# Design, synthesis, in vitro anticancer, molecular docking and SAR studies of new series of pyrrolo[2,3-*d*]pyrimidine derivatives

**DOI:** 10.1186/s13065-023-01014-0

**Published:** 2023-08-28

**Authors:** Farid M. Sroor, Wael M. Tohamy, Khairy M. A. Zoheir, Nagwa M. Abdelazeem, Karima F. Mahrous, Nada S. Ibrahim

**Affiliations:** 1 Organometallic and Organometalloid Chemistry Department, National Research Centre, Cairo, 12622 Egypt; 2 Cell Biology Department, National Research Centre, Dokki, 12622 Egypt; 3 Department of Chemistry (Biochemistry Branch), Faculty of Science, Cairo University, Giza, Egypt

**Keywords:** Pyrrolo[2,3-*d*]pyrimidine, Anti-cancer, In-vitro, In-silico, Single-crystal X-ray diffraction, SAR

## Abstract

The current study involves the design and synthesis of a newly synthesized pyrrolo[2,3-*d*]pyrimidine derivatives to contain chlorine atoms in positions 4 and 6 and trichloromethyl group in position 2 using microwave technique as a new and robust approach for preparation of this type of pyrrolo[2,3-*d*]pyrimidine derivatives. The chemical structure of the synthesized pyrrolo[2,3-*d*]pyrimidine derivatives **3**–**19** was well-characterized using spectral and elemental analyses as well as single-crystal X-ray diffraction. All compounds were tested in vitro against seven selected human cancer cell lines, namely, MCF7, A549, HCT116, PC3, HePG2, PACA2 and BJ1 using MTT assay. It was found that compounds **14a**, **16b** and **18b** were the most active toward MCF7 with IC_50_ (1.7, 5.7, and 3.4 μg/ml, respectively) relative to doxorubicin (Dox.) (26.1 μg/ml). Additionally, compound **17** exerted promising cytotoxic effects against HePG2 and PACA2 with IC_50_ (8.7 and 6.4 μg/ml, respectively) relative to Dox. (21.6 and 28.3 μg/ml, respectively). The molecular docking study confirmed our ELISA result which showed the promising binding affinities of compounds **14a** and **17** against Bcl2 anti-apoptotic protein. At the gene expression level, *P53, BAX, DR4* and *DR5* were up-regulated, while *Bcl2, Il-8,* and *CDK4* were down-regulated in **14a**, **14b** and **18b** treated MCF7 cells. At the protein level, compound **14b** increased the activity of Caspase 8 and BAX (18.263 and 14.25 pg/ml) relative to Dox. (3.99 and 4.92 pg/ml, respectively), while the activity of Bcl2 was greatly decreased in **14a** treated MCF7 (2.4 pg/ml) compared with Dox. (14.37 pg/ml). Compounds **14a** and **14b** caused cell cycle arrest at the G1/S phase in MCF7. Compounds **16b** and **18b** induced the apoptotic death of MCF7 cells. In addition, the percentage of fragmented DNA was increased significantly in **14a** treated MCF7 cells.

**Supplementary Information:**

The online version contains supplementary material available at 10.1186/s13065-023-01014-0.

## Introduction

Cancer or tumor cells are known as abnormal growth of cells in the body. According to ACS (American Cancer Society), cancer was estimated to cause 1700 deaths daily and causes about 15% of all human deaths worldwide [1, 2]. As reported in the GLOBOCAN 2020, female breast cancer (with around 2.3 million new cases, 11.7%) surpassed lung tumor (11.4%) as the most commonly diagnosed cancer, followed by colorectal (10.0%), prostate (7.3%) [3]. By 2030, it is predicted that pancreatic cancer will be in the second stand of cancer-related death after lung tumors [4].

Apoptosis is a cellular death mechanism that plays a critical role in both physiological and pathological conditions. It has a great impact on cellular development and homeostasis [5]. It serves to remove any unwanted cells and is a highly regulated mechanism. DNA damage or uncontrolled proliferation triggers the activation of the apoptotic pathway [5]. Both intracellular and extracellular signals activate the apoptotic pathway. According to the type of signal, apoptosis divides into two pathways (intrinsic and extrinsic). They are also called the mitochondrial and death receptor pathways, respectively. The intracellular signals involve growth factor deficiency; cytokine deficiency and DNA damage [6], while the extracellular signals are death signals induced by cytotoxic T cells in response to infected or damaged cells [6]. Caspases (cysteine aspartyl-specific proteases) are a family of cysteine proteins that degrade specific proteins resulting in apoptosis [7]. The caspases are classified into four initiators (caspase-2, -8, -9, -10) and three executioners (caspase-3, -6, -7) [7]. The role of the executioner caspases is to degrade the target proteins leading to cell death. Apoptosis evasion, angiogenesis and uncontrolled growth are the most common hallmarks of cancer that are present in all cancer cell types [8]. One of the main functions of apoptosis is to prevent cancer [8]. The control of restoring or terminating uncontrolled growth by using the apoptotic process is a highly effective cancer treatment method. Therefore, targeting apoptosis is effective for all cancer types. Many anticancer drugs target various stages in both the intrinsic and extrinsic pathways [5].

The use of chemotherapy in the treatment of tumors has opened new possibilities for improving the quality of life of cancer patients and for the cure of disease. Chemotherapeutic drugs are cytotoxic and can kill both normal and malignant cell types. Their usefulness in the treatment of malignancy relies on the assumption that cancer cells, which are rapidly proliferating cells, take up extracellular materials at a higher rate than normal cells [9, 10].

Due to the ability of the nitrogen-containing heterocycles to create hydrogen bonding, van der Waals forces, hydrophobic effects, π-stacking interactions and dipole–dipole interactions with the biological targets, there more than 80% of the marketed drugs approved by the FDA [11–15]. The pyrrolopyrimidine derivatives are nitrogen-fused heterocycles in which they commonly display a wide array of biological and pharmacological properties and they are found in various small molecule drug design programs [16].

The pyrrolo[2,3-*d*]pyrimidine derivatives have an important place amongst the pyrrolopyrimidine compounds with various biological properties such as antibacterial [17], anti-diabetic agents [18, 19], antiviral [20, 21], anti-inflammatory [22, 23], anti-hypertensive activity [24], anti-protozoal activity [25], and exhibited strong anticancer activity that serves as an efficient tool for DNA interaction [4, 16, 26, 27].

**Fig. 1:**
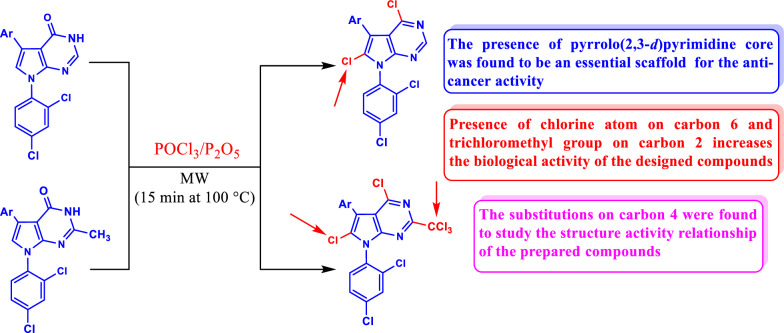
Design concept of the targeted pyrrolo[2,3-*d*]pyrimidine derivatives

In continuation with our research studies focusing on the synthesis of novel nitrogen-containing heterocycles with anti-cancer activity [12, 13, 28–32]. Recently, we have been attentive in carrying out the preparation of new pyrrolo[2,3-*d*]pyrimidine derivatives, with expected biological activity as anti-BVDV (Bovine Viral Diarrhea Virus), under environmentally friendly, time-saving microwave-assisted conditions. Accordingly, we reported that the presence of the trichloromethyl group at the 2-position and chlorine atom at the 6-position of the pyrrolo[2,3-*d*]pyrimidine scaffold increased the antiviral effect on Bovine Viral Diarrhea Virus (BVDV) [20]. Consequently, as depicted in Fig. 1, we used our robust method using microwave-assisted conditions to prepare a new series of pyrrolo[2,3-*d*]pyrimidine derivatives having the trichloromethyl group on carbon 2 and chlorine atom on carbon 6 with different substitutions on carbon 4 to evaluate the anti-cancer activity of these compounds as a first study for this type of pyrrolo[2,3-*d*]pyrimidines as anti-cancer agents. Our target is to study the mechanism of action of our compounds as anticancer agents through studying the effect on cell cycle and apoptosis at the gene, protein and DNA level using RT-PCR, Eliza and DNA fragmentation assays, respectively.

In an effort made to find a new pyrrolo[2,3-*d*]pyrimidine derivatives with promising anti-cancer activities, twenty-five derivatives were synthesized in this study and their relative anti-cancer activities were investigated in an in vitro study. In addition, the molecular docking study and the structure–activity-relationship (SAR) were presented and discussed.

## Materials and methods

### Chemistry

Digital Gallen Kamp MFB-595 instrument was used for recording the melting points for all the compounds and may be uncorrected. The chemicals of 4-Bromoacetophenone, 3,4-Dimethoxyacetophenone, hydrazine monohydrate, malononitrile, formic acid, POCl_3_, pyrrolidine, morpholine, and *N*-methyl piperazine were used as received without prior purification. IR spectra (cm^−1^) were recorded on a JASCO spectrophotometer using a KBr pellet. The ^1^H-NMR and ^13^C-NMR spectra were recorded on Bruker spectrometer (400 MHz and 100 MHz, respectively) in deuterated dimethyl sulfoxide (DMSO-*d*_6_). ^1^H-NMR spectra were assigned relative to deuterated solvent signals, while they reported as follows: chemical shift (*δ* ppm), multiplicity (s = singlet, d = doublet, t = triplet, m = multiplet), and coupling constant (*J* in Hz). Elemental analyses were recorded in the micro-analysis center at Cairo University. The TLC technique was used to check the purity of the newly synthesized compounds [20].

### General procedure for synthesis of 2-amino-1H-pyrrole-3-carbonitrile derivatives (2a and 2b)

Compounds **2a** and **2b** were prepared in three steps starting with acetophenone derivatives of 1-(4-bromophenyl)ethan-1-one or 1-(3,4-dimethoxyphenyl)ethan-1-one, respectively. The first step, the phenacyl bromide of the acetophenone derivatives was prepared according to the previous literature [33]. Second step, A mixture of the obtained phenacyl bromide (10.80 mmol) reacted with 2,4-dichloraniline (11.0 mmol) and NaHCO_3_ dissolved in water (10%) in ethanol (40 ml) was introduced in round flask (205 ml). The reaction mixture was heated in water bath for 2 h at 70 °C. The precipitate was filtered off and recrystallized from ethanol to afford the corresponding 2-((2,4-dichlorophenyl)amino)-1-phenylethan-1-one derivatives in excellent yield. Last step, the target pyrrole derivatives **2a** or **2b** were prepared by reaction of a mixture of 2-((2,4-dichlorophenyl)amino)-1-phenylethan-1-one derivatives (5.6 mmol) with the equivalent molar ratio of malononitrile (0.4 g, 6 mmol) in NaOEt (0.2 g Na metal in 20 ml ethanol) under reflux until the precipitate formed. The precipitate was filtered off and washed with ethanol (3 × 10 ml) to afford both **2a** and **2b** which recrystallized from ethanol in excellent yield as follows: [20]

### 2-amino-4-(4-bromophenyl)-1-(2,4-dichlorophenyl)-1H-pyrrole-3-carbonitrile (2a) [20]

Yield 98% as a yellowish solid, m.p. 234–235 °C; IR (KBr, cm^−1^): *υ* (cm^−1^) 3386, 3319 (NH_2_), 2196 (C≡N). ^1^H NMR (400 MHz, DMSO-*d*_*6*_): *δ* (ppm) 7.91 (m, 1H, Ar–H), 7.57 (m, 6H, Ar–H), 6.81 (s, 1H, CH-pyrrole), 6.12 (s, 2H, NH_2_). ^13^C NMR (100 MHz, DMSO-*d*_*6*_): *δ* (ppm) 149.7, 134.5, 133.0, 132.4, 131.4, 131.6, 129.9, 128.5, 127.0, 120.6, 119.1, 117.8, 113.3, 67.8; Anal. Calcd for C_17_H_10_BrCl_2_N_3_ (407.09): C, 50.16; H, 2.48; N, 10.32. Found: C, 50.22; H, 2.51; N, 10.45.

### 2-amino-1-(2,4-dichlorophenyl)-4-(3,4-dimethoxyphenyl)-1H-pyrrole-3-carbonitrile (2b)

Yield 87% as a brown solid, m.p. 248–250 °C; IR (KBr, cm^−1^): *υ* (cm^−1^) 3388, 3322 (NH_2_), 2191 (C≡N). ^1^H NMR (400 MHz, DMSO-*d*_*6*_): *δ* (ppm) 7.89 (s, 1H, Ar–H), 7.59 (s, 2H, Ar–H), 7.18 (m, 2H, Ar–H), 6.98 (m, 1H, Ar–H), 6.68 (s, 1H, CH-pyrrole), 6.00 (s, 2H, NH_2_), 3.78 (s, 6H, OCH_3_). ^13^C NMR (100 MHz, DMSO-*d*_*6*_): *δ* (ppm) 149.9, 149.4, 148.1, 137.4, 135.1, 133.6, 132.2, 130.5, 129.1, 126.7, 122.8, 118.8, 118.0, 112.7, 110.1, 69.2, 56.1. Anal. Calcd for C_19_H_15_Cl_2_N_3_O_2_ (388.25): C, 58.78; H, 3.89; N, 10.82. Found: C, 58.82; H, 3.79; N, 10.94.

### General procedure for preparation of 3a and 3b

The pyrrole derivatives (**2a** or **2b**) were refluxed in formic acid (25 ml, 85%) for two hours. The obtained precipitates were formed and then the reaction mixture was left for cooling to give **3a** or **3b**, respectively. The precipitate was filtered off, dried and recrystallized from ethanol to afford **3a** and **3b** as follows: [20]

### 5-(4-bromophenyl)-7-(2,4-dichlorophenyl)-3,7-dihydro-4H-pyrrolo[2,3-d]pyrimidin-4-one (3a) [20]

Yield 82% as a white solid, m.p. > 300 °C; IR (KBr, cm^−1^): *υ* (cm^−1^) 3138 (NH), 1676 (C=O), 1603 (C=N). ^1^H NMR (400 MHz, DMSO-*d*_*6*_): *δ* (ppm) 12.24 (s, 1H, NH), 7.55–8.02 (m, 9H, Ar–H). ^13^C NMR (100 MHz, DMSO-*d*_*6*_): *δ* (ppm) 158.7, 149.5, 145.2, 134.6, 133.7, 132.6, 132.5, 131.2, 130.3, 129.8, 128.4, 123.1, 119.8, 105.2; Anal. Calcd for C_18_H_10_BrCl_2_N_3_O (435.10): C, 49.69; H, 2.32; N, 9.66. Found: C, 49.78; H, 2.44; N, 9.71.

### 7-(2,4-dichlorophenyl)-5-(3,4-dimethoxyphenyl)-3,7-dihydro-4H-pyrrolo[2,3-d]pyrimidin-4-one (3b)

Yield 71% as yellow solid, m.p. 287–288 °C; IR (KBr, cm^−1^): *υ* (cm^−1^) 3137 (NH), 1676 (C=O), 1609 (C=N). ^1^H NMR (400 MHz, DMSO-*d*_*6*_): *δ* (ppm) 12.1 (s, 1H, NH), 7.51–8.10 (m, 7H, Ar–H), 6.95 (s, 1H, Ar–H), 3.78 (m, 6H, OCH_3_). ^13^C NMR (100 MHz, DMSO-*d*_*6*_): *δ* (ppm) 163.1, 158.6, 149.1, 148.2, 144.6, 134.2, 132.3, 131.5, 129.6, 128.1, 125.9, 121.6, 120.8, 120.1, 112.4, 111.6, 105.1, 78.9, 55.5, 30.6. Anal. Calcd for C_20_H_15_Cl_2_N_3_O_3_ (416.26): C, 57.71; H, 3.63; N, 10.09. Found: C, 57.88; H, 3.74; N, 9.98.

### General procedure for preparation of 4a and 4b

A mixture of pyrrolo[2,3-*d*]pyrimidin-4-one derivatives (**3a** or **3b**) and the required equivalent number of P_2_O_5_ was added in twice as much equivalents of POCl_3_. The mixture was heated in the microwave oven irradiation with 800 W at 100 °C for 15 min. After cooling, the mixture was poured onto ice water and alkalinized with a saturated potassium carbonate solution. The precipitate was filtered off and recrystallized from ethanol to afford **4a** or **4b** as follows: [20]

### 5-(4-bromophenyl)-4,6-dichloro-7-(2,4-dichlorophenyl)-7H-pyrrolo[2,3-d]pyrimidine (4a) [20]

Yield 92% as a pale yellow solid, m.p. 214–215 °C; IR (KBr, cm^−1^): *υ* (cm^−1^) 1635 (C = N). ^1^H NMR (400 MHz, DMSO-*d*_*6*_): *δ* (ppm) 8.70 (s, 1H, Ar–H), 8.06 (s, 1H, Ar–H) 7.71–7.87 (m, 4H, Ar–H), 7.54 (m, 2H, Ar–H). ^13^C NMR (100 MHz, DMSO-*d*_*6*_): *δ* (ppm) 152.2, 151.4, 150.7, 136.7, 134.3, 133.7, 133.5, 133.4, 131.6, 130.5, 129.3, 128.0, 122.4, 115.0, 112.2. MS (EI, m/z, %): ([M–(Br + 2Cl)], 235.85, 9), ([M–(Br + Cl)], 7), ([M–Cl], 451.60, 63), ([M]^−2^, 486.62, 100). Anal. Calcd for C_18_H_8_BrCl_4_N_3_ (487.99): C, 44.30; H, 1.65; N, 8.61. Found: C, 44.45; H, 1.73; N, 8.81.

### 4, 6-dichloro-7-(2,4-dichlorophenyl)-5-(3,4-dimethoxyphenyl)-7H-pyrrolo[2,3-d]pyrimidine (4b)

Yield 85% as yellow solid, m.p. 274–275 °C. ^1^H NMR (400 MHz, DMSO-*d*_*6*_): *δ* (ppm) 8.57 (s, 1H, Ar–H), 7.66–7.86 (m, 4H, Ar–H), 7.00–7.06 (m, 3H, Ar–H), 3.86 (m, 6H, OCH_3_). ^13^C NMR (100 MHz, DMSO-*d*_*6*_): *δ* (ppm) 157.1, 156.7, 136.1, 134.0, 133.9, 133.2, 132.7, 130.2, 129.9, 129.9, 128.8, 126.9, 123.4, 115.4, 114.9, 114.7, 112.7, 11.3, 61.1, 55.5. Anal. Calcd for C_21_H_14_Cl_4_N_2_O_2_ (469.14): C, 51.20; H, 2.79; N, 8.96. Found: C, 51.31; H, 2.82; N, 8.97.

### 5-(4-bromophenyl)-4-chloro-7-(2,4-dichlorophenyl)-7H-pyrrolo[2,3-d]pyrimidine (5) [20]

A (1.0 g, 2.2 mmol) of 5-(4-bromophenyl)-7-(2,4-dichlorophenyl)-3,7-dihydro-4H-pyrrolo[2,3-*d*]pyrimidin-4-one (**3a**) was refluxed in 20 ml of POCl_3_ for 18 h. The reaction mixture was left for cooling, then poured onto ice water slowly to form compound **5**. Yield 72% as a white-yellowish solid, m.p. 240–241 °C; IR (KBr, cm^−1^): *υ* (cm^−1^) 1642 (=N). ^1^H NMR (400 MHz, DMSO-*d*_*6*_): *δ* (ppm) 8.70 (m, 1H, Ar–H), 8.12 (m, 1H, Ar–H), 8.00 (m, 1H, Ar–H), 7.67–7.80 (m, 4H, Ar–H), 7.56 (s, 2H, Ar–H). ^13^C NMR (100 MHz, DMSO-*d*_*6*_): *δ* (ppm) 151.8, 151.3, 151.2, 134.8, 132.6, 132.92, 131.5, 131.2, 130.9, 130.5, 129.8, 128.4, 120.9, 115.4, 114.1. MS (EI, m/z, %): ([M–(Br + 2Cl)], 301.91, 11), ([M–Cl], 417.72, 60), ([M]^−1^, 452.67, 100). Anal. Calcd for C_18_H_9_BrCl_3_N_3_ (453.55): C, 47.67; H, 2.00; N, 9.27. Found: C, 47.71; H, 2.07; N, 9.31.

### 5-(4-bromophenyl)-7-(2,4-dichlorophenyl)-2-methyl-3,7-dihydro-4H-pyrrolo[2,3-d]pyrimidin-4-one (6) [20]

The amino pyrrole (**2a**) (3 g, 6.90 mmol) in a mixture of acetic acid and hydrochloric acid (10 ml/3 ml) was heated under reflux for 2 h. The precipitate was formed and then the reaction mixture was left for cooling to give 6 which was filtered off, dried and recrystallized from ethanol. Yield 75% as a white solid, m.p. > 300 °C; IR (KBr, cm^−1^): *υ* (cm^−1^) 2998 (NH), 1683 (C=O), 1612 (C=N); ^1^H NMR (400 MHz, DMSO-d_*6*_): *δ* (ppm) 12.10 (1H, s, NH), 7.93–7.98 (3H, m, Ar–H), 7.55–7.65 (5H, m, Ar–H) 2.28 (3H, s, CH_3_). ^13^C NMR (100 MHz, DMSO-d_*6*_): *δ* (ppm) 133.1, 132.9, 131.4, 130.4, 130.3. Anal. Calcd for C_19_H_12_BrCl_2_N_3_O (449.13): C, 50.81; H, 2.69; N, 9.36. Found: C, 50.88; H, 2.65; N, 9.43.

### 5-(4-bromophenyl)-4,6-dichloro-7-(2,4-dichlorophenyl)-2-(trichloromethyl)-7H-pyrrolo[2,3-d]pyrimidine (7) [20]

Using 5-(4-bromophenyl)-7-(2,4-dichlorophenyl)-2-methyl-3,7-dihydro-4H-pyrrolo[2,3-*d*]pyrimidin-4-one (**6**) (2 g, 0.9 mmol) and following the same procedure of synthesis the compound **4**. The precipitate was filtered off and recrystallized from ethanol to afford **7**. Yield 58% as a pale green solid, m.p. 138–140 °C; IR (KBr, cm^−1^): *υ* (cm^−1^) 1579 (C=N); ^1^H NMR (400 MHz, DMSO-*d*_*6*_): *δ* (ppm) 7.40–8.10 (7H, m, Ar–H). ^13^C NMR (100 MHz, DMSO-*d*_*6*_): *δ* (ppm) 158.3, 157.8, 151.6, 151.3, 150.6, 137.0, 133.7, 133.4, 131.7, 130.6, 130.1, 130.3, 129.5, 129.1, 128.8, 71.4; Anal. Calcd for C_19_H_7_BrCl_7_N_3_ (605.34): C, 37.70; H, 1.17; N, 6.94. Found: C, 37.72; H, 1.22; N, 6.97.

### 5-(4-bromophenyl)-6-chloro-7-(2,4-dichlorophenyl)-4-methoxy-2-(trichloromethyl)-7H-pyrrolo[2,3-d]pyrimidine (8) [20]

A mixture of 5-(4-bromophenyl)-4-chloro-7-(2,4-dichlorophenyl)-2-(trichloromethyl)-7H-pyrrolo[2,3-*d*]pyrimidine (**7**) (1 mmol) and sodium methoxide (1 mmol) was refluxed in 20 ml methanol for 2 h. After cooling, the mixture was poured onto ice water. The precipitate was filtered off and recrystallized from ethanol to afford **8**. Yield 56% as a pale yellow solid, m.p. 235–237 °C. ^1^H NMR (400 MHz, DMSO-*d*_*6*_): *δ* (ppm) 7.51 (m, 3H, Ar–H), 7.13 (m, 4H, Ar–H), 2.30 (s, 3H, OCH_3_). ^13^C NMR (100 MHz, DMSO-*d*_*6*_): *δ* (ppm) 145.5, 145.4, 144.0, 143.9, 137.8, 137.7, 128.1, 128.2, 127.6, 125.5, 125.4, 118.9, 118.8, 117.3, 116.3, 116.1, 20.8. Anal. Calcd for C_20_H_10_BrCl_6_N_3_O (600.92): C, 39.98; H, 1.68; N, 6.99. Found: C, 40.06; H, 1.80; N, 6.90.

### 5-(4-bromophenyl)-6-chloro-7-(2,4-dichlorophenyl)-4-methoxy-7H-pyrrolo[2,3-d]pyrimidine (9a) [20]

A mixture of 5-(4-bromophenyl)-2,4-dichloro-7-(2,4-dichlorophenyl)-7H-pyrrolo[2,3-*d*]pyrimidine (**4a**) (0.2 g, 0.41 mmol) and sodium methoxide (0.45 mmol) was refluxed in 10 ml methanol for 1 h. After cooling, the mixture was poured onto ice water. The precipitate was filtered off and recrystallized from ethanol to afford **9a**. Yield 83% as a pale yellow solid, m.p. 220–221 °C; IR (KBr, cm^−1^): *υ* (cm^−1^) 1550 (C=O). ^1^H NMR (400 MHz, DMSO-*d*_*6*_): *δ* (ppm) 8.45 (s, 1H, Ar–H), 8.01 (s, 1H, Ar–H) 7.65–7.78 (m, 4H, Ar–H), 7.56 (m, 2H, Ar–H), 3.98 (s, 3H, OCH_3_); ^13^C NMR (100 MHz, DMSO-*d*_*6*_): *δ* (ppm) 162.4, 152.5, 152.6, 151.9, 136.3, 134.5, 133.4, 132.7, 131.4, 130.3, 131.2, 130.6, 129.1, 121.4, 112.0, 103.4, 103.3, 54.5; Anal. Calcd for C_19_H_11_BrCl_3_N_3_O (483.57): C, 47.19; H, 2.29; N, 8.69. Found: C, 47.01; H, 2.42; N, 8.77.

### 6-chloro-7-(2,4-dichlorophenyl)-5-(3,4-dimethoxyphenyl)-4-methoxy-7H-pyrrolo[2,3-d]pyrimidine (9b)

A mixture of 2,4-dichloro-1-(2,4-dichlorophenyl)-3-(3,4-dimethoxyphenyl)-1H-pyrrolo[2,3-b]pyridine (**4b**) (0.5 mmol) and sodium methoxide (0.5 mmol) was refluxed in 10 ml methanol for 1 h. After cooling, the mixture was poured onto ice water. The precipitate was filtered off and recrystallized from ethanol to afford **9b**. Yield 51% as yellow solid, m.p. 215–216 °C. ^1^H NMR (400 MHz, DMSO-*d*_*6*_): *δ* (ppm) 8.46 (s, 1H, Ar–H), 8.03 (m, 1H, Ar–H) 7.75 (m, 2H, Ar–H), 7.25 (m, 3H, Ar–H), 3.77–4.03 (m, 9H, OCH_3_). ^13^C NMR (100 MHz, DMSO-*d*_*6*_): *δ* (ppm) 147.2, 144.0, 135.8, 135.6, 134.1, 132.9, 130.9, 130.7, 129.9, 128.7, 128.6, 127.4, 125.1, 124.9, 123.5, 123.1, 121.4, 60.3, 55.8, 53.9. Anal. Calcd for C_21_H_16_Cl_3_N_3_O_3_ (464.73): C, 56.98; H, 3.70; N, 6.04. Found: C, 57.02; H, 3.64; N, 6.12.

### 5-(4-bromophenyl)-6-chloro-7-(2,4-dichlorophenyl)-4-(pyrrolidin-1-yl)-2-(trichloromethyl)-7H-pyrrolo[2,3-d]pyrimidine (10)

A mixture of 5-(4-bromophenyl)-4-chloro-7-(2,4-dichlorophenyl)-2-(trichloromethyl)-7H-pyrrolo[2,3-*d*]pyrimidine (**7**) (0.5 mmol) and pyrrolidine (0.5 mmol) was refluxed in 30 ml ethanol for 2 h. After cooling, the mixture was poured onto ice water. The precipitate was filtered off and recrystallized from ethanol to afford **10**. Yield 52% as white solid, m.p. 220–225 °C. ^1^H NMR (400 MHz, DMSO-*d*_*6*_): *δ* (ppm) 6.96–8.03 (m, 7H, Ar–H), 3.17 (m, 4H, 2CH_2_), 1.69 (m, 4H, 2CH_2_). Anal. Calcd for C_23_H_15_BrCl_6_N_4_ (640.01): C, 43.16; H, 2.36; N, 8.75. Found: C, 43.22; H, 2.48; N, 8.82.

### 5-(4-bromophenyl)-6-chloro-7-(2,4-dichlorophenyl)-4-(pyrrolidin-1-yl)-7H-pyrrolo[2,3-d]pyrimidine (11a) [20]

A mixture of 5-(4-bromophenyl)-2,4-dichloro-7-(2,4-dichlorophenyl)-7H-pyrrolo[2,3-*d*]pyrimidine (**4a**) (0.2 g, 0.41 mmol) and pyrrolidine (0.03 g, 0.41 mmol) was refluxed in 20 ml ethanol for 2 h. After cooling, the mixture was poured onto ice water. The precipitate was filtered off and recrystallized from ethanol to afford **11a**. Yield 74% as a white-yellowish solid, m.p. 201–202 °C; IR (KBr, cm^−1^): *υ* (cm^−1^) 1588 (C=N). ^1^H NMR (400 MHz, DMSO-*d*_*6*_): *δ* (ppm) 8.14 (s, 1H, Ar–H), 7.97 (s, 1H, Ar–H), 7.69 (m, 4H, Ar–H), 7.39 (d, 2H, *J* = 8 Hz, Ar–H), 3.12 (m, 4H, 2CH_2_), 1.64 (m, 4H, 2CH_2_); ^13^C NMR (100 MHz, DMSO-*d*_*6*_): *δ* (ppm) 156.2, 152.1, 151.6, 135.8, 134.6, 133.9, 133.4, 133.0, 132.0, 130.2, 128.9, 121.6, 120.5, 112.9, 102.2, 56.5, 50.1, 25.3, 19.0. Anal. Calcd for C_22_H_16_BrCl_3_N_4_ (522.65): C, 50.56; H, 3.09; N, 10.72. Found: C, 50.42; H, 2.98; N, 11.01.

### 6-chloro-7-(2,4-dichlorophenyl)-5-(3,4-dimethoxyphenyl)-4-(pyrrolidin-1-yl)-7H-pyrrolo[2,3-d]pyrimidine (11b)

A mixture of 2,4-dichloro-1-(2,4-dichlorophenyl)-3-(3,4-dimethoxyphenyl)-1H-pyrrolo[2,3-b]pyridine (**4b**) (0.5 mmol) and pyrrolidine (0.5 mmol) was refluxed in 20 ml ethanol for 2 h. After cooling, the mixture was poured onto ice water. The precipitate was filtered off and recrystallized from ethanol to afford **11b**. Yield 45% as white solid, m.p. 198–201 °C. ^1^H NMR (400 MHz, DMSO-*d*_*6*_): *δ* (ppm) 8.12 (s, 1H, Ar–H), 7.79 (s, 1H, Ar–H), 7.54 (m, 3H, Ar–H), 7.01 (m, 2H, Ar–H), 3.89 (m, 6H, OCH_3_), 3.28 (m, 4H, 2CH_2_), 1.76 (m, 4H, 2CH_2_). ^13^C NMR (100 MHz, DMSO-*d*_*6*_): *δ* (ppm) 155.9, 154.8, 155.7, 151.3, 151.1, 149.5, 135.8, 134.3, 132.9, 131.5, 129.7, 128.4, 126.2, 113.5, 111.6, 102.0, 55.6, 49.3, 48.8, 24.8. MS (EI, m/z, %): ([M–2Cl], 432.06, 9), ([M–OMe]^+2^, 475.12, 42), ([M], 503.17, 100). Anal. Calcd for C_24_H_21_Cl_3_N_4_O_2_ (503.81): C, 57.22; H, 4.20; N, 11.12. Found: C, 57.32; H, 4.28; N, 11.22.

### 5-(4-bromophenyl)-6-chloro-7-(2,4-dichlorophenyl)-3,7-dihydro-4H-pyrrolo[2,3-d]pyrimidine-4-thione (12) [20]

A mixture of 5-(4-bromophenyl)-2,4-dichloro-7-(2,4-dichlorophenyl)-7H-pyrrolo[2,3-*d*]pyrimidine (**4a**) (0.4 g, 0.82 mmol) and thiourea (0.063 g, 0.83 mmol) was refluxed in 20 ml ethanol for 1.5 h. After cooling, the mixture was poured onto ice water. The precipitate was filtered off and recrystallized from ethanol to afford **12**. Yield 70% as a pale green solid, m.p. 178–180 °C; IR (KBr, cm^−1^): *υ* (cm^−1^) 3153 (NH), 1342 (C=S), 1587 (C=N). ^1^H NMR (400 MHz, DMSO-*d*_*6*_): *δ* (ppm) 13.66 (s, 1H, NH), 8.11 (d, 1H, *J* = 4 Hz, Ar–H), 8.02 (d, 1H, *J* = 4 Hz, Ar–H), 7.45–7.82 (m, 6H, Ar–H). ^13^C NMR (100 MHz, DMSO-*d*_*6*_): *δ* (ppm) 176.5, 145.4, 144.6, 136.4, 134.3, 134.1, 133.3, 130.9, 130.6, 130.3, 129.1, 122.8, 121.4, 117.2, 116.9; Anal. Calcd for C_18_H_9_BrCl_3_N_3_S (485.61): C, 44.52; H, 1.87; N, 8.65. Found: C, 44.63; H, 2.01; N, 8.77.

### 5-(4-bromophenyl)-6-chloro-7-(2,4-dichlorophenyl)-4-(methylthio)-7H-pyrrolo[2,3-d]pyrimidine (13) [20]

A mixture of 5-(4-bromophenyl)-2-chloro-7-(2,4-dichlorophenyl)-3,7-dihydro-4H-pyrrolo[2,3-*d*]pyrimidine-4-thione (**12**) (0.48 g, 1.0 mmol) in ethanol as solvent and sodium hydroxide (0.08 g, 2.0 mmol) with stirring for 1 h and add methyl iodide (0.06 ml, 1.0 mmol) with stirring until precipitate was formed. Product **13** was filtered off and purified by recrystallization from ethanol. Yield 42% as yellow crystals, m.p. 171–172 °C; IR (KBr, cm^−1^): *υ* (cm^−1^) 1594 (C = N). ^1^H NMR (400 MHz, DMSO-*d*_*6*_): *δ* (ppm) 8.64 (s, 1H, Ar–H), 8.03 (s, 1H, Ar–H), 7.71–7.83 (m, 4H, Ar–H), 7.46 (d, 2H, *J* = 8 Hz, Ar–H), 2.48 (s, 3H, CH_3_). ^13^C NMR (100 MHz, DMSO-*d*_*6*_): *δ* (ppm) 161.4, 152.0, 148.5, 136.3, 134.9, 133.5, 131.6, 130.4, 129.2, 130.3, 124.7, 122.4, 114.2, 112.5, 12.5; Anal. Calcd for C_19_H_11_BrCl_3_N_3_S (499.63): C, 45.68; H, 2.22; N, 8.41. Found: C, 45.30; H, 2.63; N, 9.08.

### 5-(4-bromophenyl)-6-chloro-7-(2,4-dichlorophenyl)-4-hydrazineyl-7H-pyrrolo[2,3-d]pyrimidine (14a) [20]

A mixture of 5-(4-bromophenyl)-2,4-dichloro-7-(2,4-dichlorophenyl)-7H-pyrrolo[2,3-*d*]pyrimidine (**4a**) (0.2 g, 0.41 mmol) and hydrazine monohydrate (0.02 g, 0.43 mmol) was refluxed in 20 ml ethanol for 1 h. By following the procedure of **11a**, compound **14a** was afforded as an excellent yield. Yield 86% as a bright yellow solid, m.p. 187–188 °C; IR (KBr, cm^−1^): *υ* (cm^−1^) 3412, 3265 (NH_2_), 3100 (NH), 1588 (C=N). ^1^H NMR (400 MHz, DMSO-*d*_*6*_): *δ* (ppm) 8.25 (s, 1H, Ar–H), 7.99 (s, 1H, Ar–H), 7.73 (m, 4H, Ar–H), 7.45 (d, 2H, *J* = 8 Hz, Ar–H), 6.83 (br, 1H, NH), 4.51 (br, 2H, NH_2_). ^13^C NMR (100 MHz, DMSO-*d*_*6*_): *δ* (ppm) 153.1, 149.8, 136.0, 134.5, 133.5, 132.4, 132.3, 131.6, 131.2, 130.3, 129.0, 121.8, 120.3, 112.0, 99.8, 50.1. Anal. Calcd for C_18_H_11_BrCl_3_N_5_ (483.58): C, 44.71; H, 2.29; N, 14.48. Found: C, 45.12; H, 2.75; N, 14.71.

### 6-chloro-7-(2,4-dichlorophenyl)-5-(3,4-dimethoxyphenyl)-4-hydrazineyl-7H-pyrrolo[2,3-d]pyrimidine (14b)

A mixture of 2,4-dichloro-1-(2,4-dichlorophenyl)-3-(3,4-dimethoxyphenyl)-1H-pyrrolo[2,3-b]pyridine (**4b**) (0.5 mmol) and hydrazine monohydrate (0.55 mmol) was refluxed in 20 ml ethanol for 1 h. By following the procedure of **14a**, the product **14b** afforded in excellent yield. Yield 86% as yellow solid, m.p. 200–202 °C; IR (KBr, cm^−1^): *υ* (cm^−1^) 3416, 3260 (NH_2_), 3120 (NH), 1593 (C=N). ^1^H NMR (400 MHz, DMSO-*d*_*6*_): *δ* (ppm) 10.99 (br, s, 3H, NH + NH_2_), 7.01–7.81 (m, 7H, Ar–H), 3.89 (m, 6H, OCH_3_). ^13^C NMR (100 MHz, DMSO-*d*_*6*_): *δ* (ppm) 152.5, 149.0, 148.8, 148.7, 135.3, 134.1, 132.9, 131.2, 129.8, 128.5, 123.5, 122.0, 114.7, 113.2, 112.1, 60.2, 55.6. Anal. Calcd for C_20_H_16_Cl_3_N_5_O_2_ (464.73): C, 51.69; H, 3.47; N, 15.07. Found: C, 51.73; H, 3.52; N, 15.18.

### 5-(4-bromophenyl)-6-chloro-7-(2,4-dichlorophenyl)-4-hydrazineyl-2-(trichloromethyl)-7H-pyrrolo[2,3-d]pyrimidine (15) [20]

5-(4-bromophenyl)-4-chloro-7-(2,4-dichlorophenyl)-2-(trichloromethyl)-7H-pyrrolo[2,3-*d*]pyrimidine (**7**) (0.2 g, 0.35 mmol) and hydrazine monohydrate (0.02 g, 0.43 mmol) was refluxed in 20 ml ethanol for 1 h. By following the procedure of **14a**, compound **15** is afforded in medium yield. Yield 61% as a bright yellow solid, m.p. 161–162 °C. ^1^H NMR (400 MHz, DMSO-*d*_*6*_): *δ* (ppm) 7.01–8.01 (m, 9H, NH_2_ + Ar–H), 4.84 (br, 1H, NH); ^13^C NMR (100 MHz, DMSO-*d*_*6*_): *δ* (ppm) 159.2, 157.3, 149.2, 136.2, 134.5, 134.3, 133.4, 132.3, 132.1, 131.3, 130.4, 129.4, 129.2, 112.5, 99.6, 72.6; Anal. Calcd for C_19_H_10_BrCl_6_N_5_ (600.93): C, 37.98; H, 1.68; N, 11.65. Found: C, 38.05; H, 1.71; N, 11.88.

### 5-(4-bromophenyl)-6-chloro-7-(2,4-dichlorophenyl)-4-(4-methylpiperazin-1-yl)-7H-pyrrolo[2,3-d]pyrimidine (16a)

A mixture of 5-(4-bromophenyl)-2,4-dichloro-7-(2,4-dichlorophenyl)-7H-pyrrolo[2,3-*d*]pyrimidine (**4a**) (0.5 mmol) and *N*-methyl piperazine (0.52 mmol) was refluxed in 20 ml ethanol for 2 h. By following the procedure of **11a**, compound **16a** was afforded in good yield. Yield 82% as a bright yellow solid, m.p. 175–178 °C. ^1^H NMR (400 MHz, DMSO-*d*_*6*_): *δ* (ppm) 8.32 (s, 1H, Ar–H), 7.99 (s, 1H, Ar–H), 7.72–7.75 (m, 4H, Ar–H), 7.45 (d, 2H, *J* = 8 Hz, Ar–H), 3.18 (s, 4H, CH_2_), 2.08 (s, br, 7H, CH_3_ + CH_2_). ^13^C NMR (100 MHz, DMSO-*d*_*6*_): *δ* (ppm) 159.0, 152.0, 151.9, 136.1, 134.6, 133.4, 132.3, 132.0, 131.8, 130.3, 129.1, 121.4, 112.3, 103.2, 103.1, 60.5, 54.1, 49.0, 46.1. Anal. Calcd for C_23_H_19_BrCl_3_N_5_ (551.69): C, 50.07; H, 3.47; N, 12.69. Found: C, 50.12; H, 3.55; N, 12.78.

### 6-chloro-7-(2,4-dichlorophenyl)-5-(3,4-dimethoxyphenyl)-4-(4-methylpiperazin-1-yl)-7H-pyrrolo[2,3-d]pyrimidine (16b)

A mixture of 2,4-dichloro-1-(2,4-dichlorophenyl)-3-(3,4-dimethoxyphenyl)-1H-pyrrolo[2,3-b]pyridine (**4b**) (0.5 mmol) and *N*-methyl piperazine (0.52 mmol) was refluxed in 20 ml ethanol for 2 h. By following the procedure of **16a**, product **16b** was afforded in good yield. Yield 84% as a bright yellow solid, m.p. 198–199 °C. ^1^H NMR (400 MHz, DMSO-*d*_*6*_): *δ* (ppm) 8.29–8.31 (m, 1H, Ar–H), 8.00 (s, 1H, Ar–H), 7.69–7.73 (m, 2H, Ar–H), 7.22 (s, 1H, Ar–H), 6.97–7.11 (m, 2H, Ar-H), 3.79–3.86 (m, 6H, OCH_3_), 3.20 (s, 4H, CH_2_), 2.03–2.08 (m, 7H, CH_3_ + CH_2_). ^13^C NMR (100 MHz, DMSO-*d*_*6*_): *δ* (ppm) 158.5, 154.1, 152.9, 148.2, 146.2, 135.5, 134.4, 132.9, 131.3, 130.3, 131.5, 129.7, 128.8, 124.8, 122.4, 121.8, 112.8, 102.0, 60.2, 56.1, 53.6, 48.2, 45.6. Anal. Calcd for C_25_H_24_Cl_3_N_5_O_2_ (532.85): C, 56.35; H, 4.54; N, 13.14. Found: C, 56.22; H, 4.52; N, 13.28.

### 5-(4-bromophenyl)-6-chloro-7-(2,4-dichlorophenyl)-4-(4-methylpiperazin-1-yl)-2-(trichloromethyl)-7H-pyrrolo[2,3-d]pyrimidine (17)

A mixture of 5-(4-bromophenyl)-4-chloro-7-(2,4-dichlorophenyl)-2-(trichloromethyl)-7H-pyrrolo[2,3-*d*]pyrimidine (**7**) (0.5 mmol) and *N*-methyl piperazine (0.52 mmol) was refluxed in 20 ml ethanol for 2 h. By following the procedure of **16b**, compound **17** was afforded in good yield. Yield 82% as a bright yellow solid, m.p. 175–178 °C. ^1^H NMR (400 MHz, DMSO-*d*_*6*_): *δ* (ppm) 7.95 (s, 1H, Ar–H), 7.69 (m, 4H, Ar–H), 7.55 (m, 2H, Ar–H), 3.15 (m, 4H, CH_2_), 2.23 (s, 3H, CH_3_), 2.04 (m, 4H, CH_2_). ^13^C NMR (100 MHz, DMSO-*d*_*6*_): *δ* (ppm) 158.4, 155.9, 149.2, 136.1, 134.2, 133.4, 133.3, 132.8, 132.3, 131.1, 130.4, 130.5, 129.1, 121.0, 117.7, 115.5, 103.5, 54.8, 25.1, 21.4. Anal. Calcd for C_24_H_18_BrCl_6_N_5_ (669.05): C, 43.09; H, 2.71; N, 10.47. Found: C, 43.15; H, 2.87; N, 10.55.

### 4-(5-(4-bromophenyl)-6-chloro-7-(2,4-dichlorophenyl)-7H-pyrrolo[2,3-d]pyrimidin-4-yl)morpholine (18a)

A mixture of 5-(4-bromophenyl)-2,4-dichloro-7-(2,4-dichlorophenyl)-7H-pyrrolo[2,3-*d*]pyrimidine (**4a**) (0.5 mmol) and morpholine (0.52 mmol) was refluxed in 20 ml ethanol for 2 h. By following the procedure of **11a**, compound **16a** was afforded in good yield. Yield 82% as a bright yellow solid, m.p. 175–178 °C. ^1^H NMR (400 MHz, DMSO-*d*_*6*_): *δ* (ppm) 8.36 (s, 1H, Ar–H), 7.99 (s, 1H, Ar–H), 7.74 (m, 4H, Ar–H), 7.48 (m, 2H, Ar–H), 3.35 (m, 4H, CH_2_), 3.16 (s, 4H, CH_2_). ^13^C NMR (100 MHz, DMSO-*d*_*6*_): *δ* (ppm) 159.1, 152.3, 152.0, 151.9, 136.1, 134.5, 133.4, 132.3, 132.2, 132.1, 131.8, 131.5, 130.3, 129.1, 122.6, 112.2, 103.3, 65.7, 49.7. Anal. Calcd for C_22_H_16_BrCl_3_N_4_O (538.65): C, 49.06; H, 2.99; N, 10.40. Found: C, 49.12; H, 3.05; N, 10.48.

### 4-(6-chloro-7-(2,4-dichlorophenyl)-5-(3,4-dimethoxyphenyl)-7H-pyrrolo[2,3-d]pyrimidin-4-yl)morpholine (18b)

A mixture of 2,4-dichloro-1-(2,4-dichlorophenyl)-3-(3,4-dimethoxyphenyl)-1H-pyrrolo[2,3-b]pyridine (**4b**) (0.5 mmol) and morpholine (0.52 mmol) was refluxed in 20 ml ethanol for 2 h. By following the procedure of **11a**, **18b** was afforded in good yield. Yield 72% as a bright yellow solid, m.p. 179–180 °C. ^1^H NMR (400 MHz, DMSO-*d*_*6*_): *δ* (ppm) 8.35 (s, 1H, Ar–H), 8.00 (s, 1H, Ar–H), 7.72 (m, 2H, Ar–H), 7.15 (m, 3H, Ar–H), 3.86 (m, 6H, OCH_3_), 3.38 (m, 4H, CH_2_), 3.22 (s, 4H, CH_2_). ^13^C NMR (100 MHz, DMSO-*d*_*6*_): *δ* (ppm) 151.4, 151.2, 148.5, 135.4, 134.1, 132.9, 131.2, 129.8, 128.5, 124.5, 113.4, 111.9, 110.2, 107.6, 103.2, 65.5, 55.1, 55.5, 49.2, 48.9. MS (EI, m/z, %): ([M–(OMe + 2Cl]^+2^, 415.04, 7), ([M–2Cl]^+2^, 446.06, 8), ([M–OMe], 488.17, 30), ([M], 519.14, 100). Anal. Calcd for C_24_H_21_Cl_3_N_4_O_3_ (519.81): C, 55.46; H, 4.07; N, 10.78. Found: C, 55.61; H, 4.09; N, 10.91.

### 4-(5-(4-bromophenyl)-6-chloro-7-(2,4-dichlorophenyl)-2-(trichloromethyl)-7H-pyrrolo[2,3-d]pyrimidin-4-yl)morpholine (19)

A mixture of 5-(4-bromophenyl)-4-chloro-7-(2,4-dichlorophenyl)-2-(trichloromethyl)-7H-pyrrolo[2,3-*d*]pyrimidine (**7**) (0.5 mmol) and morpholine (0.52 mmol) was refluxed in 20 ml ethanol for 2 h. By following the procedure of **18a**, compound **19** was afforded in good yield. Yield 79% as a bright yellow solid, m.p. 185–188 °C. ^1^H NMR (400 MHz, DMSO-*d*_*6*_): *δ* (ppm) 7.09–8.03 (m, 7H, Ar–H), 3.63 (m, 4H, CH_2_), 3.15 (m, 4H, CH_2_). ^13^C NMR (100 MHz, DMSO-*d*_*6*_): *δ* (ppm) 159.2, 158.1, 148.5, 134.6, 134.5, 133.4, 132.3, 132.1, 132.0, 130.5, 129.3, 129.2, 124.5, 113.2, 72.4, 65.7, 49.6, 44.5. Anal. Calcd for C_23_H_15_BrCl_6_N_4_O (656.00): C, 42.11; H, 2.30; N, 8.54. Found: C, 42.21; H, 2.41; N, 8.49.

### Single crystal X-ray diffraction

Single Crystals suitable for X-ray diffraction analysis were obtained from a saturated acetonitrile solution at room temperature. The X-ray crystal structures were determined by using a Rigaku R-AXISRAPID diffractometer and Bruker X8 Prospector. The collection of single crystal data was made at room temperature by using Cu-Kα radiation. The structures were solved by using direct methods and expanded using Fourier techniques. Thenon-hydrogen atoms were refined anisotropically [20, 34].

The structure was solved and refined using the Bruker SHELXTL Software Package, using the space group C 2/c, as monoclinic with Z = 8 for the formula unit, C_20_H_13_Cl_4_N_3_O_2_ and the formula weight 469.13. The final anisotropic full-matrix least-squares refinement on F2 with 200 variables converged at R1 0.0959%, for the observed data and wR2 = 0.2776% for all data. The goodness-off was 1.059. The largest peak in the final difference electron density synthesis was 2.084 e − /A3 and the largest hole was − 0.743 e−/A3 with an RMS deviation of 0.098 e−/A3. Based on the final model, the calculated density was 1.473 g/cm3 and F(000), 1904 e−. The CCDC number is 2215014. More details could be found in Additional file 1.

### Anticancer activity

#### MTT assay

3-(4,5-dimethylthiazol-2-yl)-2,5-diphenyl tetrazolium bromide (MTT) assay was performed to evaluate the percentage of the viability of cells under the effect of the synthesized compounds [35–37]. All of the following steps were done in a sterile Laminar flow class II biosafety cabinet (Baker, SG406INT, Sanford, ME, USA). DMEM was used as a suspension medium for breast cancer (MCF7), colorectal cancer (HCT116), prostate cancer (PC3), liver cancer (HePG2), pancreatic cancer (PACA2) and lung cancer (A549), while normal cell line (BJ1) was suspended in DMEM-F12 medium. The selected cell lines were purchased from American Type Culture Collection (ATCC). The media were supplemented with a 1% antimycotic-antibiotic mixture (10,000 µg/ml streptomycin sulfate, 25 µg/ml Amphotericin B, and 10,000 U/ml Potassium Penicillin) and 1% l-glutamine. 10 × 10^4^cells/well were seeded in 96 well microtiter plates and incubated for 24 h at 37 ℃ under 5% carbon dioxide using a water-jacketed Carbon dioxide incubator (Sheldon, SCO5A, OR, USA). Then, the media were discarded and replaced with a fresh one (without serum), and the cells were incubated either alone (negative control) or with different concentrations of the prepared compounds to give a final concentration of (100- 50- 25- 12.5- 6.25- 3.125- 1.56 and 0.78 µg/ml). After the incubation time of 72 h, the medium was removed. Afterward, 40 µl MTT salt (2.5 μg/ml) was added to each well and kept for 4 h under the same conditions of 37 ºC and 5% carbon dioxide. Finally, 200 μl of 10% Sodium dodecyl sulfate (SDS) in deionized water was added to each well and incubated overnight at 37 ºC. The step of SDS addition was done to stop the reaction and dissolve the formed formazan crystals. The absorbance was then measured at a wavelength of 595 nm and the reference wavelength was set at 620 nm using a microplate multi-well reader (Bio-Rad Laboratories Inc., model 3356, California, USA). Doxorubicin was used as a positive control that gave 100% lethality at a concentration of 100 µg/ml under the same conditions. A statistical significance analysis was determined between the samples and the negative control using an independent t-test by SPSS 11 program. Dimethyl sulfoxide (DMSO) was the vehicle that dissolved our synthesized compounds and its concentration in the cells was less than 0.2%. IC_50_ values were calculated using the GraphPad Prism 6 program. The degree of selectivity of the synthetic compounds was expressed as SI = IC_50_ of a pure compound in a normal cell line/IC_50_ of the same pure compound in the cancer cell line, where IC_50_ is the concentration of the compound required to kill 50% of the cells.

#### Molecular docking study

Molecular modeling studies for compounds **14a** and **17** were done according to Fathi et al. [38] using (MOE) program 2009.10 version to investigate the ligand–protein interactions at the active sites of the P53 mutant Y220C and Bcl2 proteins. The x-ray crystallographic structures of selected proteins were downloaded from the protein data base (PDB) (www.rcsb.org) (PDB ID: 5O1H and 6QGG respectively). The selected proteins were first prepared for modeling study where the standard ligand molecule was removed from the active site of the protein, the heavy atoms were kept fixed, and the hydrogen atoms were added to the whole protein structure. According to the author's instructions, the RMS gradient was adjusted at 0.01 kcal/mole, the RMS distance at 0.1 Å, and the partial charges were computed using the MMFF94x force field. At the final step, the ligand interaction (MOE) structure was saved as a Pdb file which was then visualized through the BIOVIA Discovery Studio V6.1.0.15350 program, where the tested compounds appeared to fit into the active domain of proteins in 2D and 3D states [39].

### Isolation of total RNA and RT-PCR

All of the extractions were conducted on ice with ice-cold reagents. Total RNA from the different cell lines was isolated using Trizol (Invitrogen; Life Technologies, USA) according to the method of Sthoeger et al. [40]. The 260:280 ratios were measured to determine RNA quality after the isolation method was completed according to the manufacturer’s instructions. A high-capacity cDNA reverse transcription kit was used to produce complementary DNA (cDNA) (Applied Biosystems, USA). Table 1 shows the primers that were used in these tests. The relative gene expression method (i.e., ΔΔCT) was used to analyze the real-time PCR data, as explained in Applied Biosystem User Bulletin No. 2. Each sample and gene were normalized using the β-actin gene.

**Table 1:** Primers sequences

Gene	Primer sequence	GenBank (Accession no)
P53 (Tumor protein)	GGCCCACTTCACCGTACTAA GTGGTTTCAAGGCCAGATGT	AH002919.2
CDK4 (cyclin-dependent kinases)	TGTATGGGGCCGTAGGAACC GCAGGGATACATCTCGAGGC	NM_000075.4
BAX (Bcl-2 Associated X-protein)	CTGGATCCAAGACCAGGGTG CCTTTCCCCTTCCCCCATTC	NR_027882.2
Bcl2 (B-cell lymphoma 2)	CCTTTGTGGAACTGTACGGC CCGGCCAACAACATGGAAAG	NM_000633.3
IL-8	ATGACTTCCAAGCTGGCCGT CCTCTTCAAAAACTTCTCCACACC	LC461682.1
Homo sapiens TNF superfamily member 10 (TNFSF10), DR4 (TRAIL1)	TTGGGACCCCAATGACGAAG TGGTCCCAGTTATGTGAGCTG	NM_003810.4
Homo sapiens TRAIL receptor 2 mRNA, (DR5)	CACGAGCGGAGAACCCC TTTTGTTGTGGGGCCACTCT	AF016266.1
Homo sapiens caspase 3 (CASP3), transcript variant 1,	TCTGGTTTTCGGTGGGTGTG GTCGGCCTCCACTGGTATTT	NM_004346.4
Homo sapiens beta-actin mRNA	GGCTCTTTTCCAGCCTTCCT AATGCCAGGGTACATGGTGG	HQ154074.1

### ELISA

Elisa assay was used to determine the concentrations of Human caspases-3, -8, Bax, and Bcl2 in PACA2- and A549-treated cells. Also, the concentrations of caspase-8, Bax, and Bcl2 in MCF7-treated cells were detected [41–44]. The procedures were followed up according to the instructions described in the following kits; Invitrogen human Caspase-3 Elisa Kit, Catalog KHO1091, DRG^®^ human Caspase-8 ELISA Kit, Catalog (EIA-4863), Human Bax ELISA Kit (ab199080) and Human Bcl-2 ELISA Kit (ab119506) respectively. Shortly, the protocol described in the above kits was as followed; at the beginning, all the reagents, samples and standards were prepared. 100 μl of samples or standard were micro pipetted to each well of micro-well strip and kept at room temperature for 2 h. After washing the micro-well strips three times with washing buffer, the prepared antibody (anti-rabbit-IgG-HRP) at a concentration of 100 μl was added to each well at room temperature for 1 h. Then, the micro-well strips were washed again three times. Afterward, the prepared tetramethyl-benzidine (TMB) solution (100 μl) was added to the micro-well strips and kept for 10 min. At last, about 50 μl of stop solution was pipetted to each well to inactivate the enzyme completely. The absorbance of all samples was measured at a wavelength of 460 nm and compared to that of the standard. Curve fitting software was used to plot a standard curve and the concentrations for the unknown samples were measured from the standard curve.

### Flow cytometer analysis of cell cycle and apoptosis

For cell cycle analysis, 10^6^ MCF7 cells were cultured in 60 mm Petri dishes for 24 h and then treated with the selected compounds **14a** and **14b** at their IC_50_ concentrations for 24 h. Herein, the negative control was the untreated MCF7 cells and Doxorubicin was used as a positive control. Firstly, after the treatment time of 24 h, MCF7 cells were centrifuged at 1000 rpm for 5 min at 4 °C. The supernatant was discarded and the cells pellet were washed in phosphate-buffered saline (PBS). Then, the cells pellet were centrifuged at 1000 rpm for another 5 min. Afterward, the cells were collected in a single-cell suspension and maintained in 70% ethanol overnight on ice. Then, cells were washed with 1 ml PBS. At last, 200 µl 1 × propidium iodide (PI) mixture was added to the cells pellet in the dark at room temperature for 30 min. Then, the DNA content was analyzed by subjecting the cells to an Epics XL-MCL flow cytometer (Beckman Coulter, Miami, FL). The distribution of cells at different phases of the cell cycle was determined by Multi-cycle software (Phoenix Flow Systems, San Diego, CA).

The percentage of apoptotic cells was detected by using Annexin V-FITC kit catalog number (#K101-25). About 2X10^6^ of MCF7 and Paca2 cells were washed in 500 µl of 1 × PBS. Then, the cells were centrifuged and the supernatant was discarded. The cells pellet were re-suspended in the annexin V incubation reagent. The annexin V incubation reagent contained 10 μl binding buffer (10 ×), 10 μl propidium iodide, 1 μl annexin V-FITC, and 79 μl deionized water. The cells were incubated in 100 μl annexin V incubation reagent for 15 min in the dark at room temperature [45]. Finally, the apoptotic cell percentage was detected by flow cytometry using the FITC signal detector (usually FL-1) and PI staining by the phycoerythrin emission signal detector (usually FL-2).

### DNA fragmentation assay in a pancreatic cell line (Paca2), breast cancer cell line (MCF-7) and lung cell line (A549)

#### DNA gel electrophoresis laddering assay

The DNA fragmentation assay in a pancreatic cell line (Paca2), breast cancer cell lines (MCF-7) and lung cell line (A549) was performed in concordance with the premises established by Yawata [46] with some modifications. Briefly, after 24 h of exposure of Paca2, MCF-7 and A549 cancer cell lines to the prepared compounds in different Petri dishes (60 × 15 mm, Greiner), the cells were suspended and homogenized in 1 ml of medium and then centrifuged for 10 min at 900 rpm. The genomic DNA was extracted as shown in Yawata [46]. About 1 × 10^6^ cells of each tested cancer cell line were treated with the IC_50_ values of the tested compounds. All the cells were collected via trypsinization and washed with Dulbecco`s Phosphate Buffered Saline. Then, the cells were treated with the lysis buffer containing 5 mM ethylenediaminetetraacetic acid (EDTA), 10 mM Tris (pH 7.4), 0.5% Triton X-100 and 150 mM NaCl for 35 min on ice. After that, the lysates were vortexed and centrifuged for 20 min at 10.000 xg. The fragmented DNA was extracted from the supernatant with an equal volume of isoamyl alcohol: chloroform: neutral phenol mixture (1:24:25). Finally, the percentage of fragmented DNA was determined by performing gel electrophoresis using 2% agarose gel containing 0.1 μg/ml ethidium bromide.

#### Diphenylamine reaction procedure

Pancreatic cancer cell line (Paca2), breast cancer cell line (MCF-7) and lung cancer cell line (A549) were used to determine the percentage of DNA fragmentation after the treatment with IC_50_ of the tested compounds. The selected cells were suspended in 0.5 ml of lysis buffer containing 1 mM EDTA, 10 mM Tris–HCl (pH 8) and 0.2% triton X-100. Then, the cells were centrifuged at 10.000 rpm for 20 min at 4 °C. After removing the supernatant, the pellets were re-suspended in 0.5 ml of lysis buffer. Then, 0.5 ml of 25% tri-chloroacetic acid (TCA) was added to the pellets (P) and the supernatants (S), and incubated for 24 h at 4 °C. Afterwards, the cells were centrifuged at 4 °C for 25 min at 10,000 rpm and the pellets were suspended in 80 ml of 5% TCA, followed by incubation at 80 °C for 20 min. Finally, 160 ml of Diphenyl Amine (DPA) solution [150 ml of sulfuric acid, 150 mg DPA in 10 ml glacial acetic acid, and 50 ml acetaldehyde (16 mg/ml)] was added to each cell sample and kept at room temperature for 24 h [47]. The percentage of fragmented DNA was measured from the absorbance reading at 600 nm wavelength using the following formula:

% Fragmented DNA = [OD(S)/[OD(S) + OD(P)] × 100 (OD: optical density, S: supernatant, P: pellet).

### Statistical analysis

All data were analyzed using the General Liner Models (GLM) procedure of Statistical Analysis System (1982)[48] followed by Scheffé-test to assess significant differences between groups. The values are expressed as mean ± SEM. All statements of significance were based on the probability of P < 0.05.

## Results and discussion

### Chemistry

**Scheme 1:**
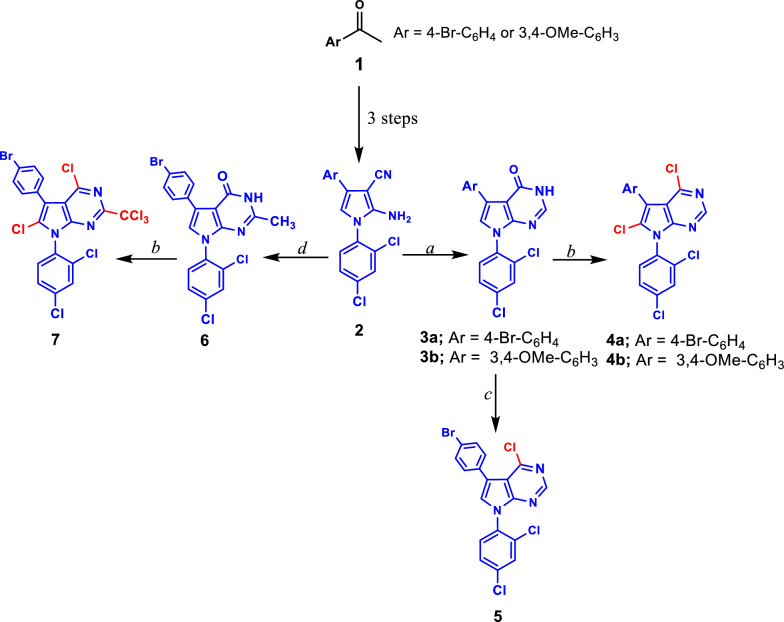
Synthesis of **3**–**7**. **a** HCOOH; **b** POCl_3_/P_2_O_5_, MW for 15 min at 100 °C; **c** POCl_3_; **d** AcOH/HCl (3:1)

The preparation of the targeted pyrrolopyrimidine derivatives **3**–**19** was started with the synthesis of cyclic pyrrole derivatives **2**, which were prepared in three steps starting with acetophenone derivatives **1** as reported in the previous works of literature [20, 33, 49]. The pyrrolo[2,3-*d*]pyrimidin-4-ones **3** or **6** were prepared via the reaction of **2** with formic acid to give pyrrolopyrimidine derivatives **3a** and **3b**, while compound **6** was obtained via the reaction of **2** with the mixture of acetic acid and hydrochloric acid in molar ratio 3:1 (Scheme 1).

The chlorination of pyrrolo[2,3-*d*]pyrimidin-4-one **3a** was carried out in two ways. Refluxing of **3a** in an excess of POCl_3_ for around 18 h afforded **5** in moderate yield 72% in which the chlorination occurred on carbon-4 only. On the other hand, the microwave technique (MW) was used in the chlorination of **3a** under the reaction condition of the presence of POCl_3_/P_2_O_5_ for 15 min at 100 °C to afford **4a** in which the chlorination occurred on the carbons 4 and 6 in excellent yield 92%. From these results we observed that microwave irradiation reduced the reaction time of the chlorination. Moreover, the heating using the microwave is more efficient for producing pure compounds in good to excellent yields in comparison to the refluxing conditions. Therefore, we have chlorinated both of the pyrrolo[2,3-*d*]pyrimidin-4-ones **3b** and **6** using the microwave technique to afford the newly synthesized **4b** and **7**, respectively, as shown in Scheme 1.

**Scheme 2:**
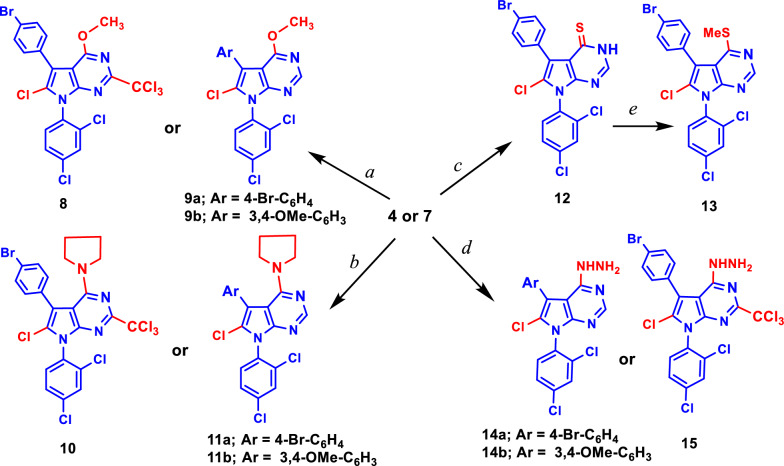
Synthesis of **8**–**15**. **a** NaOMe/MeOH; **b** Pyrrolidine/EtOH; **c** Thiourea/EtOH; **d** NH_2_NH_2_/EtOH; **e** NaOH/EtOH, MeI

Patrice Vanelle and co-workers reported that the insertion of the trichloromethyl group in position 2 of the quinazoline derivatives increased the antiplasmodial activity of these compounds [50–52]. To study the structure–activity relationship (SAR), we prepared a new series of pyrrolo[2,3-*d*]pyrimidine derivatives with different substitutions on carbon 4 and chlorine on carbon 6 with or without trichloromethyl group at carbon 2 as shown in Scheme 2. Moreover, in the current work, we reported the first study for using these compounds as anti-cancer agents. The reaction of **4** and **7** with sodium methoxide in methanol afforded **8** with the trichloromethyl group in position 2, methoxy group in position 4 and chlorine atom in position 2, while in **9** the methoxy group in position 4 and chlorine atom in position 2. Likewise, the chlorine atom in position 4 of compounds **4** and **7** was replaced with pyrrolidine rings in **10** and **11** by refluxing the pyrrolidine with **4** or **7** in ethanol, respectively (Scheme 2). The treatment of **4a** with thiourea in refluxing ethanol afforded **12** which reacted with MeI in ethanol and the presence of NaOH to give **13**. Hydrazine monohydrate reacted with both **4** and **7** in ethanol to afford the corresponding pyrrolo[2,3-*d*]pyrimidine derivatives **14** and **15** as depicted in Scheme 2.

**Scheme 3:**
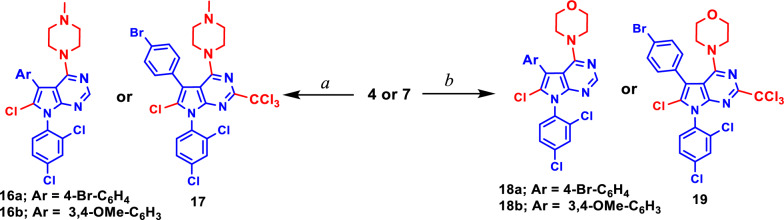
Synthesis of **16**–**19**. **a** *N*-methyl piperazine/EtOH; **b** morpholine/EtOH

Likewise, the treatment of **4** or **7** with *N*-methyl piperazine or morpholine afforded a novel series of pyrrolopyrimidine derivatives having chlorine atoms at carbon 6 in compounds **16** and **18** (Scheme 3) and pyrrolo[2,3-*d*]pyrimidine derivatives with trichloromethyl group in position 2 and chlorine atom in position 6 as shown the compounds **17** and **19** (Scheme 3).

The chemical structures of the newly synthesized pyrrolo[2,3-*d*]pyrimidine derivatives **3**–**19** were proven based on analytical and spectral data as well as single-crystal X-ray diffraction. The IR spectra of compounds **3–19** depicted absorption bands of the newly formed C=N bond in the range of *υ* 1579–1642 cm^−1^. The NH and NH_2_ groups were assigned at *υ* 2998–3153 cm^−1^ and in the region of *υ* 3319–3322 cm^−1^, and *υ* 3386–3416 cm^−1^ for the compounds containing NH and NH_2_, respectively. The ^1^H- and ^13^C-NMR spectra of compound **4** confirmed the chlorination of the carbons at positions 4 and 6 as well as the chlorination of the methyl group at position 2 of compound **6** using the MW technique, while the chlorination occurs only on carbon 4 using the reflux condition as shown in **5**. All the protons and carbons of compounds **2**–**19** were assigned in their expected chemical shifts *δ* (ppm) as depicted in the experimental section.

**Fig. 2:**
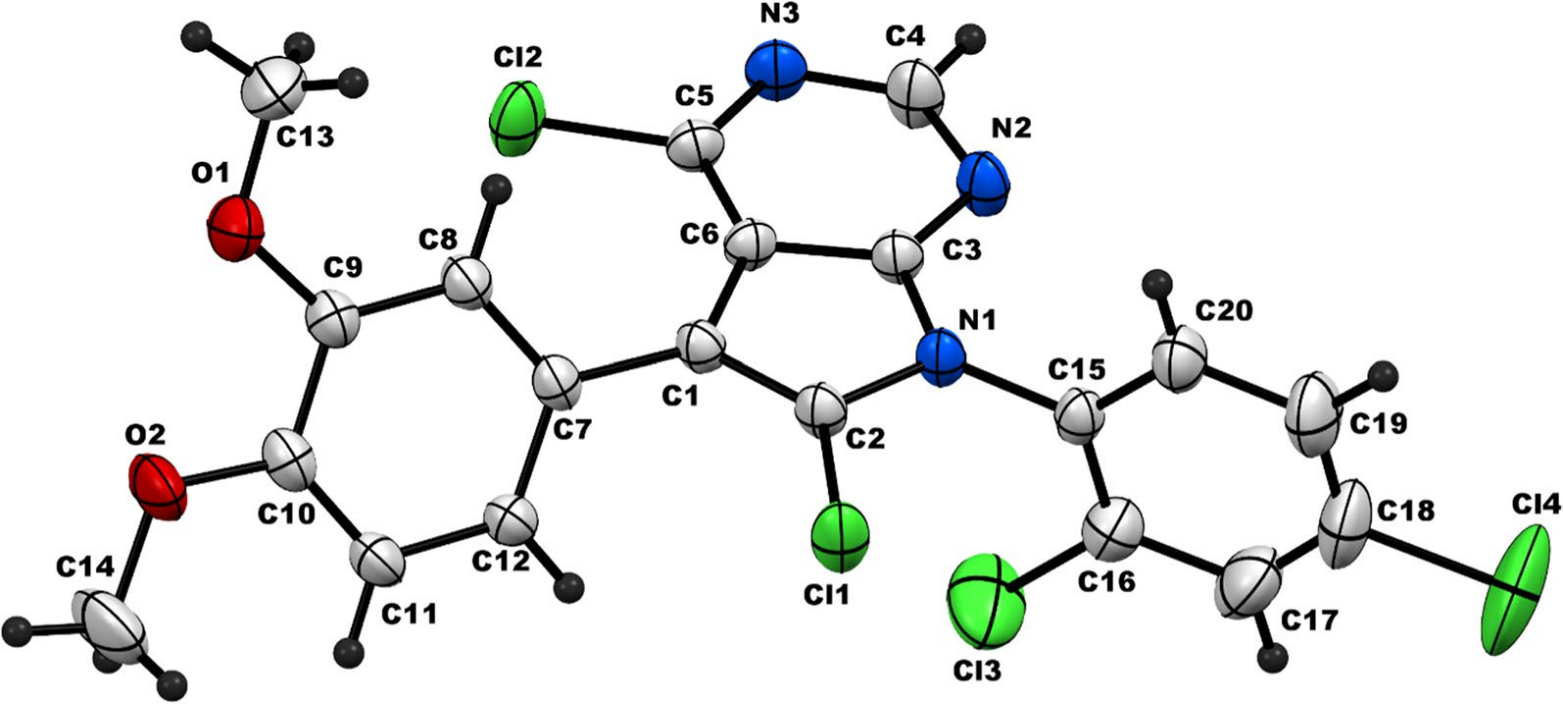
Single crystal X-ray diffraction of compound **4b**

Exclusively, good quality single crystals of **4b** were obtained from a saturated solution of acetonitrile solution at room temperature as yellow crystals and found suitable for X-ray single crystal diffraction measurement (Fig. 2). Compound **4b** crystallizes in the monoclinic space group C 2/c (see supplementary data). The crystal structure determination of **4b** confirmed the chlorination on the carbons at positions 4 and 6 of the novel 4,6-dichloro-7-(2,4-dichlorophenyl)-5-(3,4-dimethoxyphenyl)-7*H*-pyrrolo[2,3-*d*]pyrimidine (**4b**) (Fig. 2) (for more details see Additional file 1).

### Anti-cancer activity

All the synthesized compounds **3–19** were evaluated against selected human cancer cell lines for their anti-proliferative activity in an in vitro study. Seven cancer cell lines MCF7, A549, HCT116, PC3, HePG2, PACA2 and the normal cell line (BJ1) were used in this study to evaluate the cytotoxicity of the compounds **3–19** using 3-(4,5-dimethylthiazol-2-yl)-2,5-diphenyltetrazolium bromide (MTT) assay. Doxorubicin (Dox.) was used as a reference standard drug.

### Primary screening using MTT assay

The primary in vitro screening results are depicted in Table 2. The results revealed that most compounds exhibited a potent cytotoxic effect with 100% mortality against plenty of cell lines (MCF7, A549, HCT116, PC3, HePG2 and PACA2). In the case of MCF7, compounds **4a**, **6**, **7**, **9b**, **10**, **12**, **14a**, **16b** and **18b** showed 100% mortality, while compounds **4b**, **8**, **11b**, **14b** and **15** exhibited 80–95% mortality. For A549, compounds **4b**, **8**, **11b**, **14b** and **15** gave 100% mortality, while compounds **4a**, **15** and **17** exhibited 80–90% mortality. In HCT116 and PC3 cancer cell lines, they were very sensitive toward the compounds **4b**, **6**, **7**, **10**, **14a**, **14b**, **15**, **17** and **18b** with 100% cytotoxic activity. On the other hand, compounds **3a**, **4b**, **8**, **11b** and **17** exhibited 80–90% mortality against HCT116 and PC3 cancer cell lines. Compounds **8**, **14a**, **14b**, **15**, **17** and **18b** exhibited a 100% cytotoxic effect against both HePG2 and PACA2 cancer cell lines. Moreover, compounds **4a**, **4b**, **6**, **7**, **10**, and **16b** showed 100% mortality against PACA2. Finally, most of the tested compounds exhibited high safety against the human normal cell line (BJ1). Compounds **3b** (3.5%), **4b** (22.5%), **5** (16.3%), **8** (15.6), **13** (25.3), **14a** (30.8), **15** (4.5%), **17** (12.5%), **18a** (3.2%) and **18b** (20.6%) showed limited % mortality toward normal cells BJ1 and they were subjected to secondary screening to calculate their IC_50_ values and selectivity index.

**Table 2:** MTT cell proliferation mortality assay (%) of cancer and normal cell lines at 100 μg/ml

Compound	MCF7	A549	HCT116	PC3	HePG2	PACA2	BJ1
**3a**	63.5 ± 2.6	3.4 ± 0.2	84.3 ± 1.1	36.5 ± 0.9	100	71.3 ± 0.8	95.3 ± 1.8
**3b**	11.5 ± 0.8	8.5 ± 0.6	14.6 ± 0.1	15.6 ± 1.3	10.4 ± 1.7	76.3 ± 1.8	3.5 ± 0.9
**4a**	100	79.4 ± 1.6	74.2 ± 2.5	53.0 ± 0.7	43.5 ± 2.2	100	74.6 ± 0.6
**4b**	90.3 ± 4.9	100	90.2 ± 1.0	100	84.3 ± 0.9	100	22.5 ± 0.3
**5**	27.9 ± 1.2	25.1 ± 0.7	60.4 ± 0.9	42.3 ± 1.3	34.5 ± 2.9	29.3 ± 0.8	16.3 ± 2.0
**6**	100	ND	100	100	ND	100	84.9 ± 0.7
**7**	100	14.2 ± 0.8	100	54.2 ± 1.6	ND	100	100
**8**	84.3 ± 2.5	19.3 ± 1.2	80 ± 1.0	13.5 ± 1.5	100	100	15.6 ± 2.8
**9a**	75.2 ± 3.6	58.6 ± 1.3	52.1 ± 0.8	53.8 ± 0.3	ND	1.3 ± 0.2	37.8 ± 0.6
**9b**	100	ND	45.6 ± 3.2	ND	63.8 ± 0.6	2.5 ± 0.3	ND
**10**	100	10.2 ± 0.5	100	100	ND	100	100
**11a**	65.2 ± 1.0	11.8 ± 0.6	44.2 ± 0.8	12.5 ± 1.4	7.2 ± 1.3	84.3 ± 1.7	47.3 ± 1.3
**11b**	80.4 ± 1.8	11.6 ± 0.9	33.5 ± 0.4	94.6 ± 1.3	63.1 ± 0.5	25.6 ± 0.9	56.9 ± 0.1
**12**	100	71.3 ± 1.6	64.3 ± 1.1	3.1 ± 0.2	26.3 ± 3.4	81.5 ± 1.2	100
**13**	3.5 ± 0.6	42.6 ± 1.2	60.2 ± 1.0	32.2 ± 1.7	12.4 ± 1.9	58.8 ± 1.2	25.3 ± 0.4
**14a**	100	100	100	ND	100	100	30.8 ± 0.2
**14b**	94.9 ± 1.1	100	100	100	100	100	ND
**15**	81.2 ± 0.8	84.6 ± 0.4	100	65.9 ± 2.4	100	100	4.5 ± 0.3
**16a**	73.6 ± 2.2	3.2 ± 0.5	73.2 ± 0.8	3.8 ± 0.3	100	7.5 ± 1.1	70.5 ± 0.4
**16b**	100	100	ND	ND	ND	100	ND
**17**	74.3 ± 1.7	89.2 ± 0.4	100	79.5 ± 0.5	100	100	12.5 ± 1.7
**18a**	41.2 ± 1.5	22.7 ± 0.9	54.2 ± 1.2	2.1 ± 0.6	16.4 ± 1.6	31.0 ± 1.3	3.2 ± 1.5
**18b**	100	11.2 ± 0.6	ND	100	100	100	20.6 ± 2.1
**19**	62.3 ± 3.4	5.6 ± 0.7	35.9 ± 1.5	3.7 ± 1.1	100	31.2 ± 5.1	52.7 ± 1.4
Dox	100	100	100	100	100	100	–––-
Negative control	0	0	0	0	0	0	0

*ND* not detected

### Secondary screen

As illustrated in Table 3, the results indicated that most of the tested compounds have a promising cytotoxic effect against the tested cancer cell lines (MCF7, A549, HCT116, PC3, HEPG2, and PACA2) with IC_50_ values less than that of Doxorubicin (Dox.) as a positive reference drug. For MCF7, compounds **6**, **7**, **12**, **14a**, **14b**, **16b** and **18b** were more effective with IC_50_ values (15.2, 16.2, 11.9, 1.7, 16.8, 5.7, and 3.4 μg/ml) respectively, compared with reference drug (Dox.) with IC_50_ (26.1 μg/ml). Also, it was observed that compounds **14a**, **16b** and **18b** were more active than the other compounds against the same cell line. On the other hand, Compound **4b** was the most promising compound with the lowest IC_50_ (17.5 µg/ml) against A549 cells compared with Dox. (28.3 µg/mL). Regarding PC3 cells, compounds **10**, **14b** and **18b** with IC_50_ (11.6, 18.7 and 19.2 μg/ml) respectively, were very sensitive and had the lowest IC_50_ relative to the positive control Dox. (23.8 μg/ml). In the case of HePG2, compounds **3a**, **8**, **14b**, **17** and **19** were the most promising compounds with recorded IC_50_ values (19.2, 17.8, 17.5, 8.7 and 19.4 µg/ml, respectively) compared with Dox. as the reference drug (21.6 µg/ml). Compound **17** with IC_50_ (8.7 µg/ml) was found to be more potent against HepG2 cells than the other compounds. Both compounds **17** and **18b** were found to be more effective against PACA2 with IC_50_ (6.4 and 15 μg/ml, respectively), than Dox. (28.3 μg/ml). Consequently, compounds **4b**, **14a**, **14b**, **16b**, **17** and **18b** showed potent anti-cancer activity against MCF-7, while compounds **4b** and **17** are potent anti-cancer agents against A549 and PACA2 cell lines, respectively.

**Table 3:** IC_50_ (μg/ml) of the compounds **3**–**19**

Compound	MCF7	A549	HCT116	PC3	HePG2	PACA2
**3a**	–	–	44.5 ± 1.3	–	19.2 ± 0.6	–
**3b**	–	–	–	–	–	69.7 ± 1.3
**4a**	32.4 ± 0.8	68.9 ± 0.5	–	–	–	24.1 ± 1.0
**4b**	45.8 ± 1.0	17.5 ± 0.4	58.4 ± 0.7	28.5 ± 0.8	84.3 ± 0.7	–
**5**	–	–	–	–	–	–
**6**	15.2 ± 0.9	–	31.2 ± 0.9	31.1 ± 0.9		40 ± 1.0
**7**	16.2 ± 1.6	–	–	–	–	–
**8**	48.0 ± 1.5	–	68.1 ± 1.0	–	17.8 ± 1.3	23.5 ± 0.6
**9a**	–	–	–	–	–	–
**9b**	–	–	–	–	–	–
**10**	61.1 ± 1.4	–	26.9 ± 0.6	11.6 ± 0.8	–	–
**11a**	–	–	–	–	–	55.4 ± 1.4
**11b**	48.4 ± 0.8	–	–	27.7 ± 0.1	–	–
**12**	11.9 ± 0.9	–	–	–	–	59.7 ± 0.7
**13**	–	–	–	–	–	–
**14a**	1.7 ± 0.4	–	–	–	37.9 ± 0.9	–
**14b**	16.8 ± 0.9	–	–	18.7 ± 0.2	17.5 ± 0.8	–
**15**	42.8 ± 0.6	54.0 ± 1.7	–	–	29.1 ± 0.5	–
**16a**	–	–	–	–	–	–
**16b**	5.7 ± 0.9	–	–	–	–	–
**17**	–	34.1 ± 1.5	37.8 ± 0.4	66.8 ± 2.0	8.7 ± 0.8	6.4 ± 0.7
**18a**	–	–	–	–	–	–
**18b**	3.4 ± 0.3	–	–	19.2 ± 0.9	28.1 ± 0.4	15 ± 0.9
**19**	–	–	–	–	19.4 ± 0.8	–
Dox	26.1 ± 0.1	28.3 ± 1.3	37.6 ± 0.8	23.8 ± 0.2	21.6 ± 0.8	28.3 ± 0.4

### Molecular docking study

**Fig. 3:**
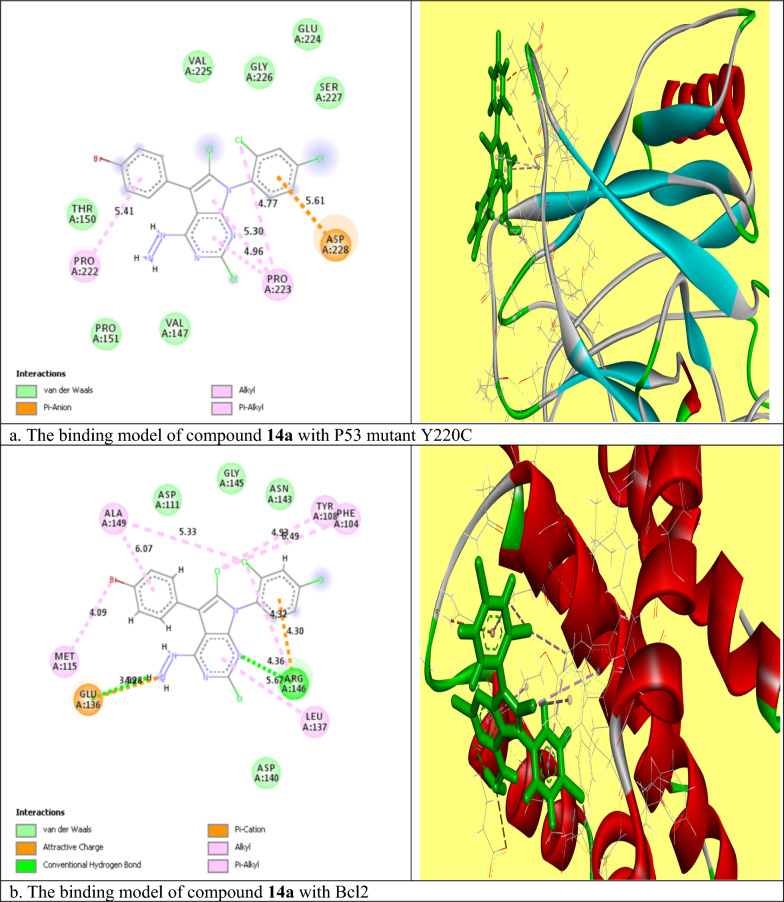

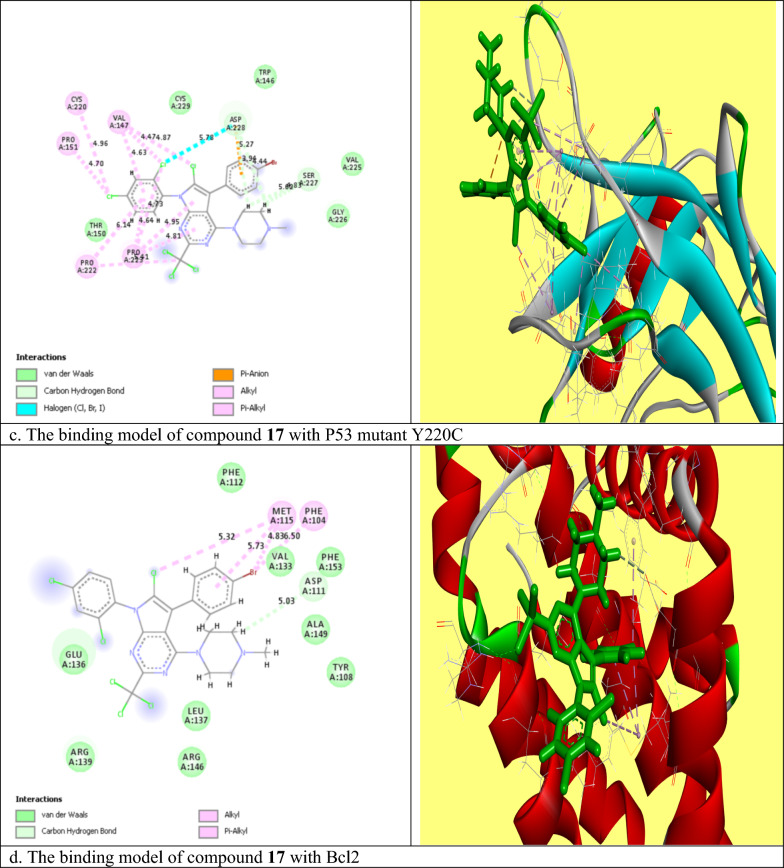
The molecular binding of compound **14a** with the active site of **a** P53 and **b** Bcl2, and compound **17** with the active site of **c** P53 and **d** Bcl2

Compounds **14a** and **17** were chosen for further molecular studies, as they had the most promising cytotoxic effect against MCF7 and PACA2 respectively. Herein, molecular docking studies were done to demonstrate the binding affinity of compounds **14a** and **17** toward P53 mutant Y220C and anti-apoptotic Bcl2. Root mean squared deviation (RMSD) for P53 mutant Y220C and Bcl2 (PDB ID: 5O1H and 6QGG respectively) were 0.7 and 2.5 respectively indicating the high accuracy of docking results. The binding affinities of compound **14a** toward P53 mutant Y220C and Bcl2 were − 14.98 and − 20.3 kcal/mole respectively which were very comparable to the standard value (− 15.82 and − 33.96 kcal/mole). Regarding compound **17**, the binding energies were − 16 and − 22.8 kcal/mole toward P53 mutant Y220C and Bcl2 respectively as compared to the standard (− 15.82 and − 33.9 kcal/mole). As shown in Fig. 3a, compound **14a** reactivated and stabilized p53 mutant Y220C through five interactions. One pi-anion hydrophobic interaction was seen between the benzene ring of dichlorobenzene moiety and ASP 228 with a bond distance of 5.61 A^0^. The remaining four interactions were pi-alkyl hydrophobic and the interacted amino acid residues were PRO 222 and PRO 223. It was noticed that compound **14a** inhibited Bcl2 via eleven interactions. These interactions included; a hydrogen bond between N of pyrimidine ring and ARG 146 with a bond distance of 4.36 A^0^, a hydrogen bond between H of NH_2_ moiety and GLU 136 (3.9 A^0^), attractive charge between the positive charge of N of NH_2_ moiety and GLU 136, pi-cation interaction between benzene ring of 2,4-dichloro benzene and ARG 146, and the remaining seven interactions were pi-alkyl and alkyl interactions with (PHE 104, TYR 108, MET 115, LEU 137, and ALA 149) amino acid residues (Fig. 3b). Compound **17** interacted with P53 mutant Y220C through 17 interactions (Fig. 3c). Four hydrogen bonds between hydrogen of 1,4diazinane and (SER 227 and ASP 228) with bond distances 5.62, 4.83, 3.94 and 4.44 A^0^ respectively, halogen interaction between Cl of dichlorobenzene moiety and ASP 228, and eleven alkyl and pi-alkyl hydrophobic interactions with VAL 147, PRO 151, CYS 220, PRO 222 and PRO 223 amino acid residues. At last, Fig. 3d showed the binding model of compound **17** with the active site of Bcl2 as followed: a carbon hydrogen bond between hydrogen of 1,4diazinane and ASP 111 (5.03A^0^), pi-alkyl hydrophobic interaction between Br of 4-bromo benzene ring and (MET 115 and PHE 104), pi-alkyl between benzene of 4-bromobenzene moiety and MET 115, and pi-alkyl between Cl of pyrimidine ring and MET 115. So, from the above results, we could assume the activating effect of compounds **14a** and **17** on P53 mutant Y220C and the inhibitory effect of compounds **14a** and **17** against Bcl2 anti-apoptotic protein and this assumption was confirmed in the subsequent Eliza assay section.

### Gene expression assay

#### Effects on mRNA expression of P53, BAX, BCL2 on MCF7

**Fig. 4:**
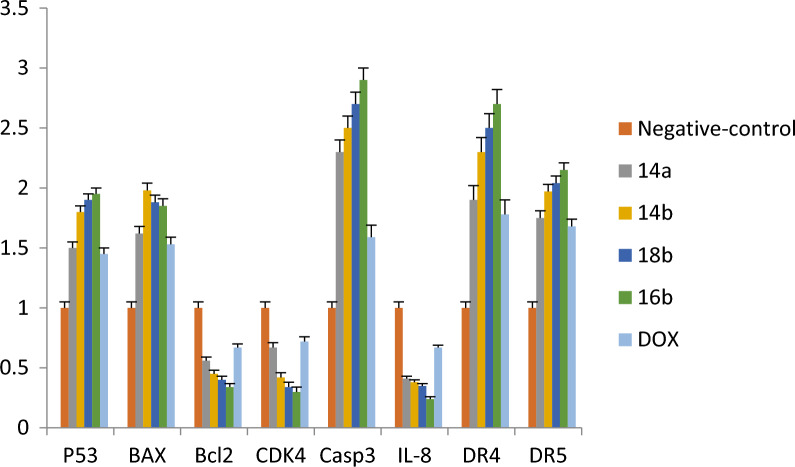
The RT-qPCR validation of mRNA expression for P53, BAX, BcL2, CDK4, caspase-3, Il-8, DR4 (TRAIL1), and DR5 (TRAIL2), in MCF7, Paca2 and A549 cells among groups of control, (Dox., negative control). Error bars represent the standard error of the mean (SEM). Means comparisons were performed by using. One-Way ANOVA test

The administration of **14a**, **14b**, **16b**, **18b** and Dox. up-regulated the mRNA levels of both P53 and BAX, whereas down-regulated Bcl-2 when compared with negative control. Moreover, we found that there are significant differences between all the compounds **14a**, **14b**, **16b**, **18b** and Dox. (Fig. 4). However, the injection of the newly synthesized compounds (**14a**, **14b**, **16b** and **18b**) and Dox. down-regulated mRNA level of Bcl-2 when compared with negative control, there were also significant differences between **14a**, **14b**, **16b**, **18b** and Dox. (Fig. 4).

#### Effects on mRNA expression of CDK4, Il-8, DR4 and DR5 on MCF7

The treated MCF7 cell line with **14a**, **14b**, **16b**, **18b** and Dox. exhibited increased DR4 and DR5 mRNA levels, but these treatments lowered CDK4 and IL-8 mRNA when compared with negative control. In addition, there were significant differences between the administration of the newly synthesized pyrrolo[2,3-*d*]pyrimidine derivatives and Dox. where **14a**, **14b**, **16b** and **18b** highly up-regulated DR4 and DR5 mRNA levels and down-regulated CDK4 and IL-8 mRNA levels compared with Dox. drug (Fig. 4).

It was known that Interleukin-8 (IL-8), is overexpressed in cancer cells compared with normal cells, and a high IL-8 level is correlated with a more aggressive tumor phenotype [53]. IL-8 has been shown to hunk TRAIL-induced cell death in ovarian cancer [54]. DR4 and DR5 (TRAILs receptors) are known to persuade apoptosis in an extensive variety of cancer cells, but rarely in normal cells. Normal cells are supposed to be resistant to TRAIL because their cell surface has the aptitude to express upper levels of TRAIL decoy receptors DcR1 and/or DcR2 [55, 56]. In addition, TRAIL death receptors are known to be transcriptionally up-regulated by p53.[57, 58] P53 has been shown to inhibit Bcl-2 activity and activated BAX and according to our data which revealed that compounds **14a**, **14b**, **16b** and **18b** significantly increased p53 mRNA levels, this could allow the upregulation of BAX, which in turn could inhibit IL-8 signaling and induce TRAIL death receptors expression.

#### Effects on mRNA expression of P53, BAX, BCL2 on Paca2

**Fig. 5:**
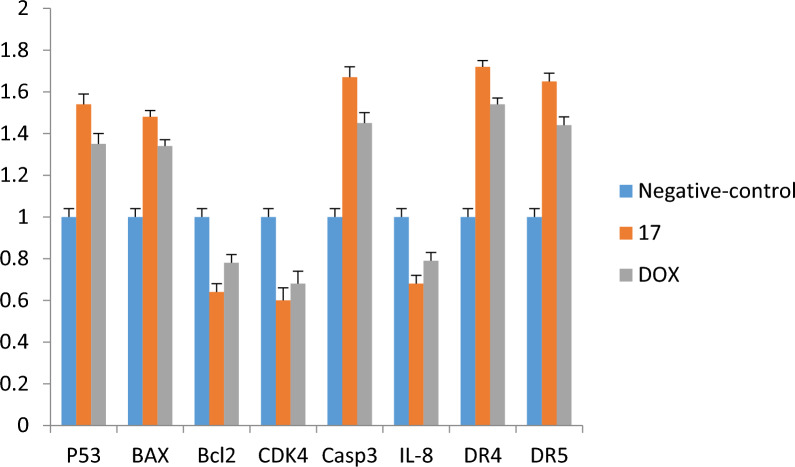
The RT-qPCR validation of mRNA expression for P53, BAX, BcL2, CDK4, caspase-3, Il-8, DR4 (TRAIL1), and DR5 (TRAIL2), in, Paca2 cells among groups of control, (Dox.; negative control). Error bars represent the standard error of the mean (SEM). Means comparisons were performed by using the One-Way ANOVA test

Compound **17** administration up-regulated the mRNA levels of both P53 and BAX in Paca2 cells, whereas it down-regulated Bcl-2 when compared to both Dox. and negative controls. Moreover, we found that there was no significant difference between negative control and Dox. (Fig. 5).

#### Effects on mRNA expression of CDK4, Casp-3, Il-8, DR4 and DR5 on Paca2

The treated Paca2 cell lines with **17** exhibited up-regulated Casp-3, DR4 and DR5 mRNA levels compared to both Dox. and negative control, while the treatment with **17** lowered CDK4 and IL-8 mRNA in comparison with both Dox. and negative control. In addition, there was no significant difference between administrations of Dox. and negative control (Fig. 5).

#### Effects on mRNA expression of P53, BAX, BCL2 on A549

**Fig. 6:**
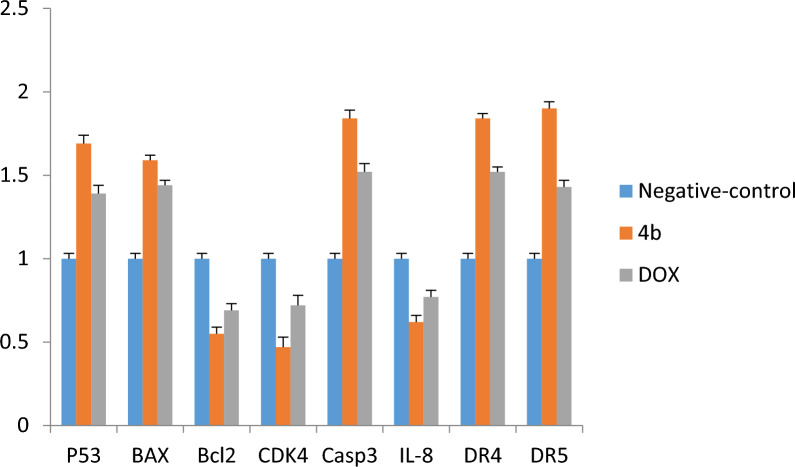
The RT-qPCR validation of mRNA expression for P53, BAX, BcL2, CDK4, caspase-3, Il-8, DR4 (TRAIL1), and DR5 (TRAIL2), in, A549 cells among groups of control, (Dox., negative control). Error bars represent the standard error of the mean (SEM). Means comparisons were performed by using the One-Way ANOVA test

The administration of **4b** and Dox. up-regulated the mRNA levels of both P53 and BAX in A549 cells, whereas down-regulated Bcl-2 when compared with negative control. In addition, we found that there were significant differences between **4b** and Dox. (Fig. 6). However, the injection of the **4b** and Dox. down-regulated mRNA level of Bcl-2 when compared with negative control and also there were significant differences between **4b** and Dox. (Fig. 6).

#### Effects on mRNA expression of CDK4, Casp-3, Il-8, DR4 and DR5 on A549

The treated lung cancer cells (A549) with 4b and the positive control (Dox.) showed up-regulated Casp-3, DR4 and DR5 mRNA levels. Moreover, it was observed considerable differences between the administration of **4b** and Dox., while **4b** is highly up-regulated of Casp-3, DR4 and DR5 mRNA levels and down-regulated CDK4 and IL-8 mRNA levels compared with the Dox. (Fig. 6).

Recently it was confirmed that the inhibition both of CDK4 and CDK6 genes influenced a varied range of cellular performances such as cancer cell metabolism and antitumor immunity. According to Shom et al. (2022) [59], Cyclin-dependent kinase (CDK4 and CDK6) are important mediators of cellular transference into the S phase and they are important growth and survival of many cancer types. According to our results, we found that both the newly synthesized pyrrolo[2,3-*d*]pyrimidine derivatives and Dox. were able to down-regulate CDK4 mRNA levels in treated cancer cells when compared with untreated cells (negative control) cells. In addition, the newly synthesized pyrrolo[2,3-*d*]pyrimidine derivatives were more potent in decreasing the level of mRNA of CDK4 than Dox. drug.

### Eliza analysis

**Fig. 7:**
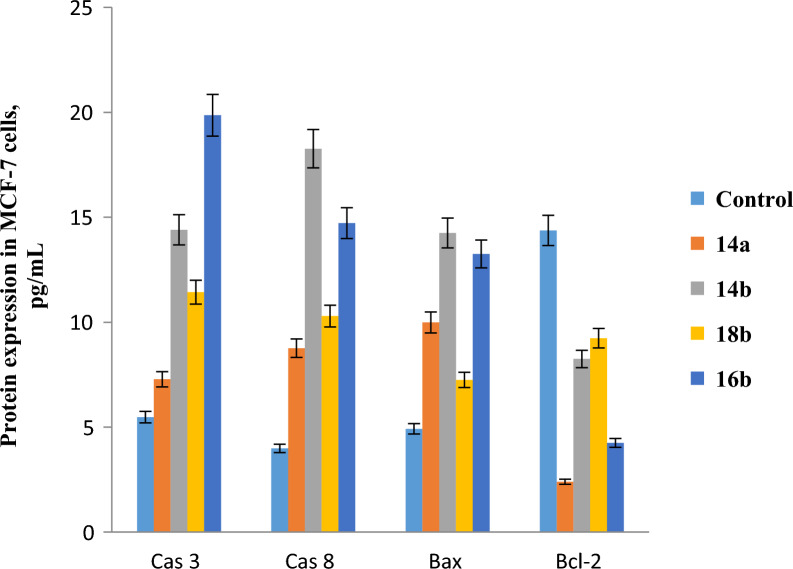
The protein expression level of caspase 3, caspase 8, BAX, and Bcl2 for **14a, 14b, 16b** and **18b** treated MCF7 cells. The untreated MCF7 cells were used as a negative control. Data demonstrated the mean ± SE

**Fig. 8:**
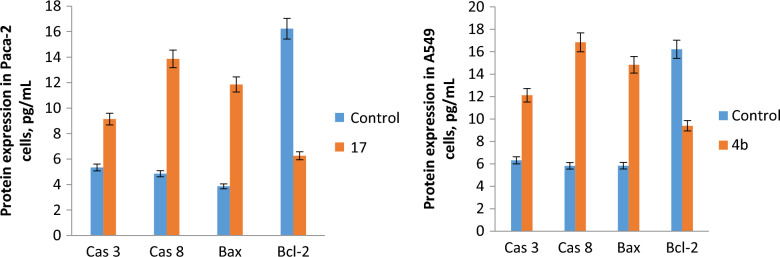
The protein expression level of caspase 3, caspase 8, BAX, and Bcl2 for **17** treated Paca2 cells (left) and **4b** treated A549 cells (right). The untreated cells were used as a negative control. Data represented mean ± SE

The activity of caspase 8, BAX, and Bcl2 in MCF7 cells after the treatment with the IC_50_ of **14a**, **14b**, **16b** and **18b** was assessed using the ELIZA assay (Fig. 7). Also, the activity of caspase 3, caspase 8, BAX, and Bcl2 was determined in **17-**treated Paca2 cells and **4b-**treated A549 cells (Fig. 8). Regarding MCF7 cells, it was found that both compounds **14b** and **16b** significantly increased the expression level of caspase 8 and BAX, (18.263 and 14.72 pg/ml for caspase 8, respectively) (14.25 and 13.25 pg/ml for BAX, respectively) relative to the control (3.99 and 4.92 pg/ml, respectively). **14a** and **18b** moderately enhanced the activity of caspase 8 and BAX, (8.76 and 10.29 pg/ml for caspase 8, respectively) (9.99 and 7.25 pg/ml for BAX, respectively). It was noticed that **14a** and **16b** greatly lowered the expression level of Bcl2 (2.4 and 4.25 pg/ml, respectively) relative to the control cells (14.37 pg/ml). The other two compounds **14b** and **18b** had a comparable effect on the activity of Bcl2 (8.25 and 9.24 pg/ml, respectively). As regard Paca2 cells, the activity of caspase 3, caspase 8 and BAX was significantly increased in response to **17** (9.14, 13.86 and 11.85 pg/ml, respectively) as compared with control (5.34, 4.85 and 3.86 pg/ml, respectively). While the concentration of Bcl2 was greatly decreased by **17** (6.26 pg/ml) compared to the control (16.23 pg/ml). In A549 cells, **4b** increased the expression level of caspase 3, caspase 8 and BAX (12.12, 16.84 and 14.83 pg/ml, respectively) relative to the control (6.32, 5.83 and 5.84 pg/ml, respectively). While the expression level of Bcl2 was decreased (9.4 pg/ml) compared to the control (16.21 pg/ml). Apoptosis is a programmed cell death and its regulation prevents many diseases including cancer. Caspases are a group of cysteine proteases that play a crucial role in apoptosis [60]. There are two pathways of apoptosis, extrinsic (death receptor) and intrinsic (mitochondrial) pathways [60]. In the extrinsic pathway, the activation of caspase 8 triggers the activation of executioner caspase 3 which leads to apoptosis [61]. While in the intrinsic pathway, caspase 8 activates Bid and the remaining reactions occur in mitochondria [62]. The mitochondrial pathway is regulated through Bcl2 family proteins, pro-apoptotic members such as BAX and anti-apoptotic members such as Bcl2 [62]. So, the up-regulation of caspase 3, caspase 8 and BAX and down-regulation of Bcl2 as indicated in the above results demonstrated the induction of apoptosis in MCF7, Paca2 and A549 treated cells.

### Flow cytometric analysis of cell cycle and apoptosis

**Fig. 9:**
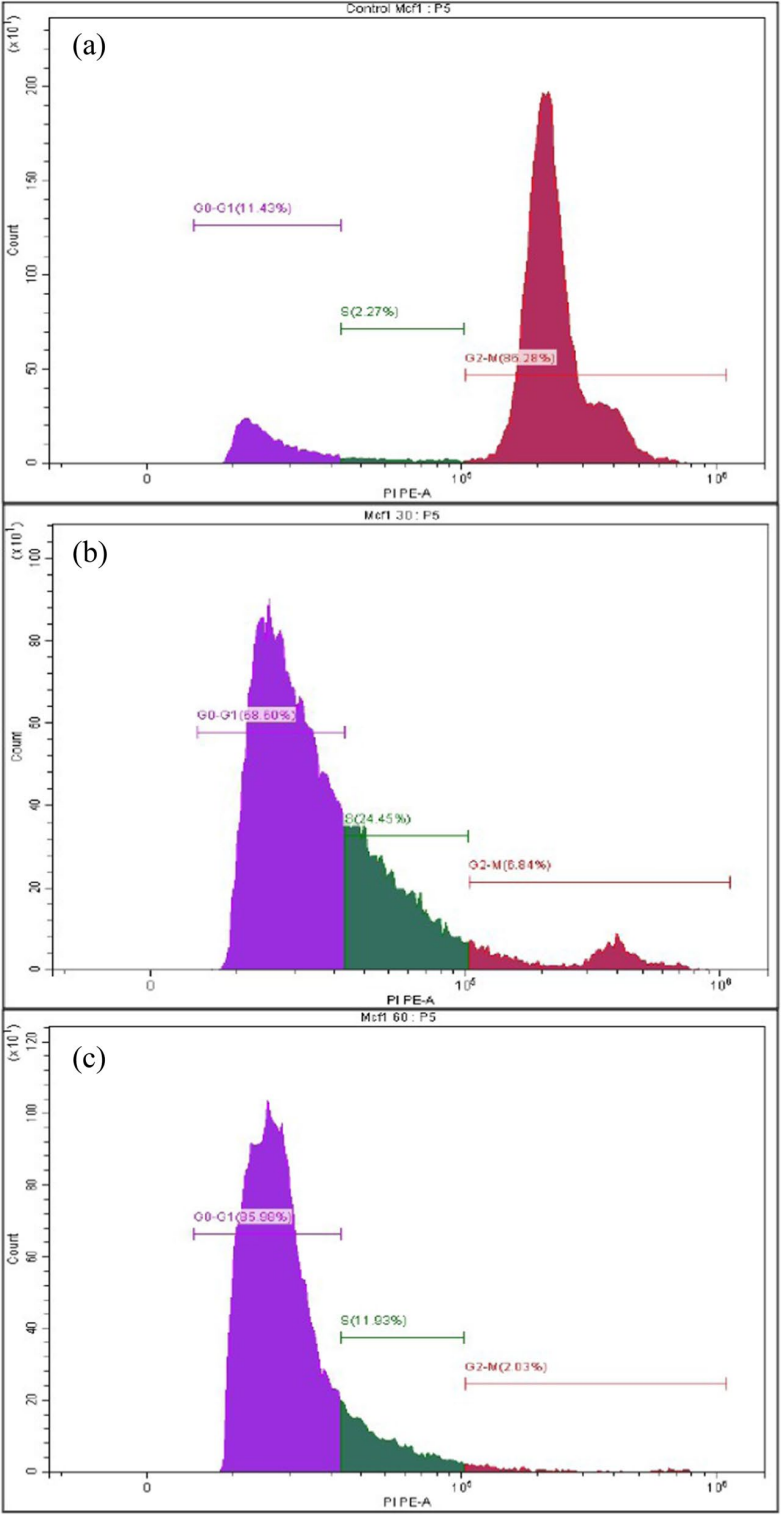
The cell distributions in the different phases of the cell cycle (G0/G1- S- G2/M) after 24 h of treatment with **a** untreated MCF7 cells, **b** **14a** treated MCF7 cells, **c** **14b** treated MCF7 cells

We examined the cell cycle distribution after 24-h treatment of MCF7 with IC_50_ concentrations of both the newly synthesized pyrrolopyrimidine compounds **14a** and **14b** to investigate the inhibitory effects on the proliferation of MCF7 cells. The untreated cells were used as a negative control for comparison purposes. As shown in Fig. 9, the percentage of cells in the G0/G1 phase was 11.43% for control cells, 68.60% for **14a** treated cells, and 85.98% for **14b** treated cells. It was noticed that the percentage of cells in the S phase was 1.91% for control cells, 17.61% for **14a** treated cells, and 7.34% for **14b** treated cells. While, the percentage of MCF7 cells in the G2/M phase was 72.58% in the control untreated cells, 4.93% in the **14a**-treated cells, and 1.937% in the **14b**-treated cells. So, compounds **14a** and **14b** caused cell cycle arrest at G1 and S phases as compared with the untreated control MCF7 cells. It was also notable that the percentage of treated arrested cells in the G1 phase was more than in the S phase and this coincided with our results in the gene expression section. As it was mentioned that both **14a** and **14b** down-regulated CDK4 which was responsible for G1/S phase progression.

**Fig. 10:**
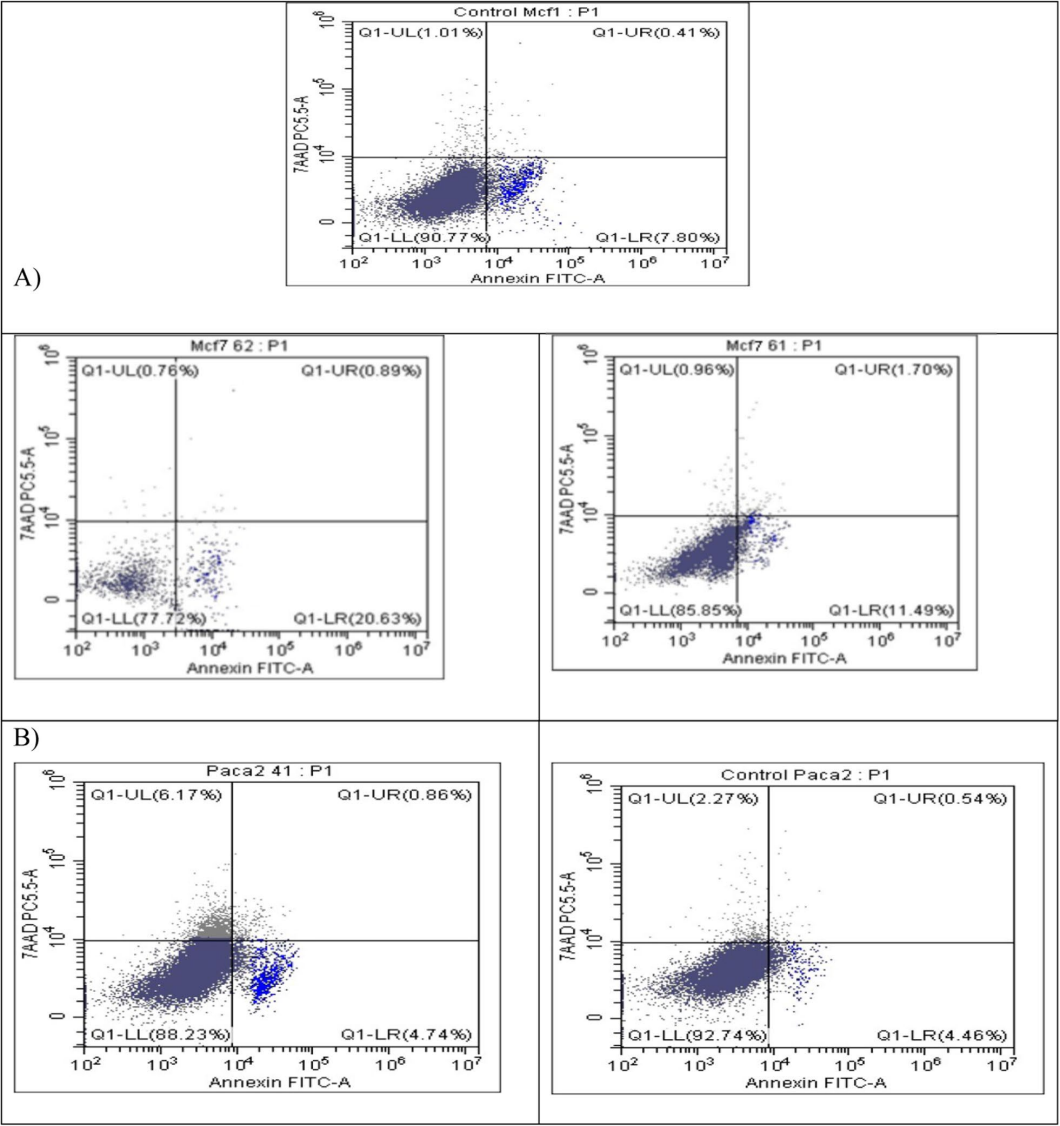
Flowcytometric analysis of apoptosis after 24 h of treatment for **A** MCF7 cells with IC_50_ of **16b** and **18b**; **B** Paca2 cells with IC_50_ of **17**. Untreated cells were used as a negative control

Additionally, the percentage of apoptotic and necrotic cells in treated MCF7 and Paca2 cells was determined using the Annexin V–FITC/PI Double Staining Kit. After 24 h of treatment of MCF7 with IC_50_ of **16b** and **18b**, it was found that the percentage of early apoptotic cells was increased to 11.49% and 20.63% respectively compared with untreated control cells (7.8%) (Fig. 10A). The percentage of late apoptotic cells was raised to 1.7% and 0.89% for **16b** and **18b** treated MCF7 cells respectively. While the percentage of necrotic cells was lowered to 0.96% and 0.76% respectively as compared with the control cells (1.01%). Regarding Paca2 cells, after 24 h of treatment with IC_50_ of **17**, it was found that the percentage of early and late apoptotic cells increased to 4.74% and 0.86% respectively. Also, the percentage of necrotic cells was raised to 6.17% compared with the control cells (2.27%) (Fig. 10B).

### DNA fragmentation

#### DNA fragmentation in Paca2

**Fig. 11:**
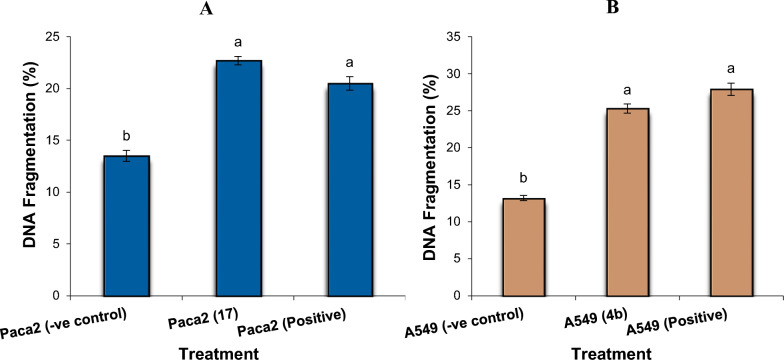
**A** DNA fragmentation detected in pancreatic cell lines (Paca2) treated with **17**. Means with different superscripts (^a, b^) between treatments in the same column are significantly different at *P* < 0.05. **B** DNA fragmentation detected in a lung cell line (A549) treated with **4b**. Means with different superscripts (^a, b^) between treatments in the same column are significantly different at *P* < 0.05

**Fig. 12:**
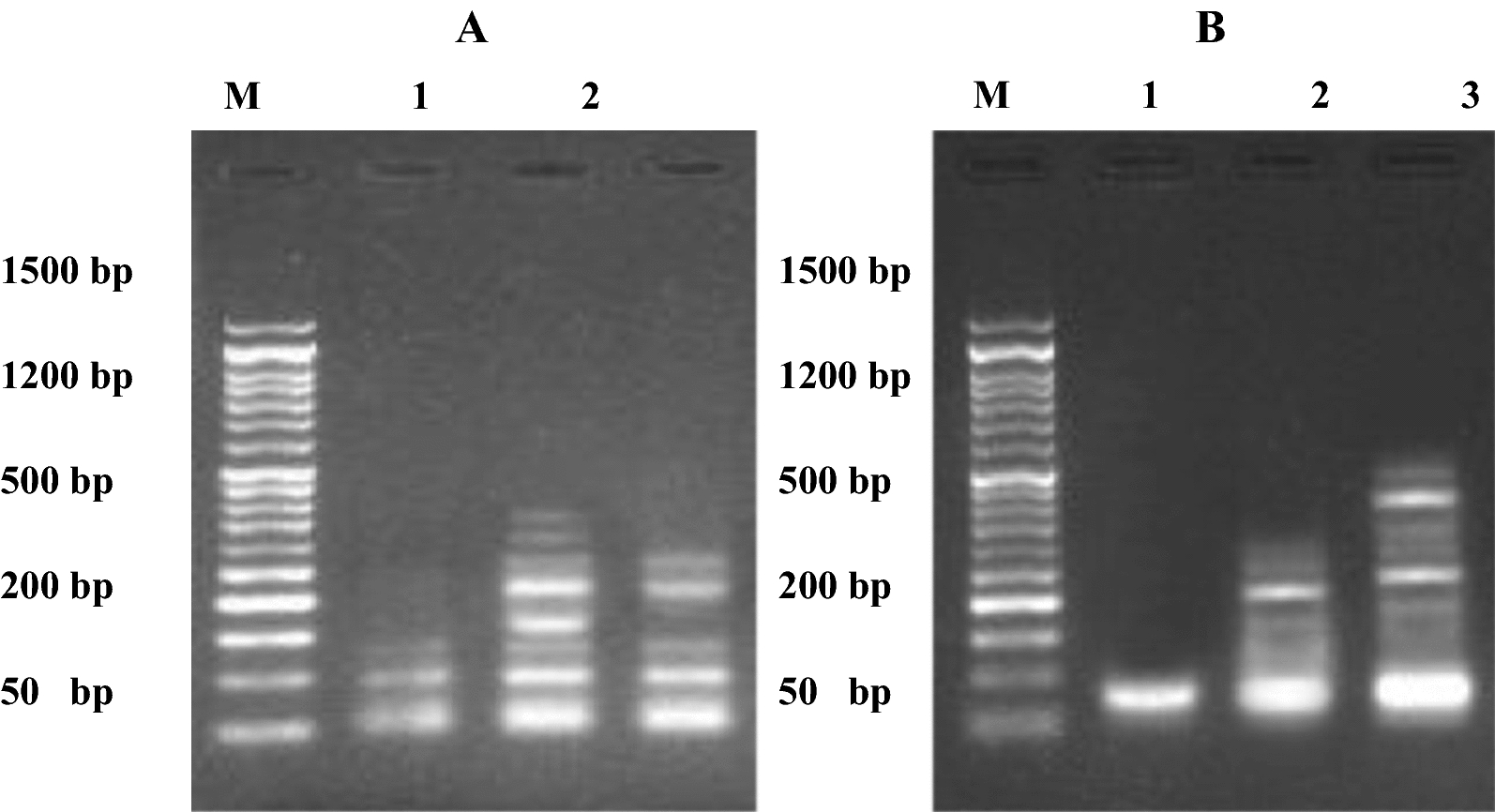
**A** DNA fragmentation detected with Agarose gel in pancreatic cell lines (Paca2) treated with **17**. M: represent DNA marker, Lane 1: represents negative cancer cell lines (-ve), Lane 2: represents Paca2 (**17**) and Lane 3: represents positive cancer cell lines (Dox.). **B** DNA fragmentation detected with Agarose gel in a lung cell line (A549) treated with **4b**. M: represent DNA marker, Lane 1: represents negative cancer cell lines (−ve), Lane 2: represents A549 (**4b**) and Lane 3: represents positive cancer cell lines (Dox.)

Determination of the rate of DNA fragmentation in the pancreatic cell line (Paca2) is depicted in Figs. 11A and 12A. The results showed that the negative samples of Paca2 exhibited a significant decrease (P < 0.01) in DNA fragmentation values compared with those in the treated samples (**17** and Dox. treated paca2 cells). However, the DNA fragmentation rates increased significantly (P < 0.01) in the treated Paca2 samples compared with the negative control. Moreover, the highest rate of DNA fragmentation was observed in Paca2-**17** more than that found in the Dox. treated cells.

#### DNA fragmentation in A549

Determination of the value of DNA fragmentation in the lung cell line (A549) is illustrated in Figs. 11B and 12B. The results revealed that the negative samples of A549 displayed a significant decrease (P < 0.01) in DNA fragmentation values compared with those in **4b** treated samples and positive cancer cell line. However, the DNA fragmentation value was increased significantly (P < 0.01) in the treated A549-**4b** cancer cell line sample compared with the negative control. Moreover, the highest value of DNA fragmentation was observed in the positive cancer cell line more than those in the A549-**4b** cell line.

#### DNA fragmentation in MCF7

**Fig. 13:**
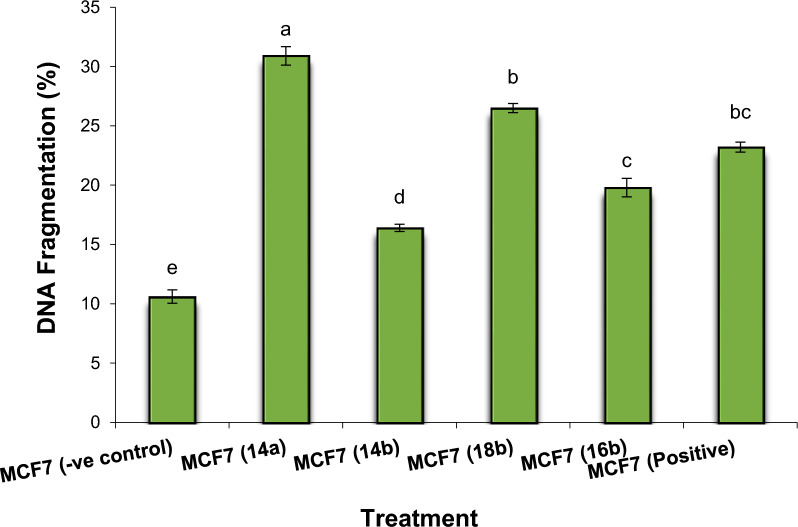
DNA fragmentation detected in breast cell line (MCF7) treated with **14a**, **14b**, **16b**, and **18b**. Means with different superscripts (^a, b, c, d, e^) between treatments in the same column are significantly different at *P* < 0.05

**Fig. 14:**
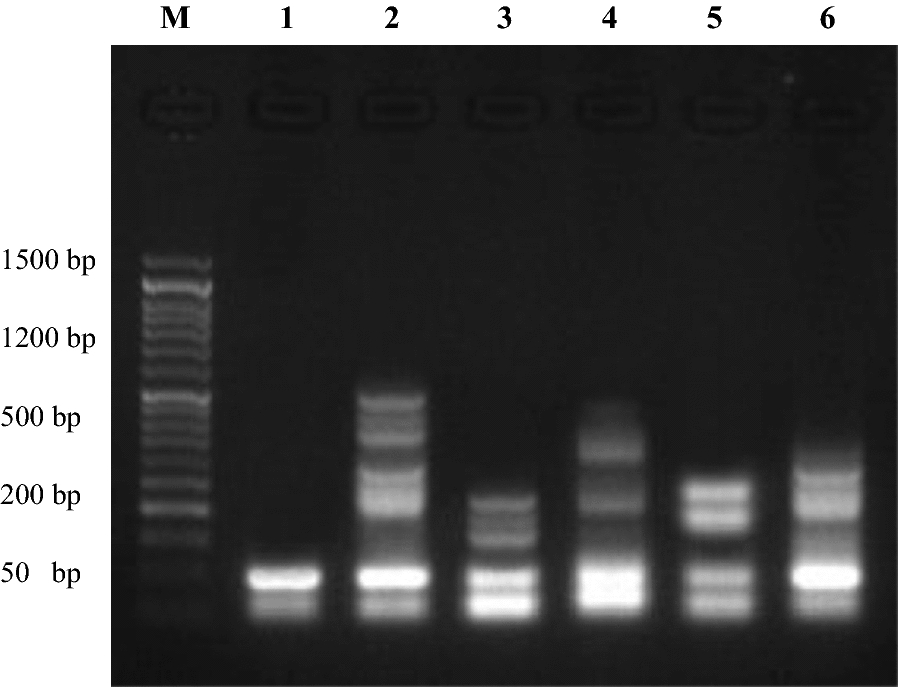
DNA fragmentation detected with Agarose gel in breast cell line (MCF7) treated with **14a**, **14b**, **16b**, and **18b**. M: represent DNA marker, Lane 1: represents negative cancer cell lines (-ve), Lane 2: represents MCF7 (**14a**), Lane 3: represents MCF7 (**14b**), Lane 4: represents MCF7 (**18b**), Lane 5: represents MCF7 (**16b**) and Lane 6: represents positive cancer cell lines (Dox.)

Assessment values of DNA fragmentation in MCF7 are summarized in Figs. 13 and 14. The results found that the negative samples of MCF7 showed a significant decrease (P < 0.01) in DNA fragmentation values compared with that observed in the treated samples (MCF7-**14a**, MCF7-**14b**, MCF7-**16b** and MCF7-**18b**) and positive cancer cell line. Conversely, the DNA fragmentation rates were found to be increased significantly (P < 0.01) in the treated MCF7 samples compared with the negative control. Moreover, the highest rate of DNA fragmentation was observed in MCF7-**14a** > MCF7-**18b** > MCF7- Positive > MCF7-**16b** > MCF7-**14b** cell line.

### Structure–activity relationship (SAR)

**Fig. 15:**
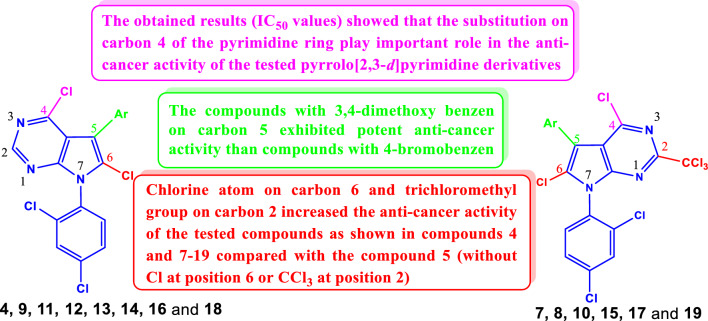
Structure–activity relationships (SAR) study of the prepared pyrrolo[2,3-*d*]pyrimidines **3**–**19**

The SAR study showed that the anti-cancer activity of the newly synthesized pyrrolo[2,3-*d*]pyrimidine derivatives changed due to the insertion of the chlorine atoms at positions 4 and 6 and the trichloromethyl group at position 2 of the pyrrolo[2,3-*d*]pyrimidine skeleton. As shown in Fig. 15, the pyrrolo[2,3-*d*]pyrimidine derivatives having 3,4-dimethoxy benzene (**4b**, **11b**, **14b**, **16b** and **18b**) on carbon 5 were found to be stronger anti-cancer agents compared with the related pyrrolo[2,3-*d*]pyrimidine derivatives containing 4-bromobenzene (**4a**, **11a**, **14a**, **16a** and **18a**). On the other hand, the chlorinated pyrrolo[2,3-*d*]pyrimidine derivatives with chlorine atoms in position 4 (or its substitutions) or in position 6, as well as the trichloromethyl group in position 2 (**4** and **7**–**19**), exhibited potent anti-cancer activity than the pyrrolo[2,3-*d*]pyrimidine without chlorine atom in position 2 as shown in compound **5**. Indeed, the presence of the trichloromethyl group at position 2 of the pyrrolo[2,3-*d*]pyrimidine core increased the anti-cancer properties of the prepared compounds compared to the related derivatives. Due to the IC_50_ values of the tested compounds, **8**, **10**, **15**, **17** and **19** exhibited potent anti-cancer activity on most of the tested cell lines compared with the related compounds **9**, **11**, **16** and **18**, respectively. The obtained results (IC_50_) showed that the substitution of carbon 4 of the pyrimidine ring plays an important role in the anti-cancer activity of the tested pyrrolo[2,3-*d*]pyrimidine derivatives. The presence of the hydrazide group on carbon 4 increased the activity as shown in compounds **14a**, **14b** and **15** on the most tested cell lines. Likewise, the *N*-methyl piperazine or morpholine groups on carbon 4 increased the anti-cancer activity of the compounds **16**–**19**. Finally, most of the prepared pyrrolo[2,3-*d*]pyrimidine derivatives **3**–**19** showed potent anti-cancer activates against the cell lines MCF7 and HePG2 than the other tested cell lines.

## Conclusion

In summary, we reported a new series of pyrrolo[2,3-*d*]pyrimidine derivatives using the microwave technique as an eco-friendly method. The prepared compounds were evaluated in vitro as anti-cancer agents against several human cancer cell lines. The presence of trichloromethyl group at position 2 and chlorine atom at position 6 of the pyrrolo[2,3-*d*]pyrimidine core increased the anti-cancer properties of the prepared compounds compared to the related derivatives. The molecular docking study confirmed the other experimental molecular studies. Where, most newly synthesized compounds might be considered as potent anticancer candidates for their ability to enhance the expression level of apoptotic genes (*P53, BAX, DR4, DR5*, and *caspase-3*), lower the expression of anti-apoptotic genes (*CDK4, Bcl2*, and *Il-8*), arrest the cell cycle at G1/S phase, induce apoptosis and cause DNA fragmentation in the selected cancer cell lines.

### Supplementary Information

**Additional file 1.** **Figure S1**: ^1^H- and ^13^C NMR spectra of **2a**. **Figure S2**: ^1^H- and ^13^C NMR spectra of **2b**. **Figure S3**: ^1^H- and ^13^C NMR spectra of **3a**. **Figure S4**: ^1^H- and ^13^C NMR spectra of **3b**. **Figure S5**: ^1^H- and ^13^C NMR spectra of **4a**. **Figure S6**: Mass spectroscopy of **4a**. **Figure S7**: ^1^H- and ^13^C NMR spectra of **4b**. **Figure S8**: ^1^H- and ^13^C NMR spectra of **5**. **Figure S9**: Mass spectroscopy of **5**. **Figure S10**: ^1^H NMR spectra of **6**. **Figure S11**: ^1^H- and ^13^C NMR spectra of **7**. **Figure S12**: ^1^H- and ^13^C NMR spectra of **8**. **Figure S13**: ^1^H- and ^13^C NMR spectra of **9a**. **Figure S14**: ^1^H- and ^13^C NMR spectra of **9b**. **Figure S15**: ^1^H-NMR spectrum of **10**. **Figure S16**: ^1^H- and ^13^C NMR spectra of **11a**. **Figure S17**: ^1^H- and ^13^C NMR spectra of **11b**. Mass spectrometry: of **11b**. **Figure S18**: ^1^H- and ^13^C NMR spectra of **12**. **Figure S19**: ^1^H- and ^13^C NMR spectra of **13**. **Figure S20**: ^1^H- and ^13^C NMR spectra of **14a**. **Figure S21**: ^1^H- and ^13^C NMR spectra of **14b**. **Figure S22**: ^1^H- and ^13^C NMR spectra of **15**. **Figure S23**: ^1^H- and ^13^C NMR spectra of **16a**. **Figure S24**: ^1^H- and ^13^C NMR spectra of **16b**. **Figure S25**: ^1^H- and ^13^C NMR spectra of **17**. **Figure S26**: ^1^H- and ^13^C NMR spectra of **18a**. **Figure S27**: ^1^H- and ^13^C NMR spectra of **18b**. Mass spectrometry: of **18b**. **Figure S28**: ^1^H- and ^13^C NMR spectra of **19**. Single-crystal X-ray report: of compound **4b**.

## Data Availability

Data supporting the productivity of this investigation are available from the corresponding author (Farid M Sroor) upon request.
