# Supplementary material for: Design, synthesis, in vitro anticancer, molecular docking and SAR studies of new series of pyrrolo[2,3-d]pyrimidine derivatives
Source: BMC Chem. 2023 Aug 28;17(1):106. doi: 10.1186/s13065-023-01014-0 (PMC10463376; doi:10.1186/s13065-023-01014-0)
Supplement: Supplementary file 1 — Additional file 1. Figure S1: 1H- and 13C NMR spectra of 2a. Figure S2: 1H- and 13C NMR spectra of 2b. Figure S3: 1H- and 13C NMR spectra of 3a. Figure S4: 1H- and 13C NMR spectra of 3b. Figure S5: 1H- and 13C NMR spectra of 4a. Figure S6: Mass spectroscopy of 4a. Figure S7: 1H- and 13C NMR spectra of 4b. Figure S8: 1H- and 13C NMR spectra of 5. Figure S9: Mass spectroscopy of 5. Figure S10: 1H NMR spectra of 6. Figure S11: 1H- and 13C NMR spectra of 7. Figure S12: 1H- and 13C NMR spectra of 8. Figure S13: 1H- and 13C NMR spectra of 9a. Figure S14: 1H- and 13C NMR spectra of 9b. Figure S15: 1H-NMR spectrum of 10. Figure S16: 1H- and 13C NMR spectra of 11a. Figure S17: 1H- and 13C NMR spectra of 11b. Mass spectrometry: of 11b. Figure S18: 1H- and 13C NMR spectra of 12. Figure S19: 1H- and 13C NMR spectra of 13. Figure S20: 1H- and 13C NMR spectra of 14a. Figure S21: 1H- and 13C NMR spectra of 14b. Figure S22: 1H- and 13C NMR spectra of 15. Figure S23: 1H- and 13C NMR spectra of 16a. Figure S24: 1H- and 13C NMR spectra of 16b. Figure S25: 1H- and 13C NMR spectra of 17. Figure S26: 1H- and 13C NMR spectra of 18a. Figure S27: 1H- and 13C NMR spectra of 18b. Mass spectrometry: of 18b. Figure S28: 1H- and 13C NMR spectra of 19. Single-crystal X-ray report: of compound 4b. [file 13065_2023_1014_MOESM1_ESM.pdf]

Supporting Information

Design, Synthesis, *In vitro* Anticancer and Molecular docking studies of New Series of Pyrrolo[2,3-d]pyrimidine Derivatives

Farid M. Sroor[1, *], Wael M. Tohamy[1], Khairy M. A. Zoheir[2], Karima F. Mahrous[2], Nagwan M. Abdelazeem[1], Nada S. Ibrahim[3]

1. Organometallic and Organometalloid Chemistry Department, National Research Centre, 12622 Cairo, Egypt
2. Cell Biology Department, National Research Centre, 12622-Dokki, Egypt
3. Department of Chemistry (Biochemistry Branch), Faculty of Science, Cairo University, Giza, Egypt

Farid M. Sroor✉

faridsroor@gmx.de,

fm.sroor@nrc.sci.eg

Contents

^{1}H and ^{13}C NMR spectra of **2a**.. S3

^{1}H and ^{13}C NMR spectra of **2b** S4

^{1}H and ^{13}C NMR spectra of **3a** S5

^{1}H and ^{13}C NMR spectra of **3b**.. S6

^{1}H and ^{13}C NMR spectra of **4a**.. S7

Mass spectrometry of **4a**.. S8

^{1}H and ^{13}C NMR spectra of **4b**.. S9

^{1}H and ^{13}C NMR spectra of **5**.. S10

Mass spectrometry of **5**.. S11

^{1}H and ^{13}C NMR spectra of **6**.. S12

^{1}H and ^{13}C NMR spectra of **7**.. S13

^{1}H and ^{13}C NMR spectra of **8**.. S14

^{1}H and ^{13}C NMR spectra of **9a**.. S15

^{1}H and ^{13}C NMR spectra of **9b**.. S16

^{1}H and ^{13}C NMR spectra of **10**.. S17

^{1}H and ^{13}C NMR spectra of **11a**.. S18

^{1}H and ^{13}C NMR spectra of **11b**.. S19

Mass spectrometry of **11b**.. S20

^{1}H and ^{13}C NMR spectra of **12**…………………………………………..………… S21

^{1}H and ^{13}C NMR spectra of **13**……………………………………….…...……… S22

^{1}H and ^{13}C NMR spectra of **14a**…………………………………...…………… S23

^{1}H and ^{13}C NMR spectra of **14b**…………………………………...…………… S24

^{1}H and ^{13}C NMR spectra of **15**……………………………………..…...……… S25

^{1}H and ^{13}C NMR spectra of **16a**……………………………….…...…………… S26

^{1}H and ^{13}C NMR spectra of **16b**……………………………….……...………… S27

^{1}H and ^{13}C NMR spectra of **17**…………………………………..……....……… S28

^{1}H and ^{13}C NMR spectra of **18a**…………………………………………………… S29

^{1}H and ^{13}C NMR spectra of **18b**…………………………………………………… S30

Mass spectrometry of **18b**…………………………………………………………….. S31

^{1}H and ^{13}C NMR spectra of **19**……………………………………….…..……… S32

X-ray report of **4b**……………….…………………………………….…………….... S33

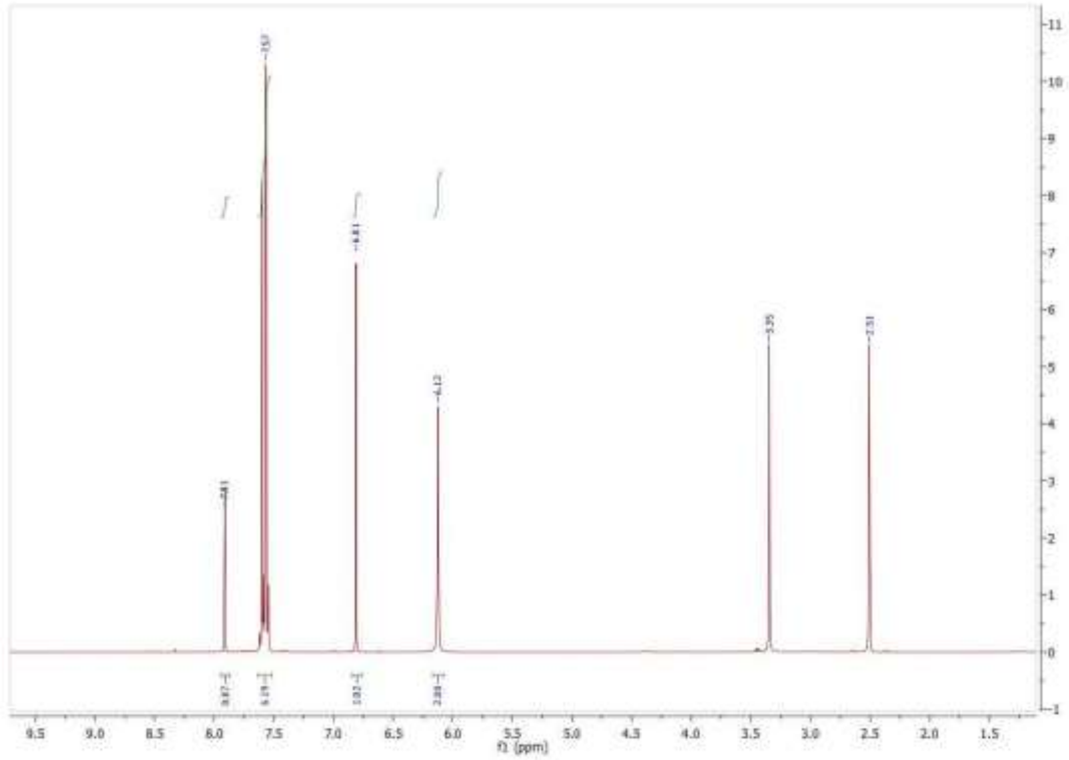

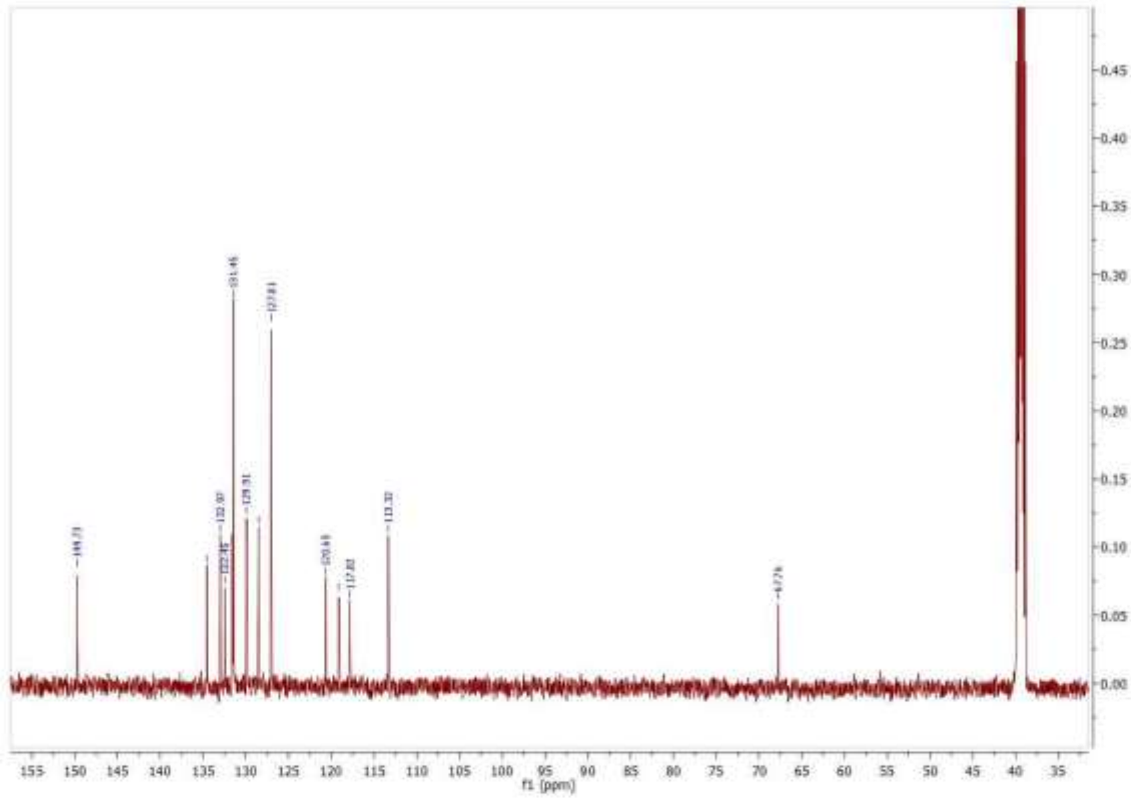

Figure S1. ^{1}H- and ^{13}C NMR spectra of **2a**.

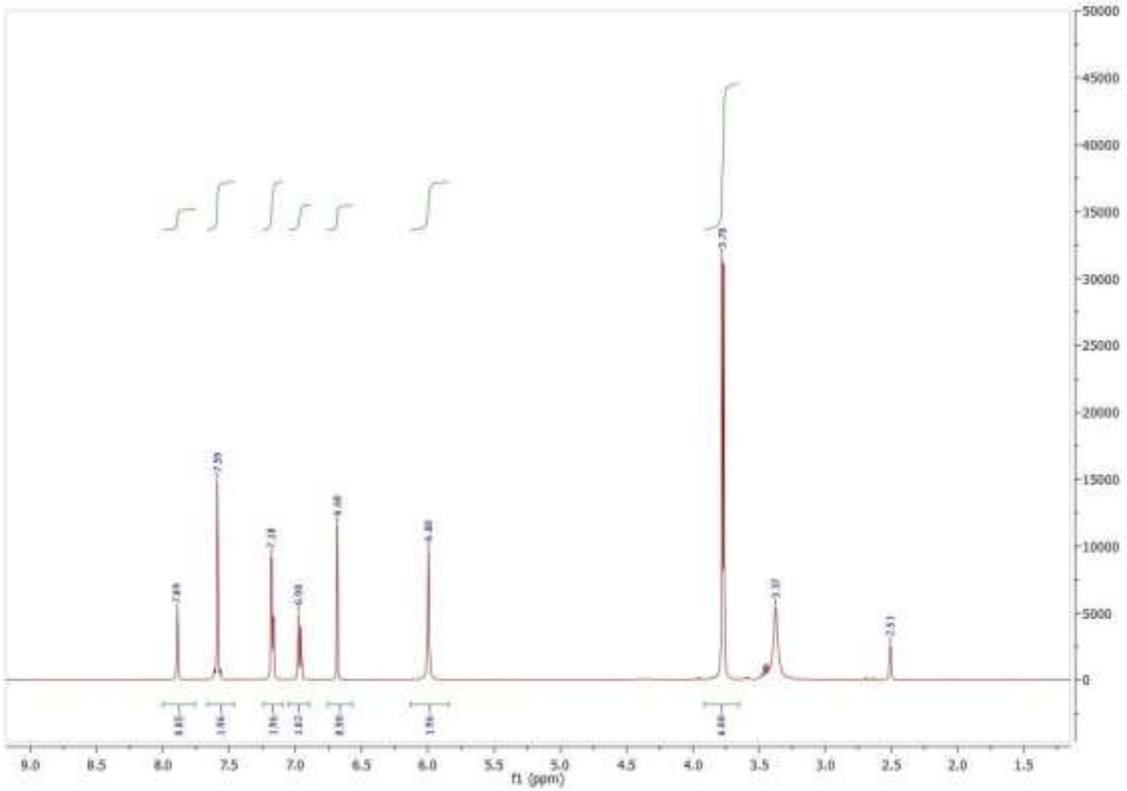

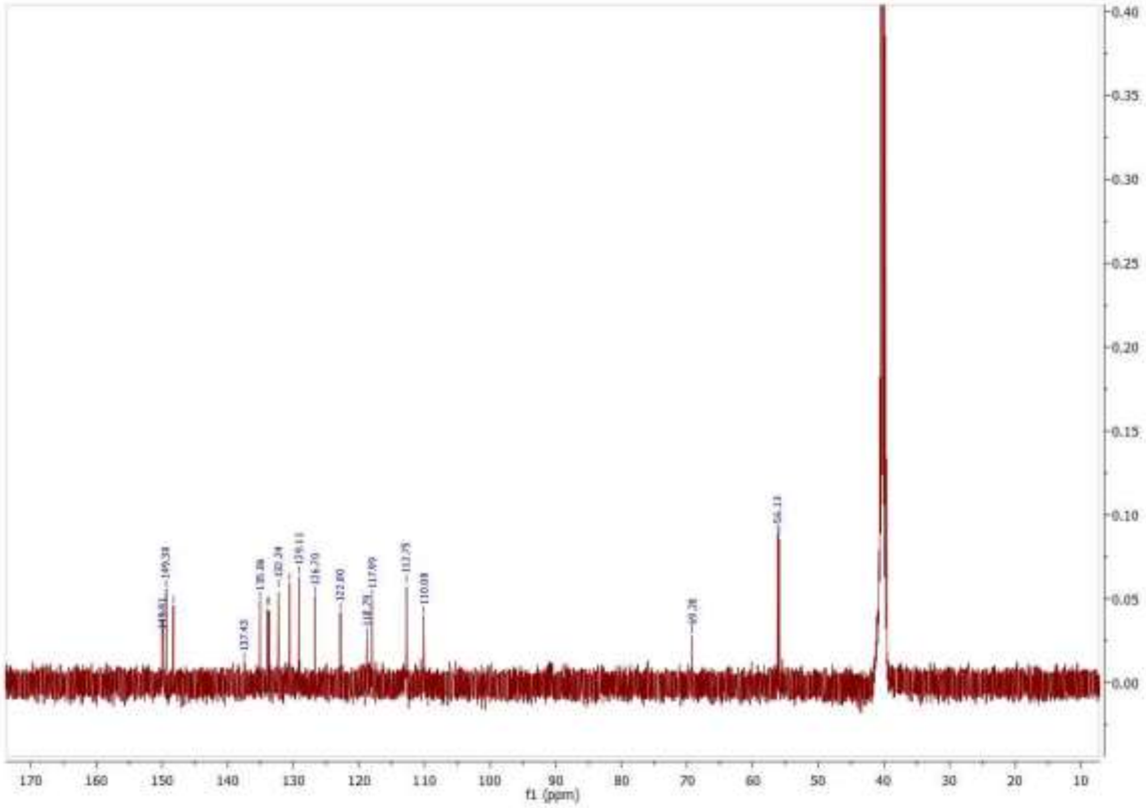

Figure S2. ^{1}H- and ^{13}C NMR spectra of **2b**.

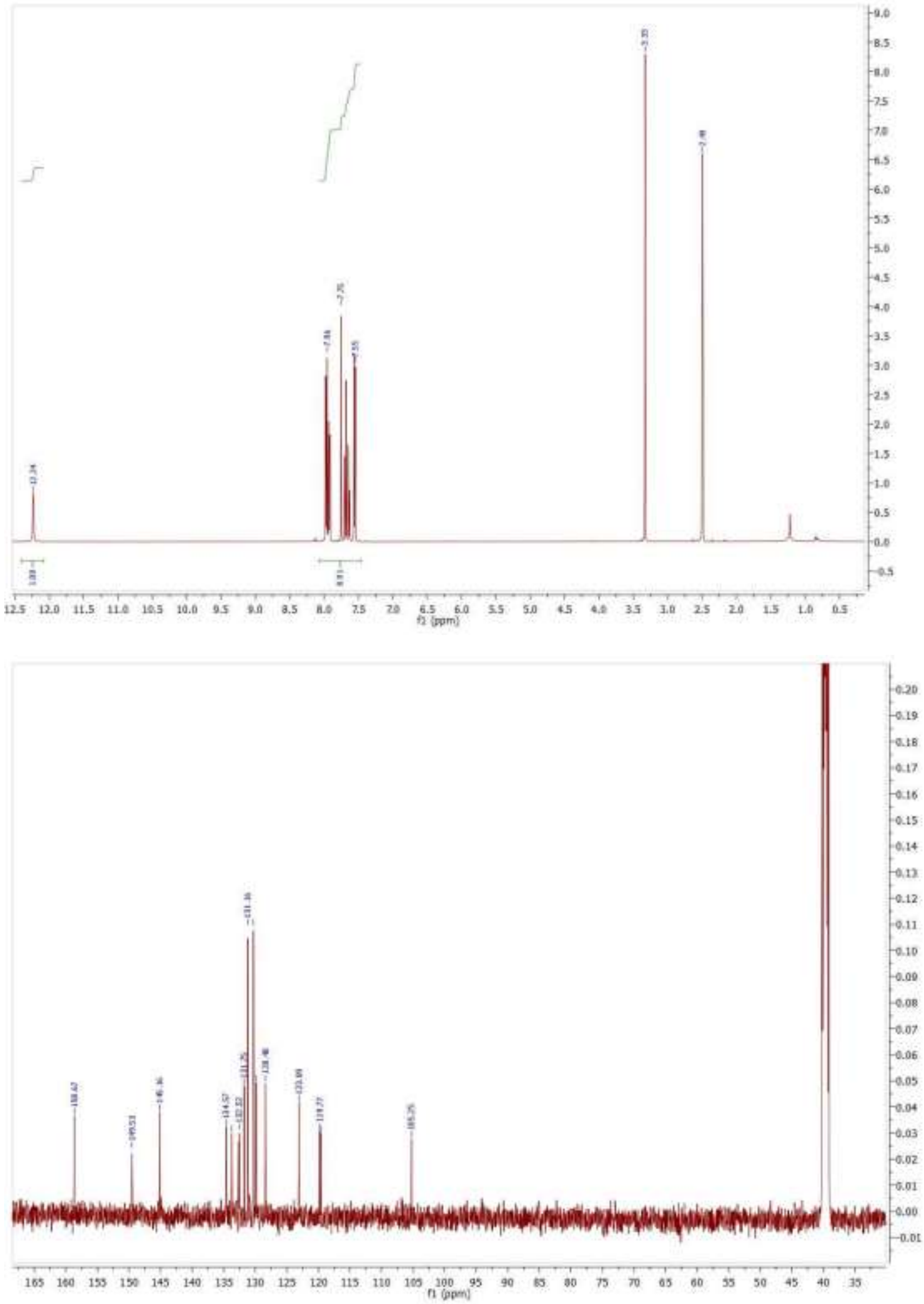

Figure S3. ^{1}H- and ^{13}C NMR spectra of **3a**.

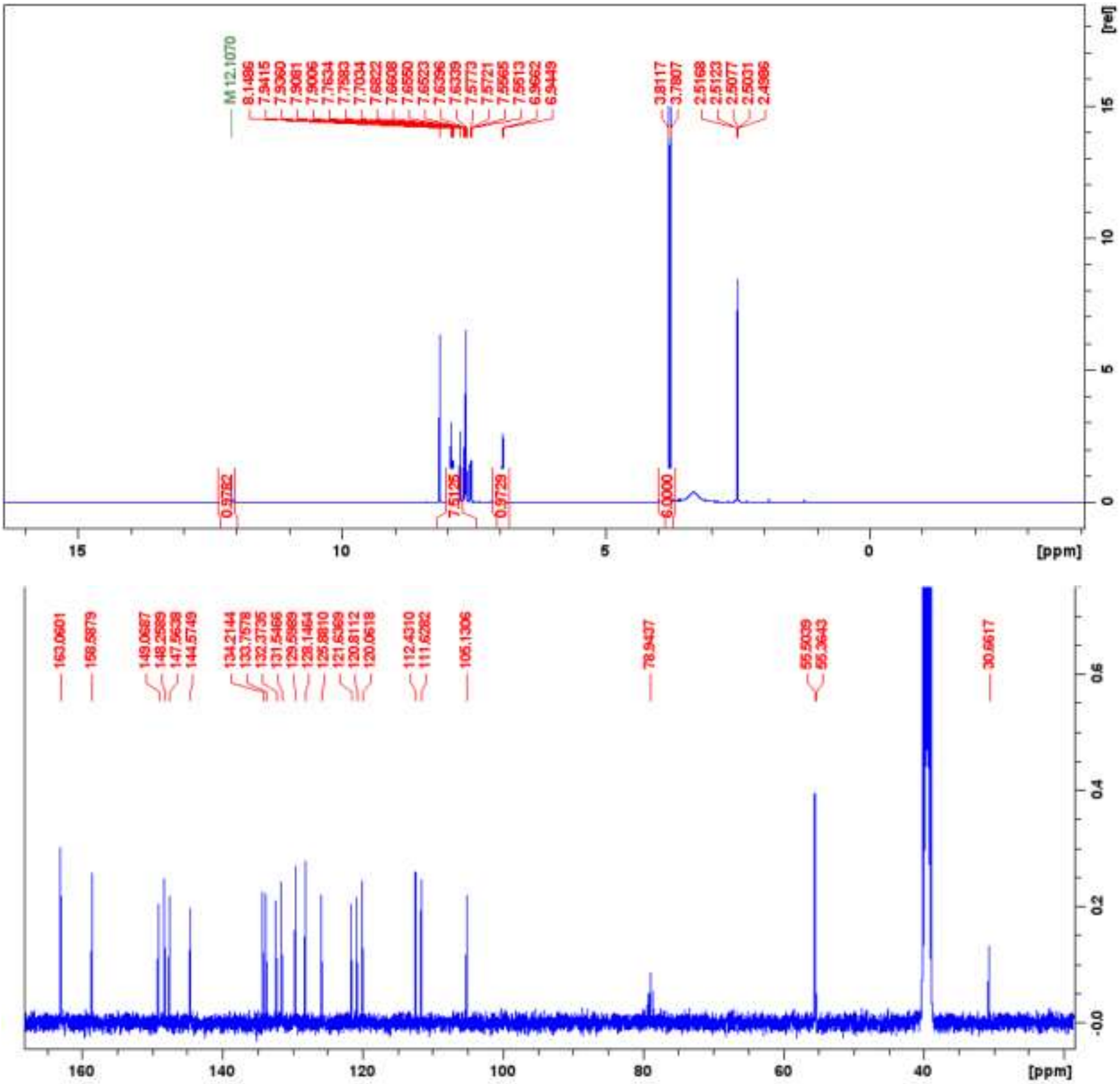

Figure S4. ^{1}H- and ^{13}C NMR spectra of **3b**.

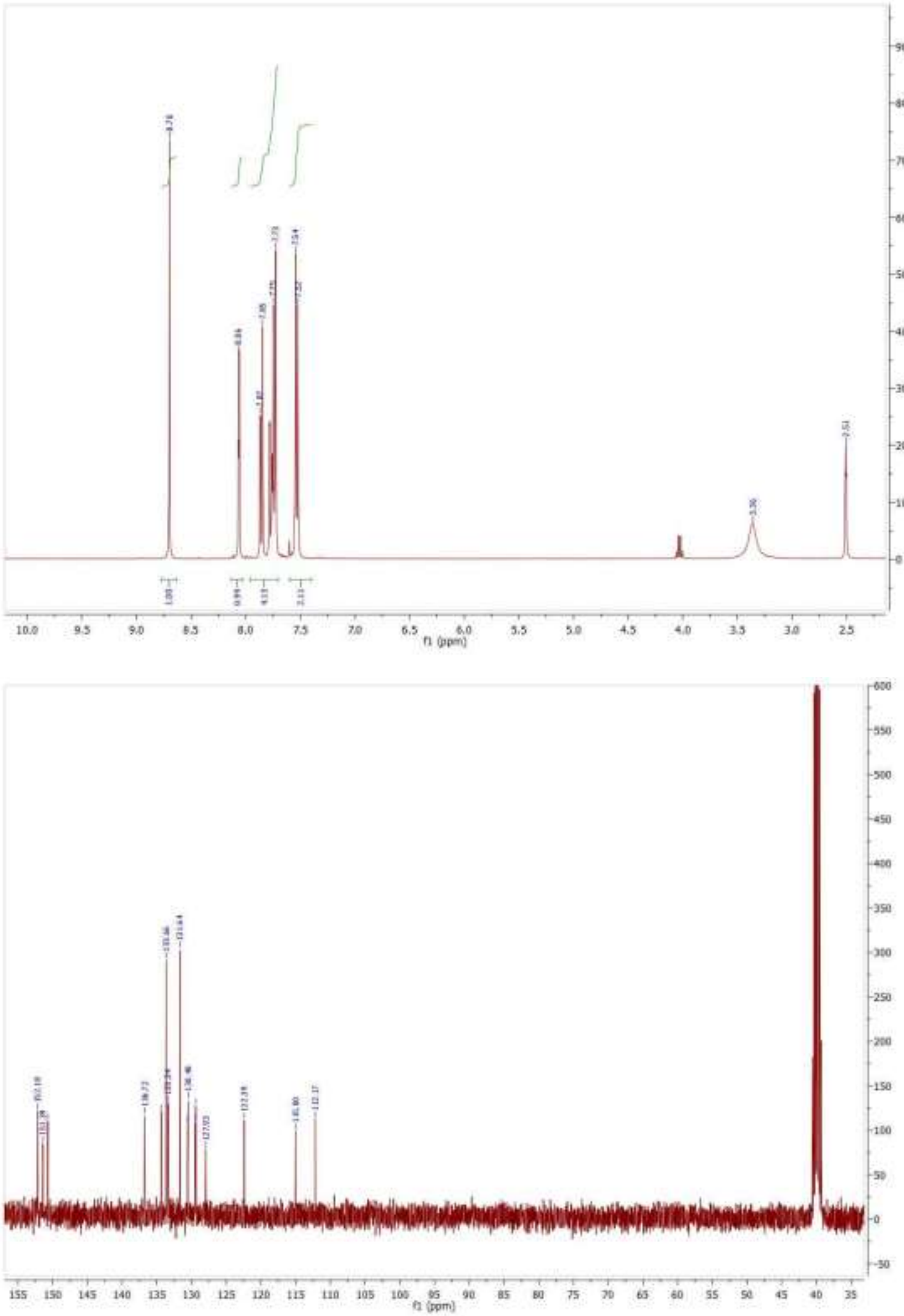

Figure S5. ^{1}H- and ^{13}C NMR spectra of **4a**.

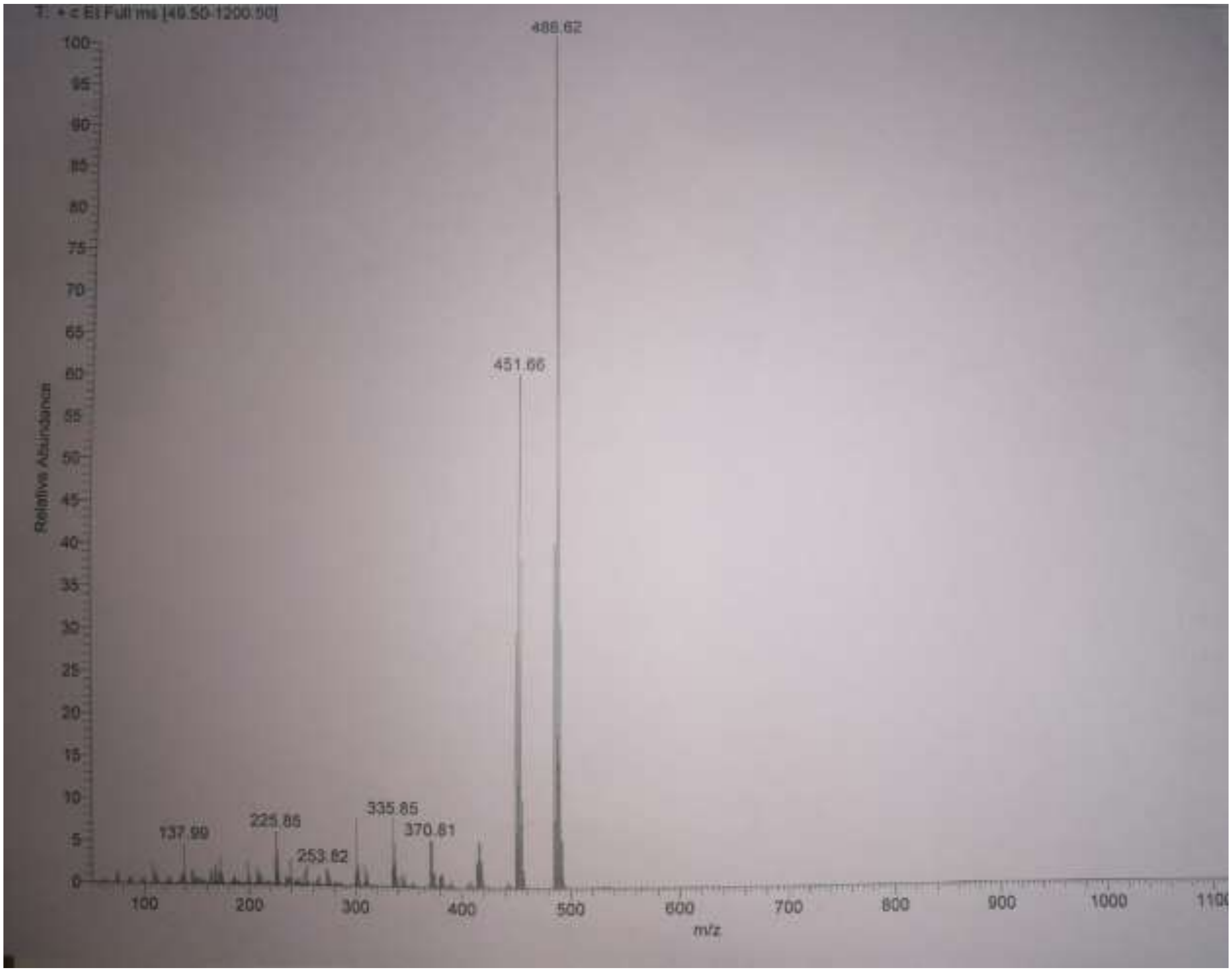

Figure S6. Mass spectroscopy of **4a**.

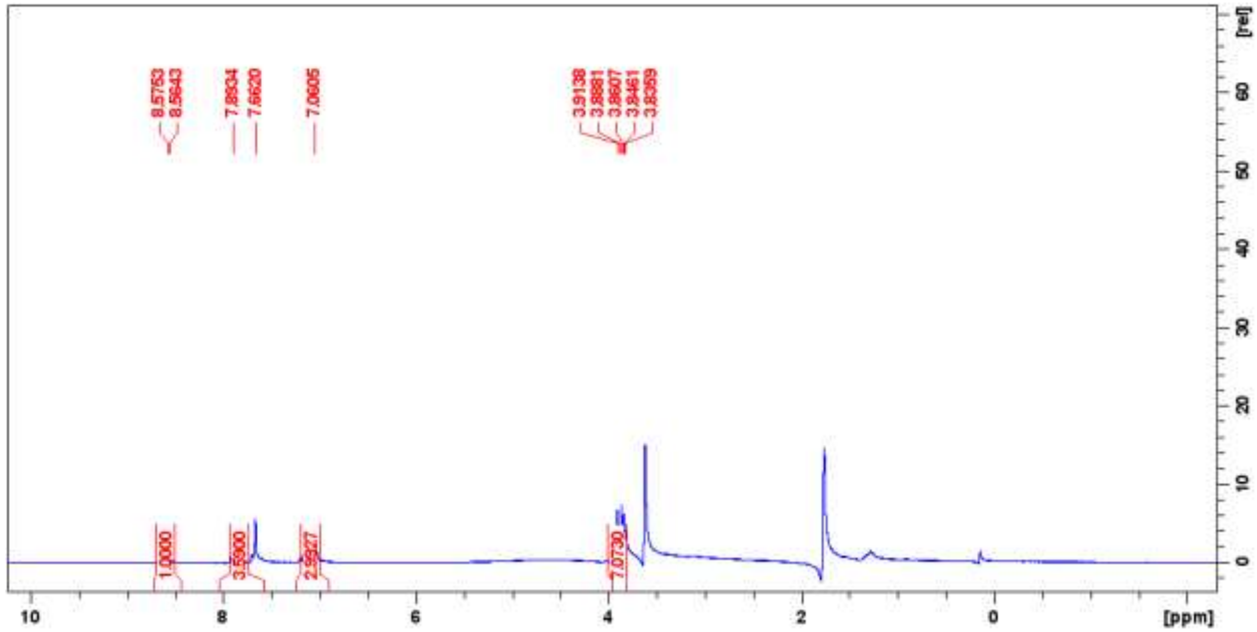

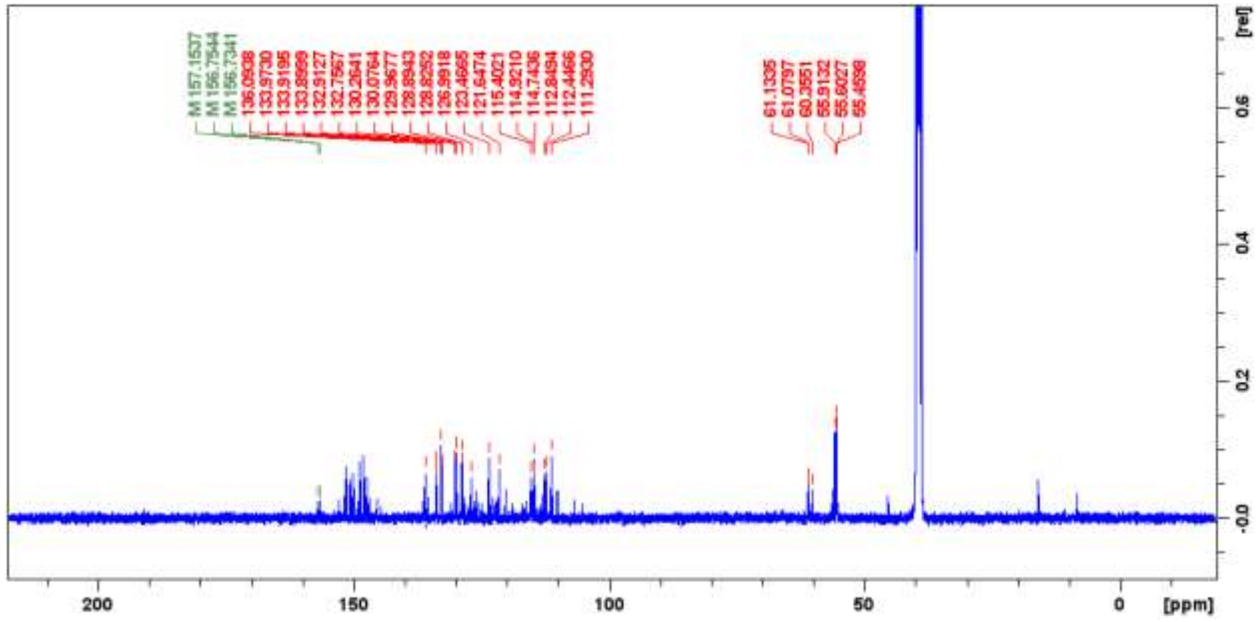

Figure S7. ¹H- and ¹³C NMR spectra of **4b.**

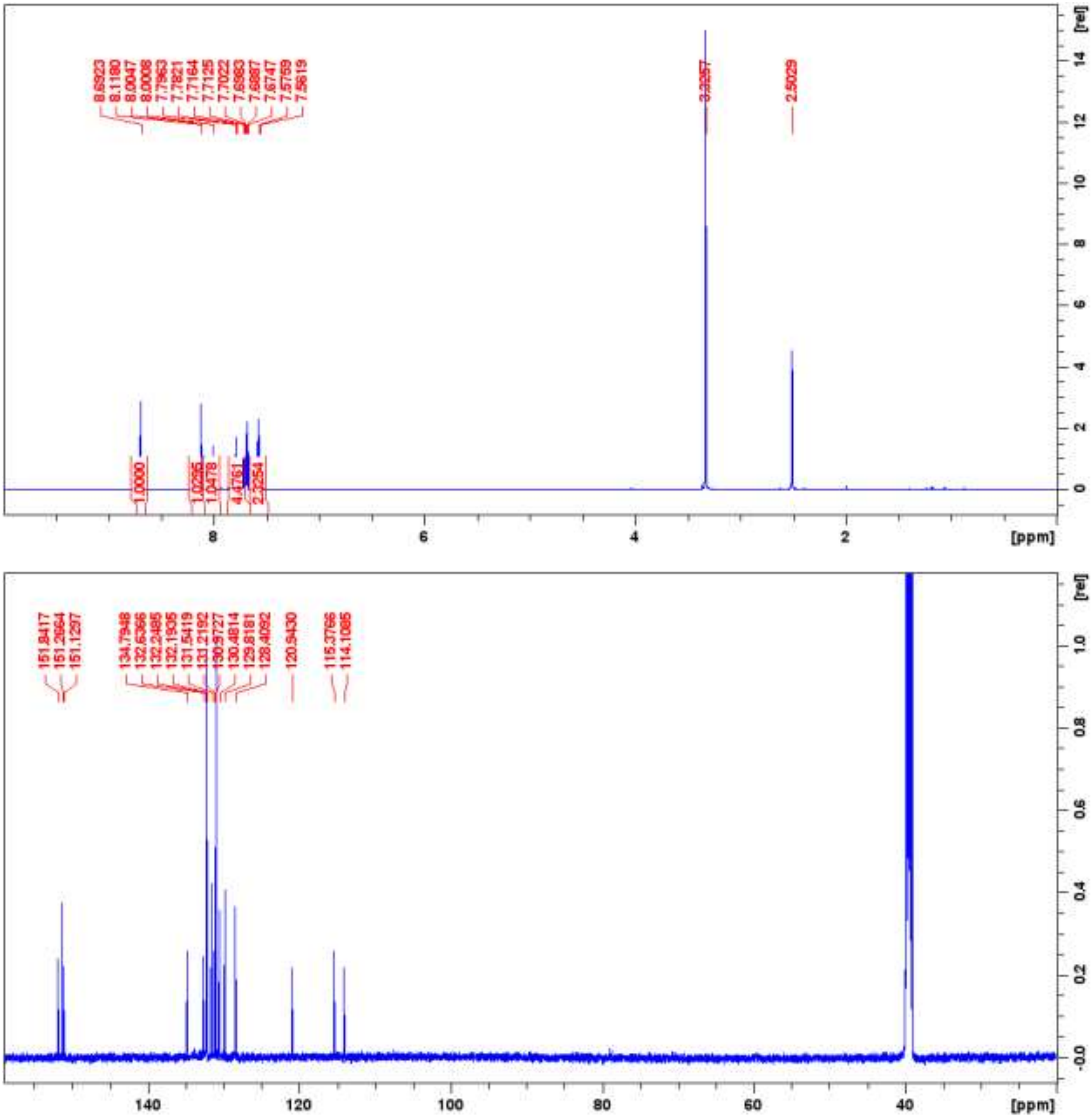

Figure S8. ^{1}H- and ^{13}C NMR spectra of **5.**

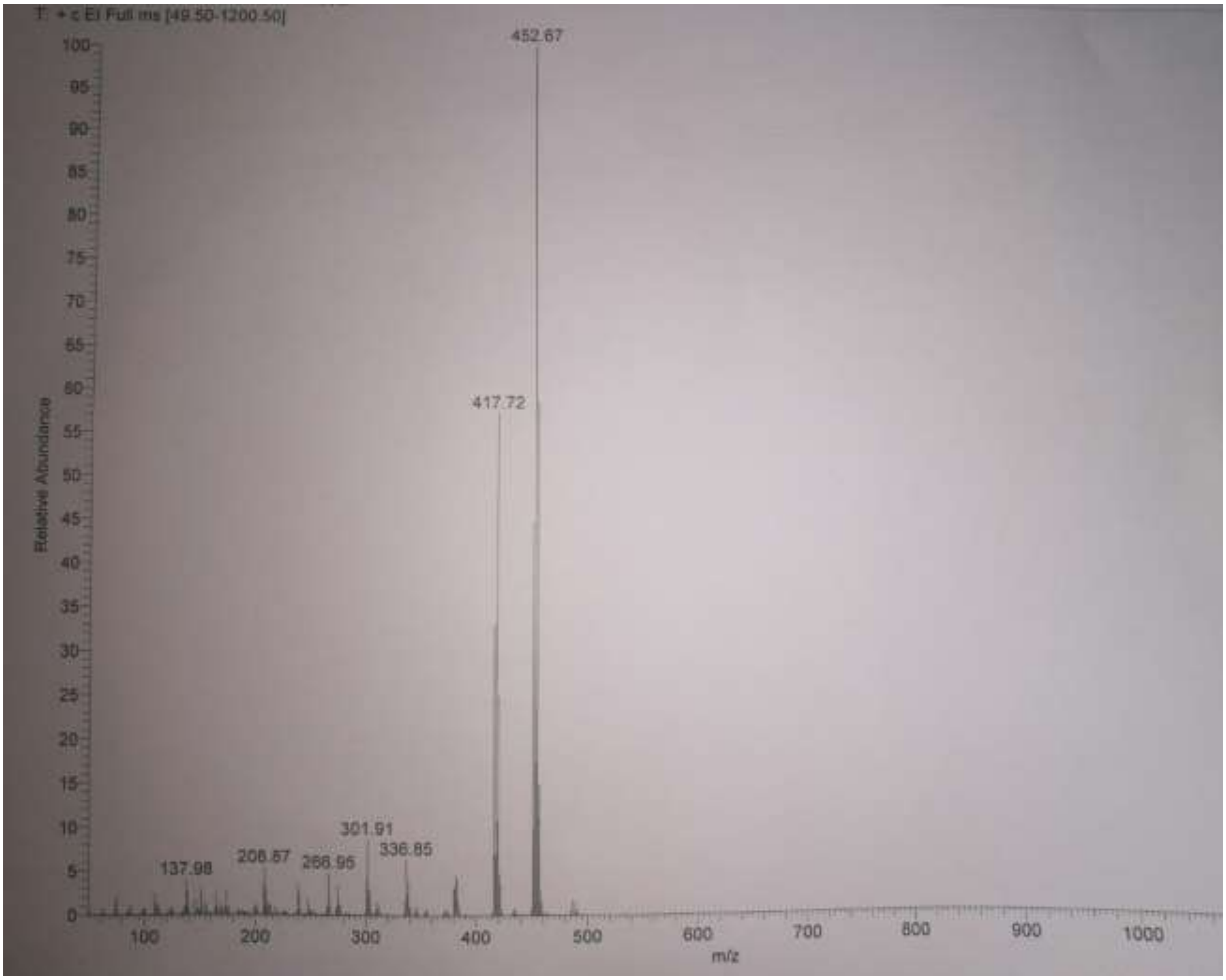

Figure S9. Mass spectroscopy of **5**.

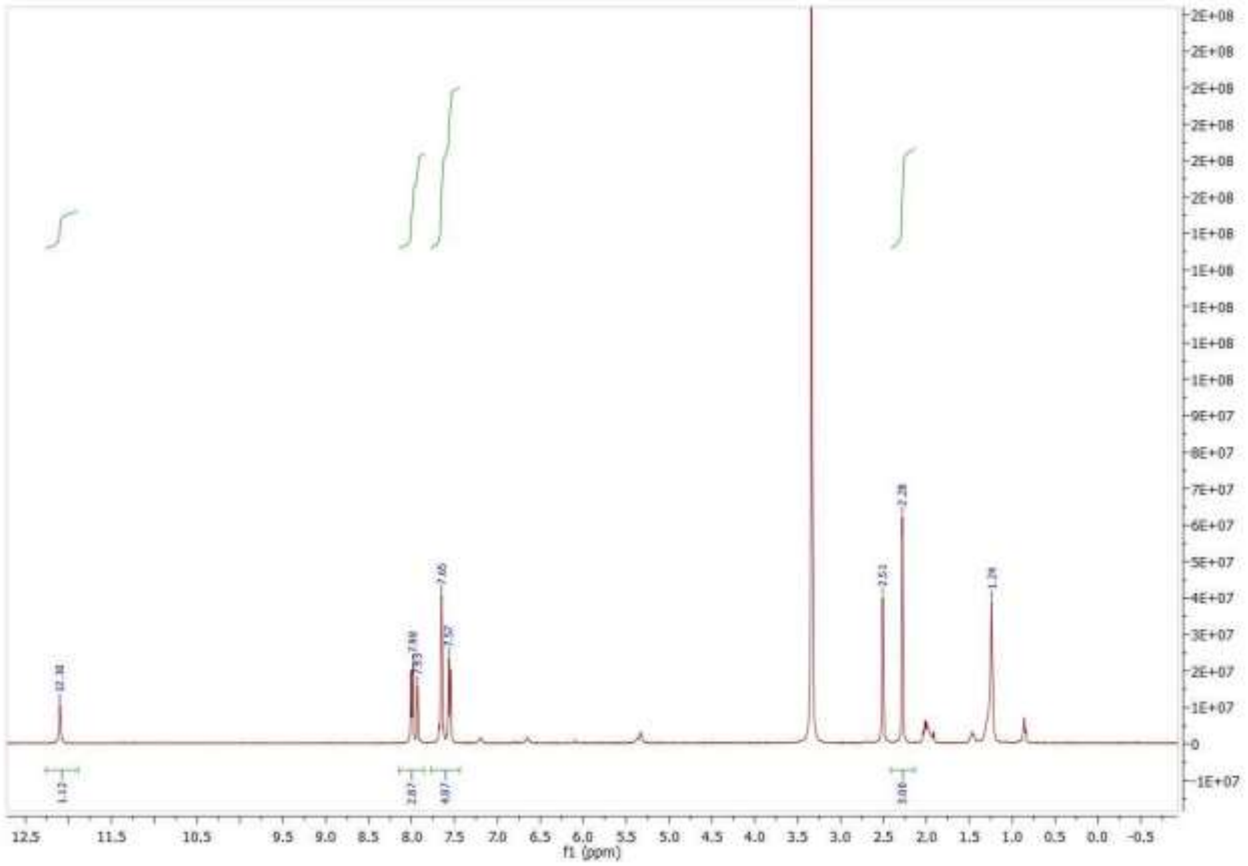

Figure S10. ^{1}H NMR spectra of **6**.

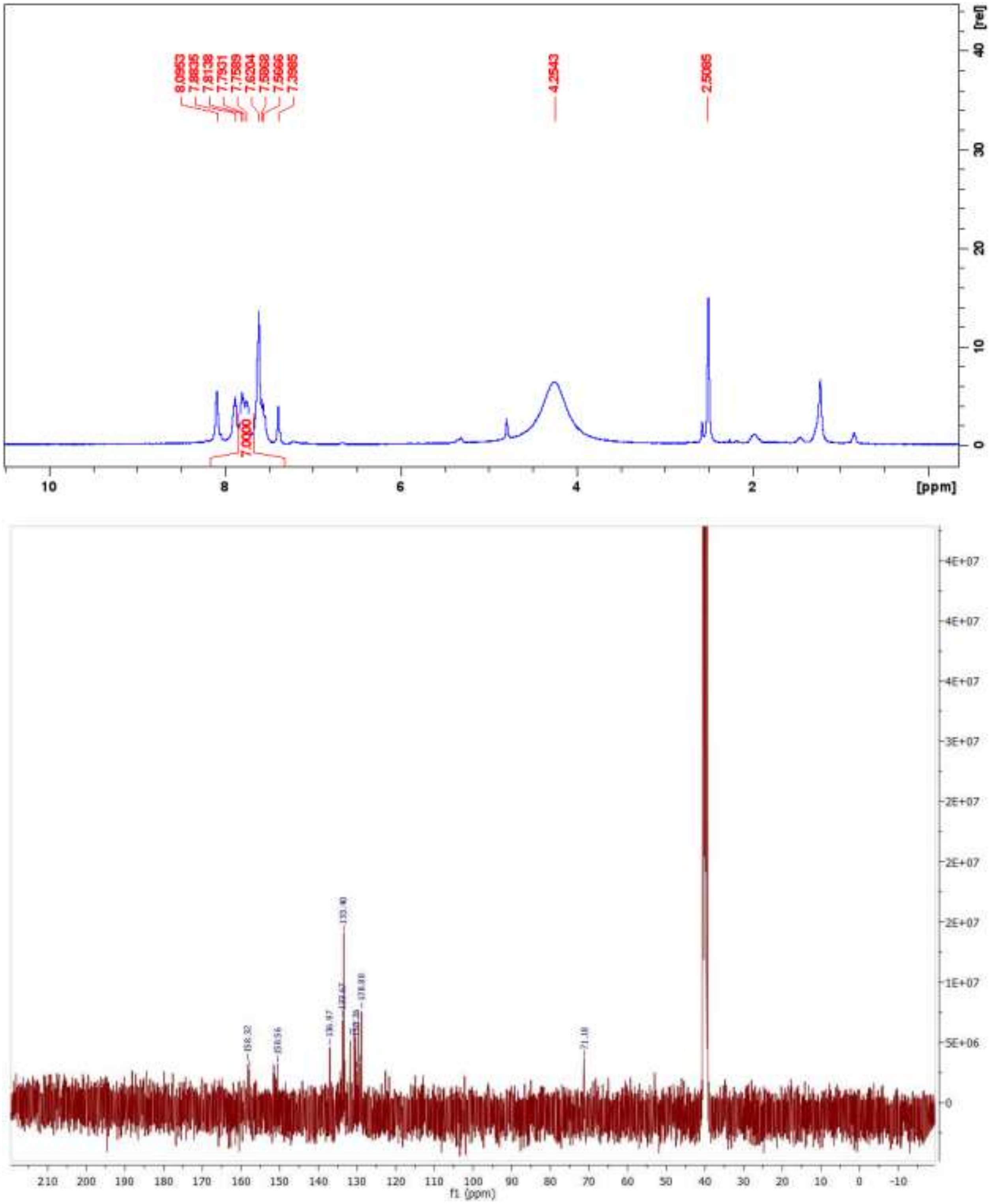

Figure S11. ^{1}H- and ^{13}C NMR spectra of **7**.

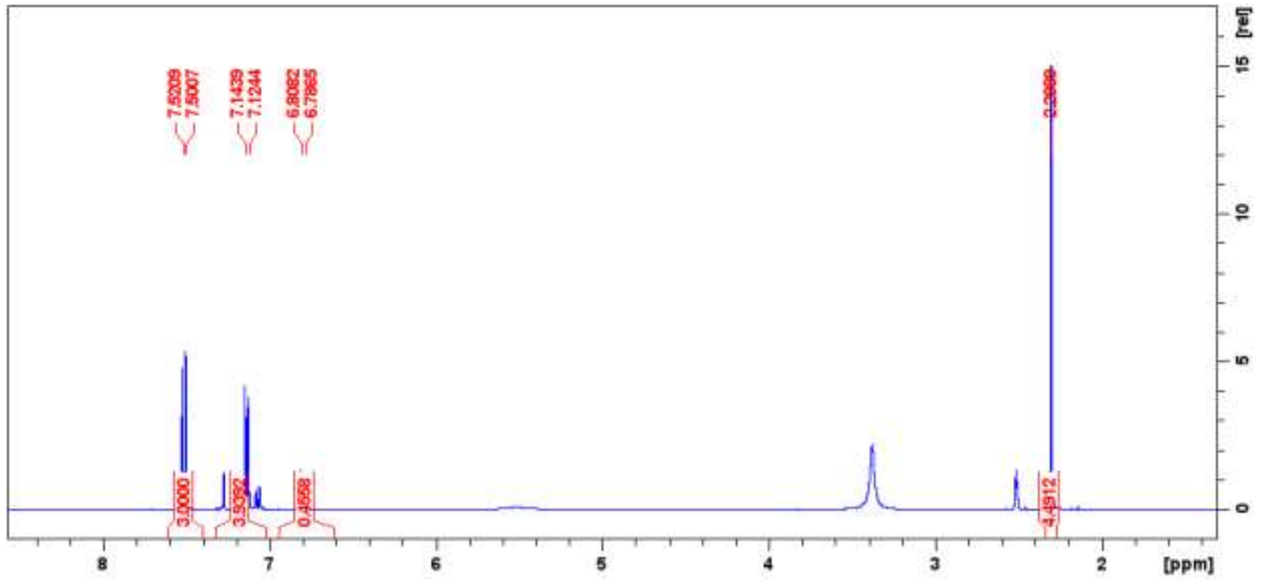

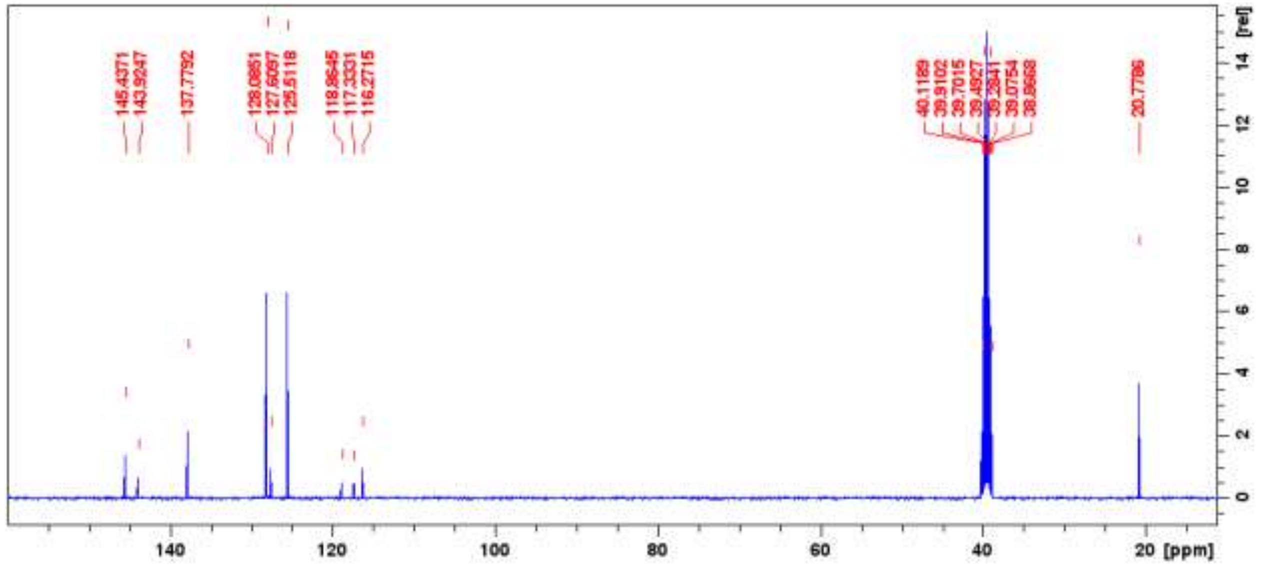

Figure S12. ^{1}H- and ^{13}C NMR spectra of **8**.

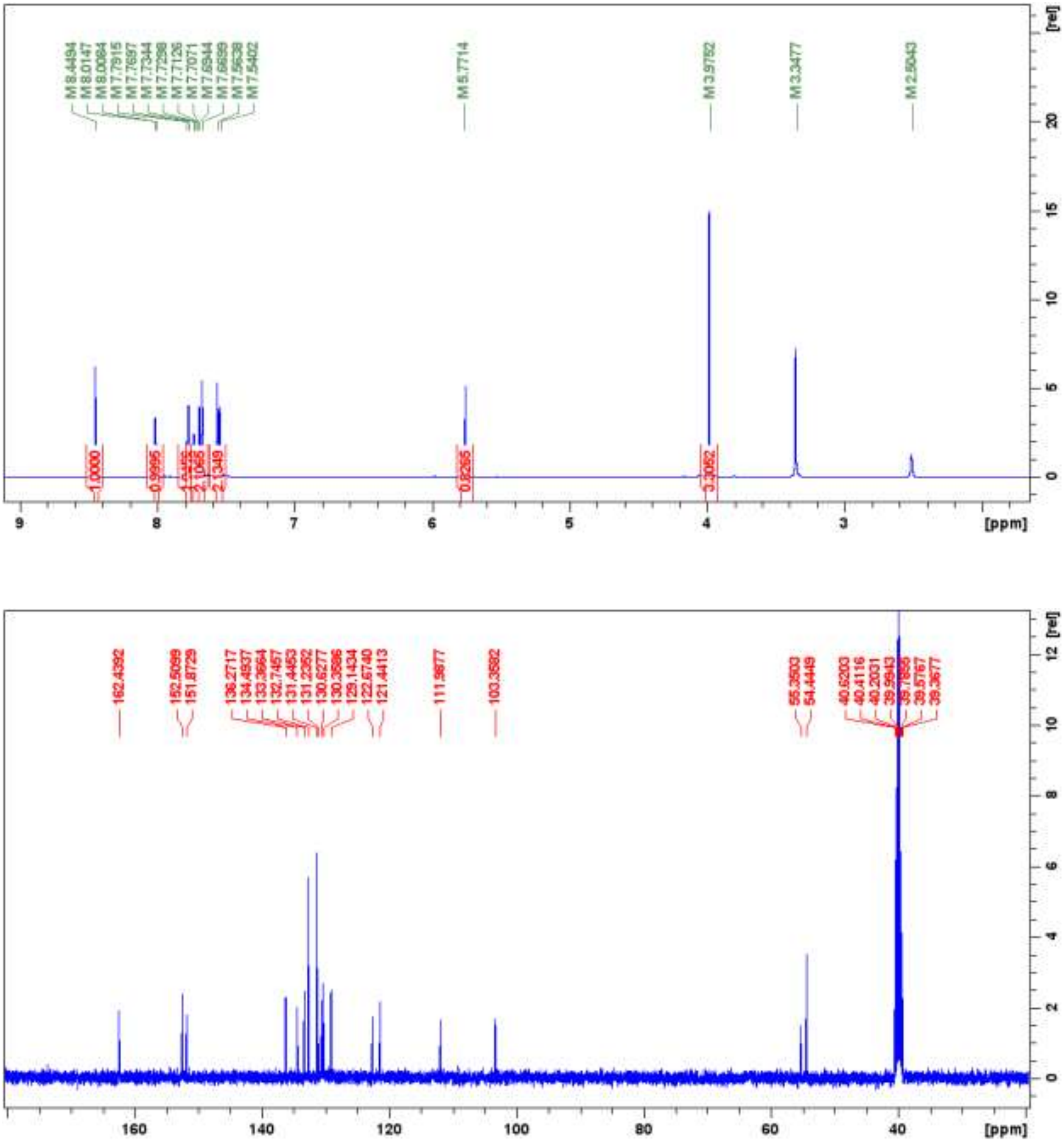

Figure S13. ^{1}H- and ^{13}C NMR spectra of **9a.**

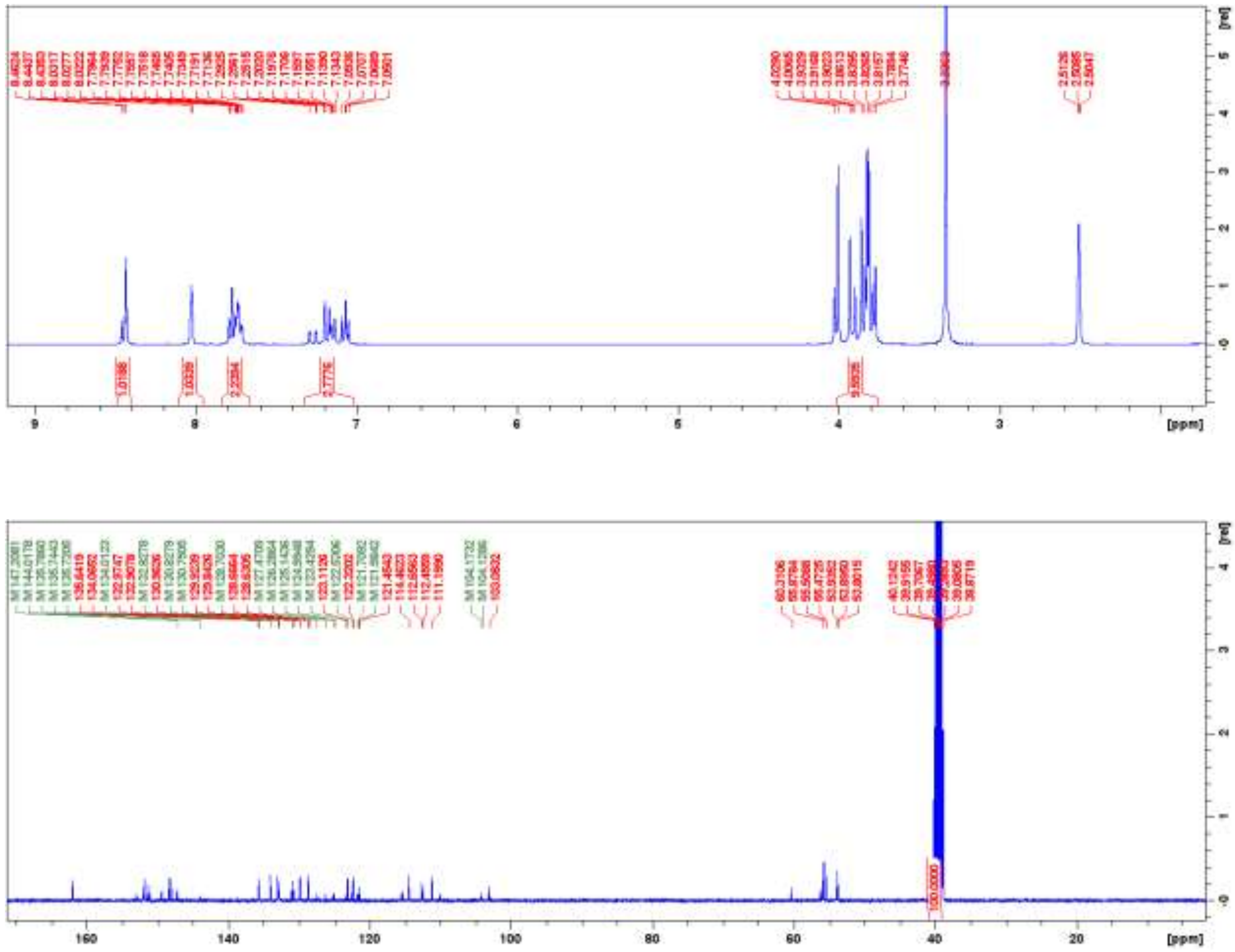

Figure S14. ^{1}H- and ^{13}C NMR spectra of **9b.**

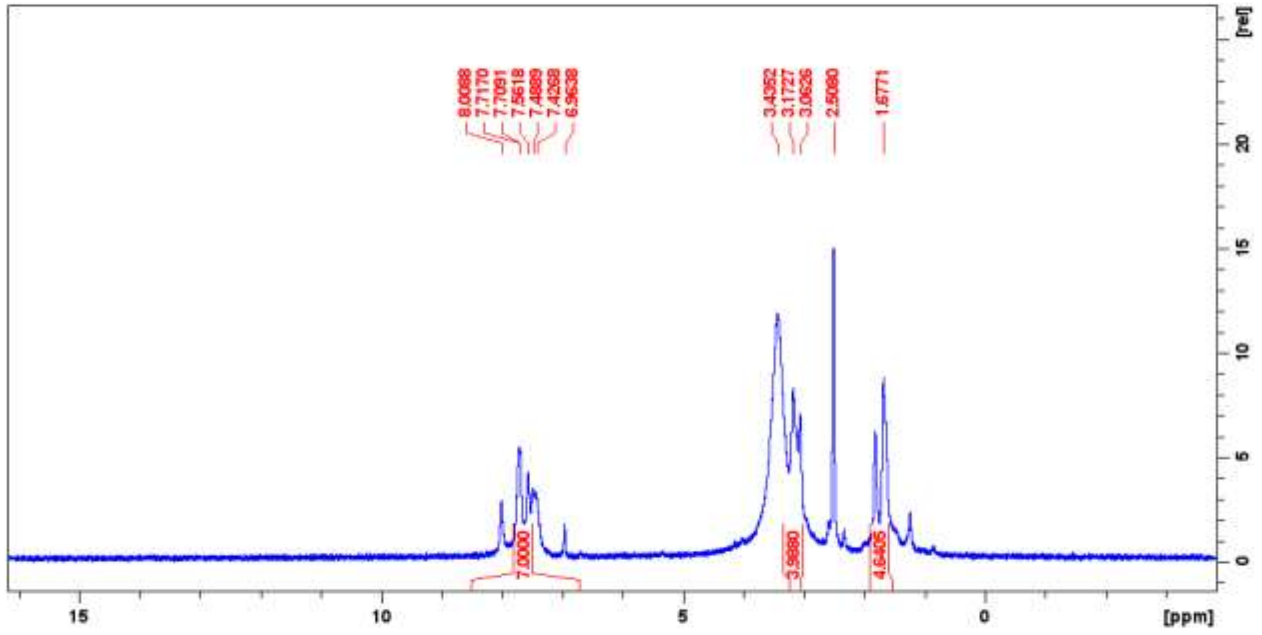

Figure S15. ^{1}H-NMR spectrum of **10**.

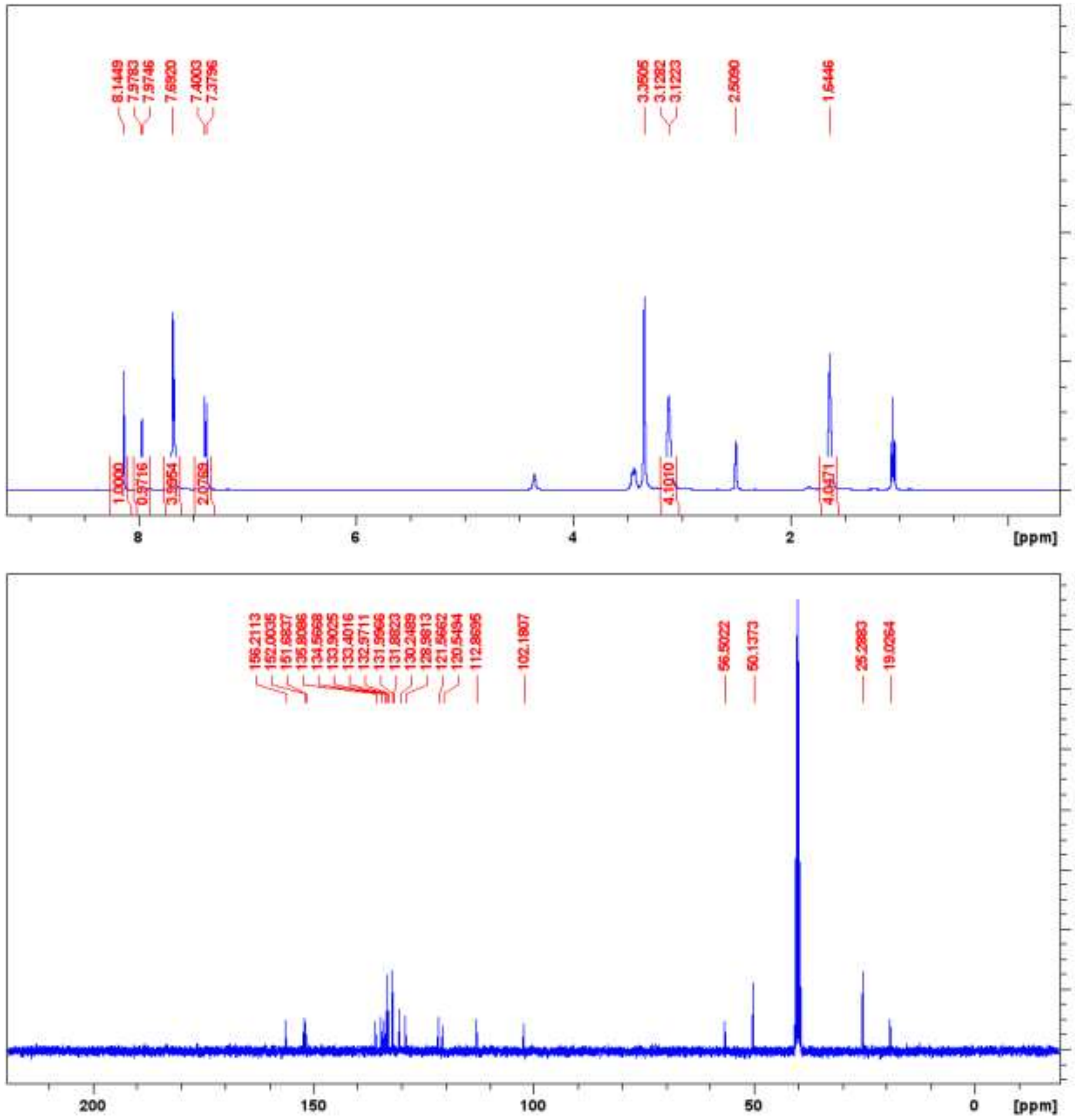

Figure S16. ^{1}H- and ^{13}C NMR spectra of **11a.**

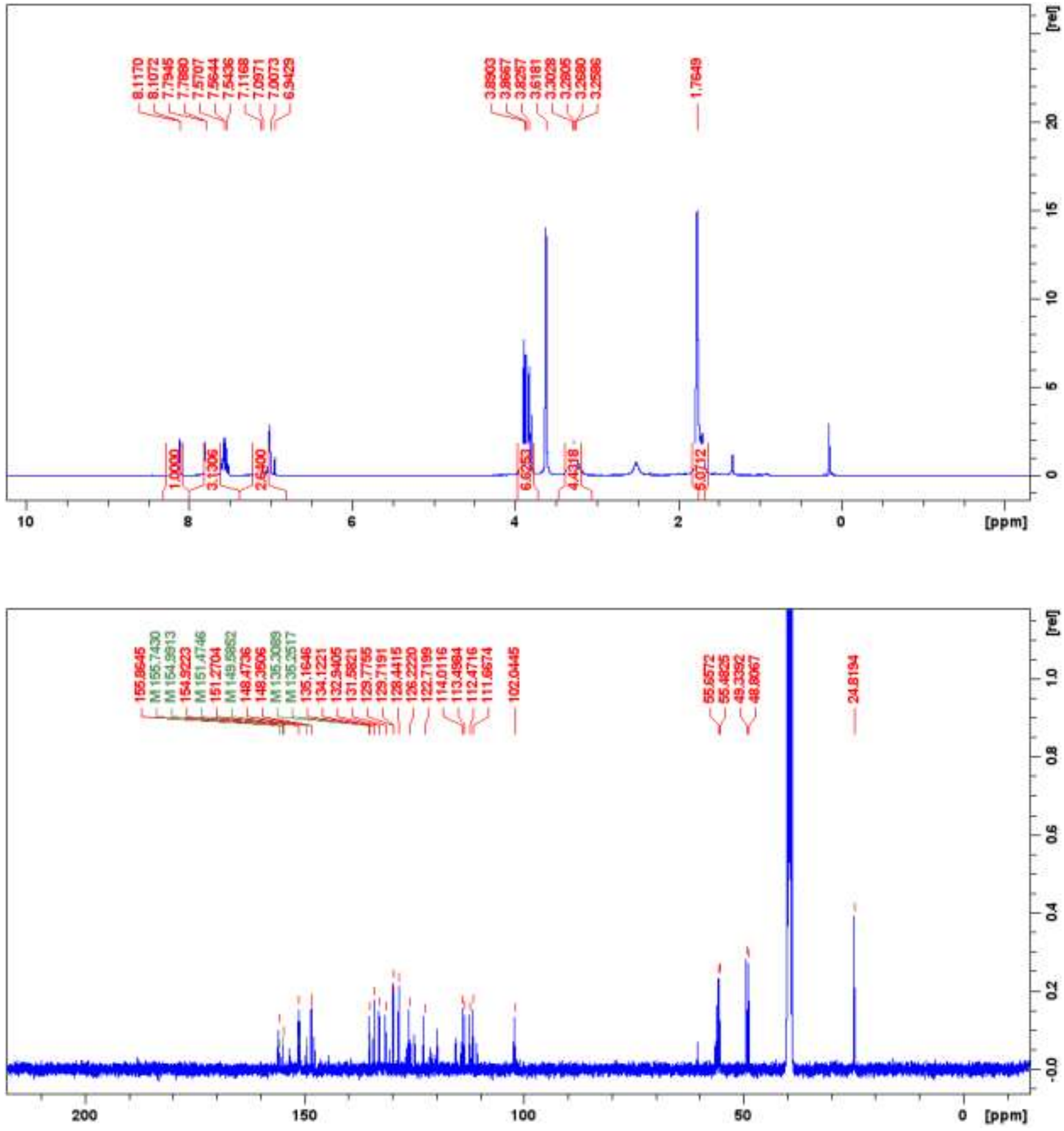

Figure S17. ^{1}H- and ^{13}C NMR spectra of **11b.**

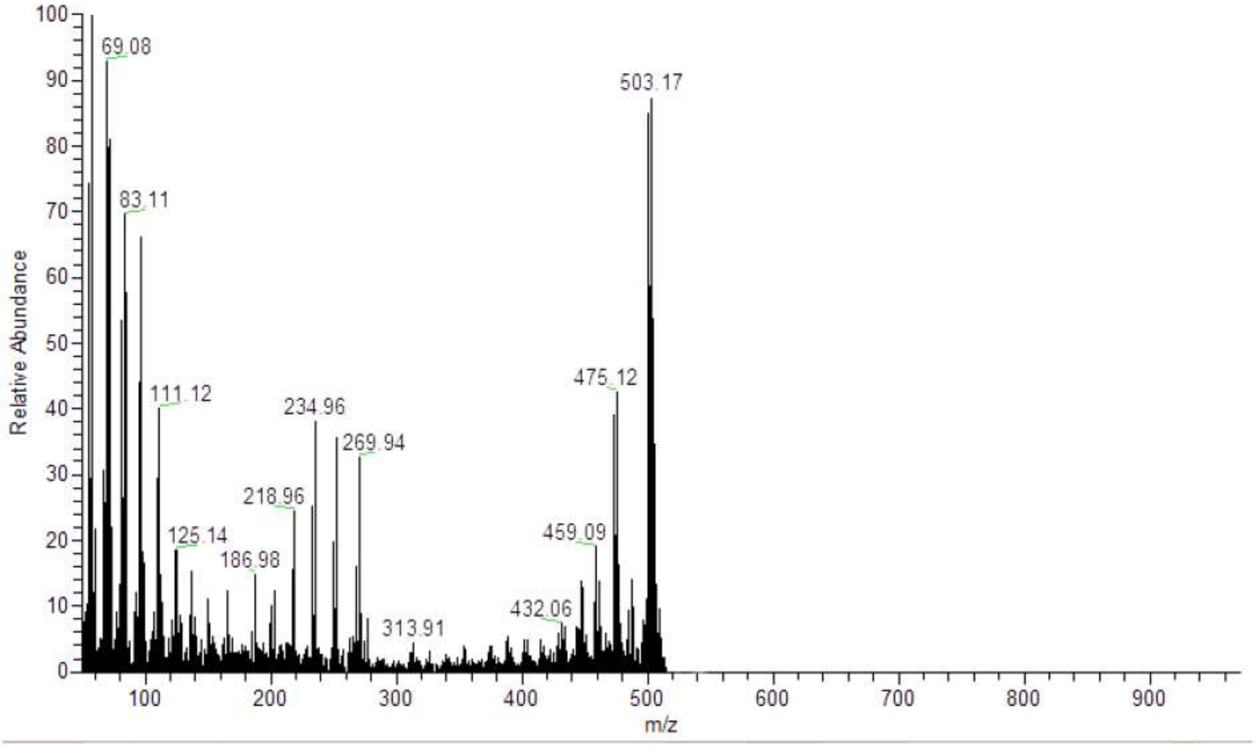

Mass spectrometry of **11b**.

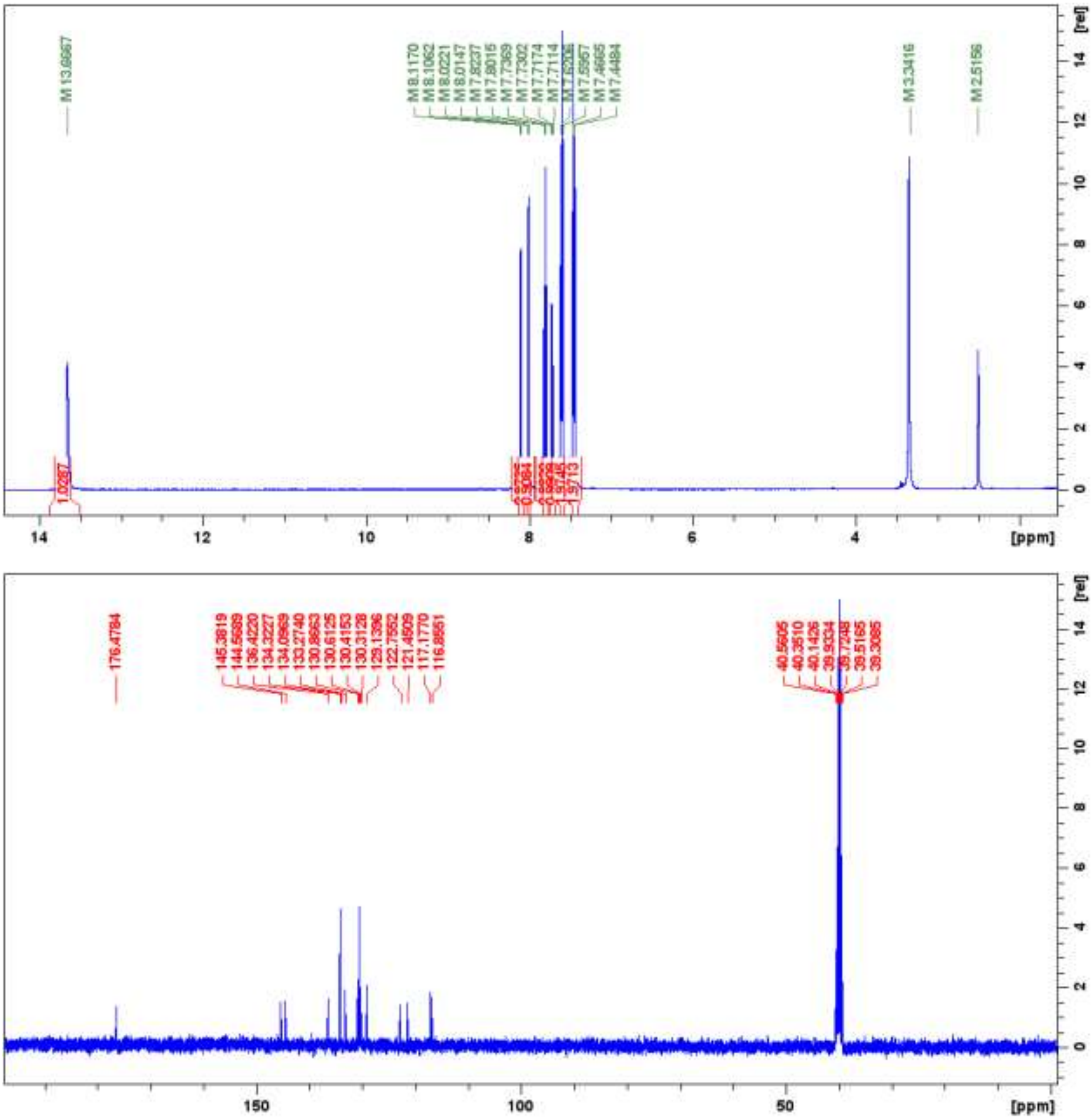

Figure S18. ^{1}H- and ^{13}C NMR spectra of **12.**

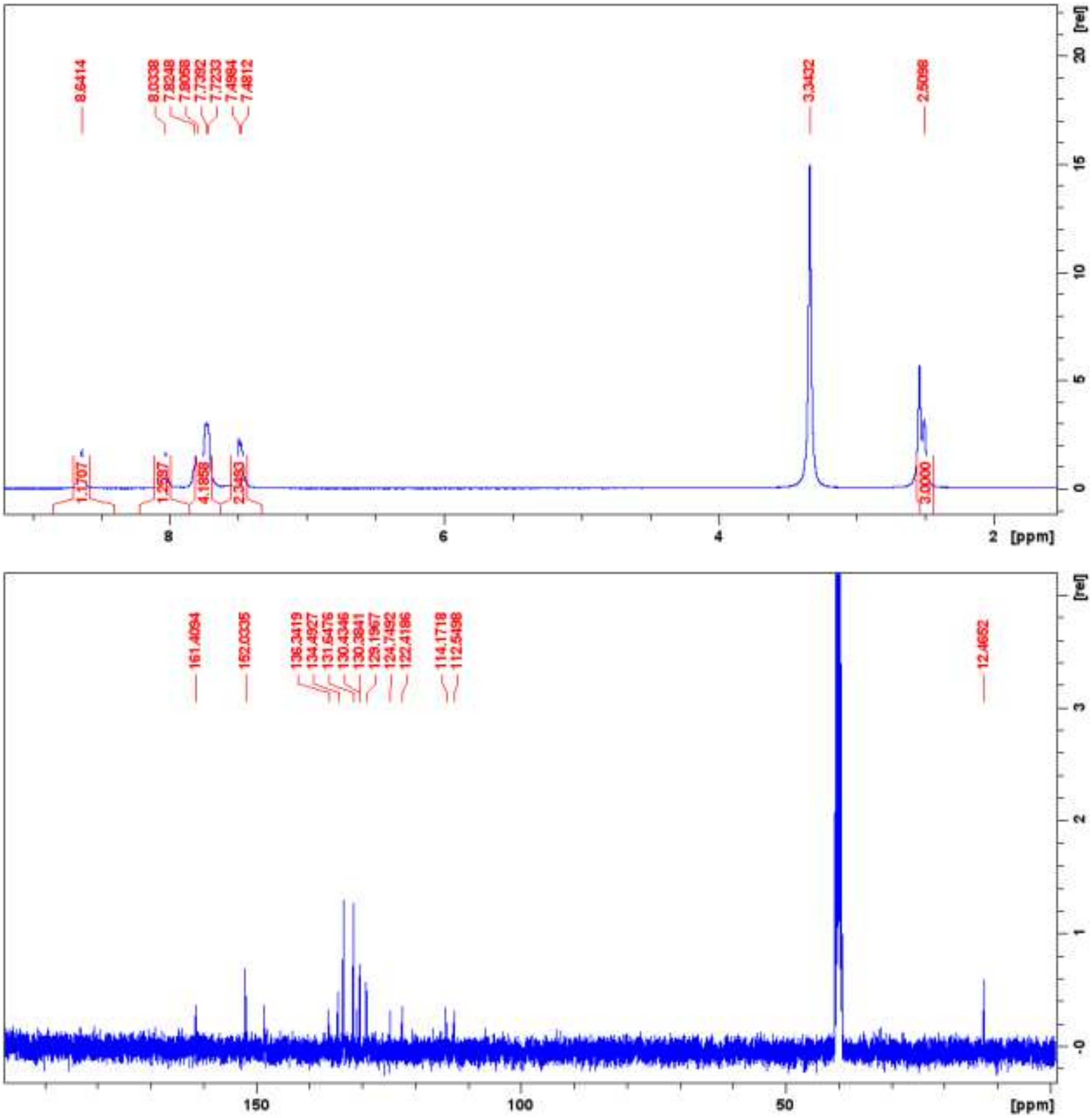

Figure S19. ^{1}H- and ^{13}C NMR spectra of **13**.

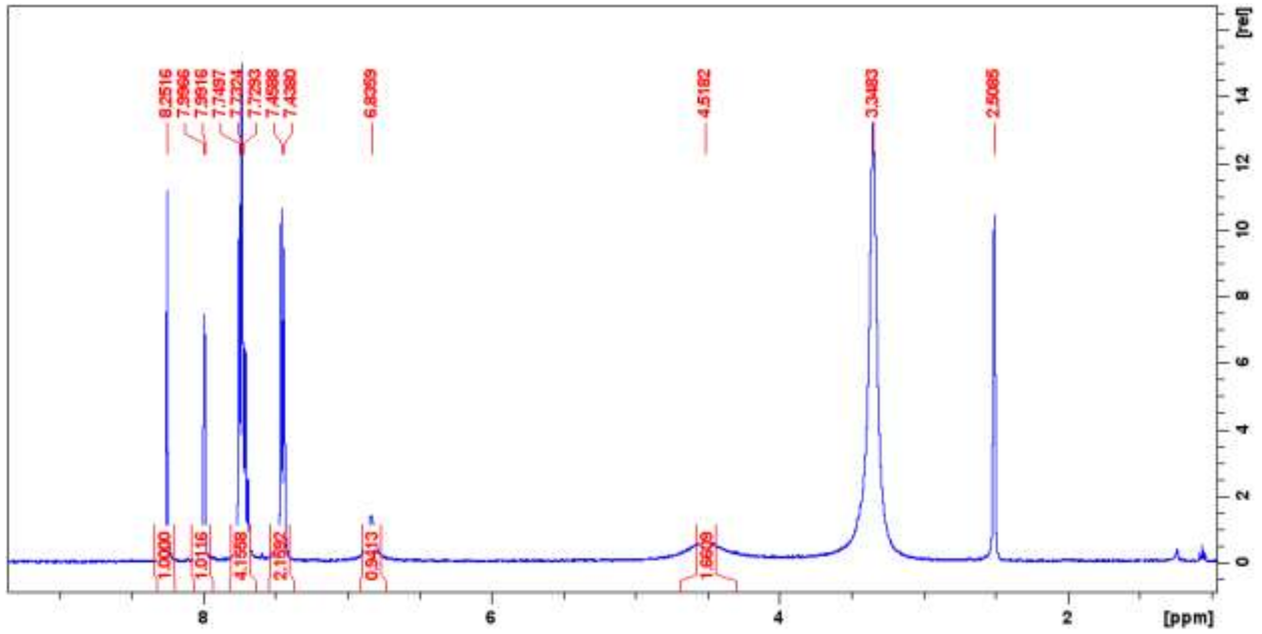

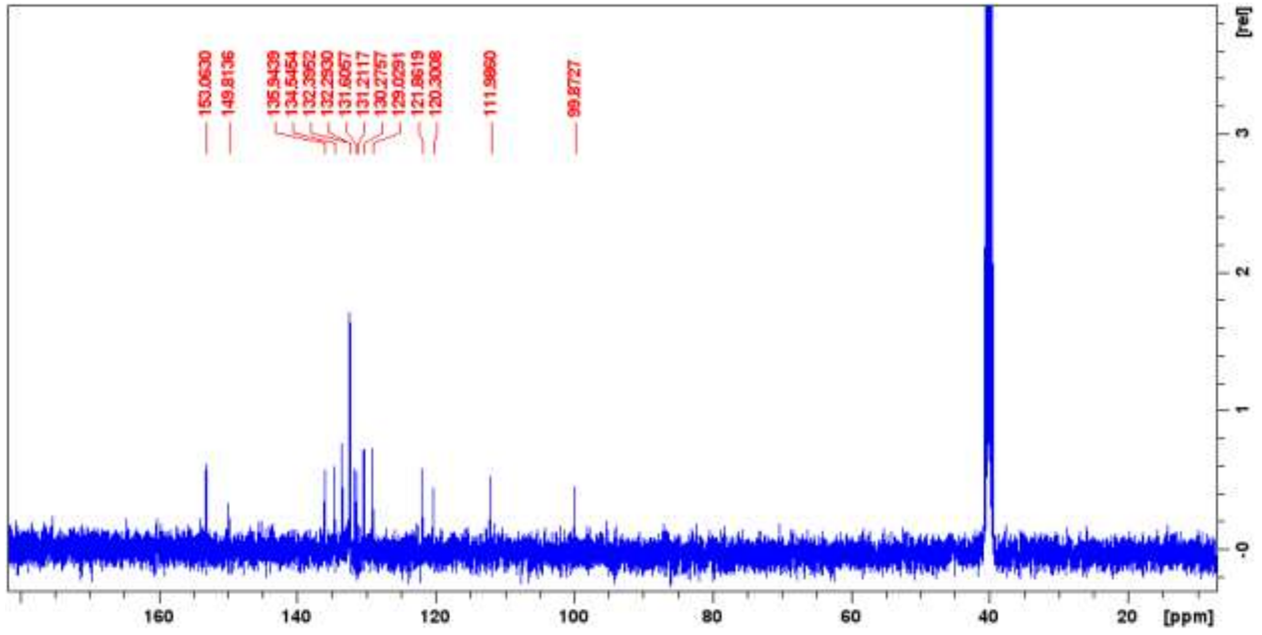

Figure S20. ^{1}H- and ^{13}C NMR spectra of **14a.**

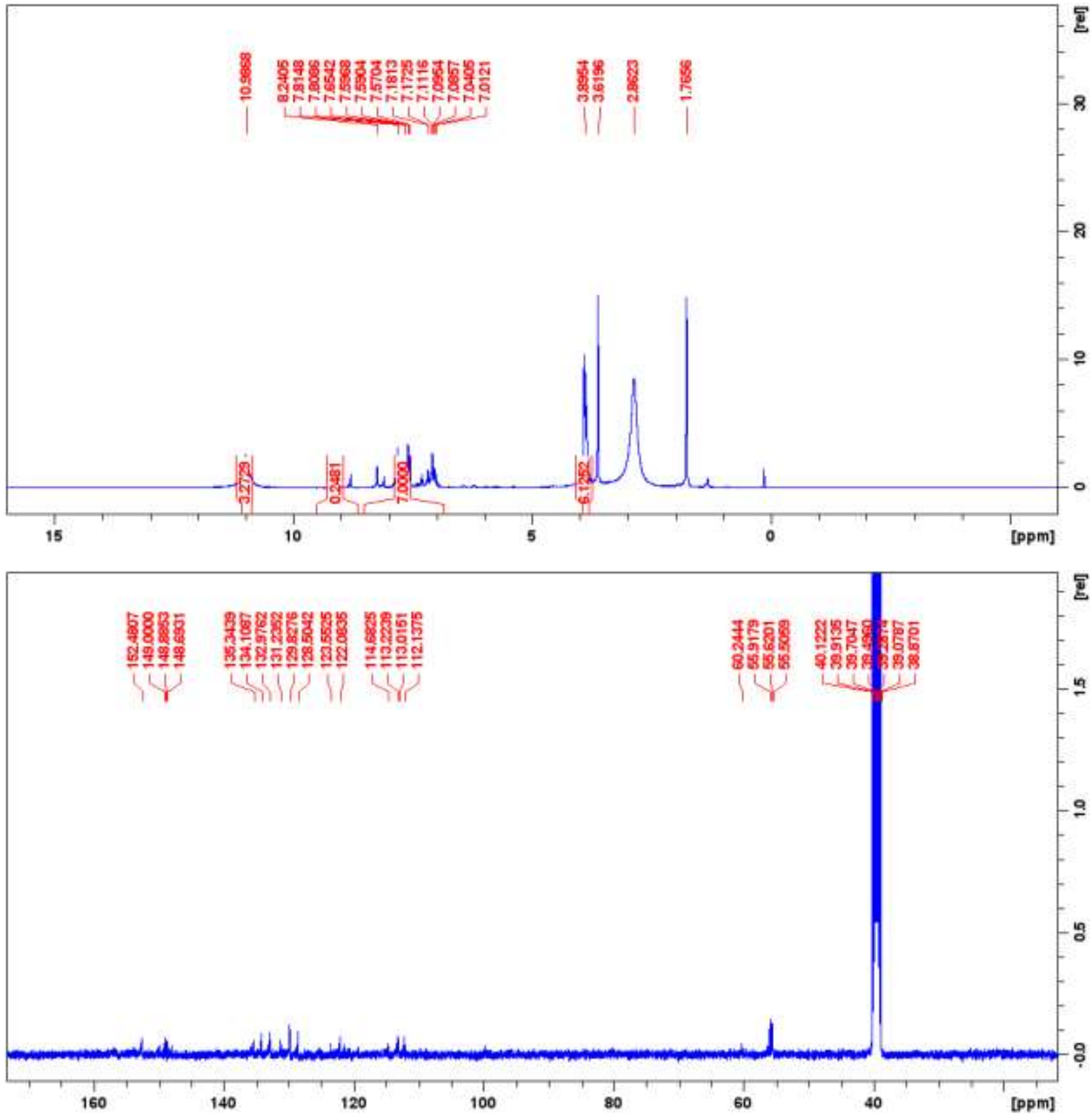

Figure S21. ^{1}H- and ^{13}C NMR spectra of **14b.**

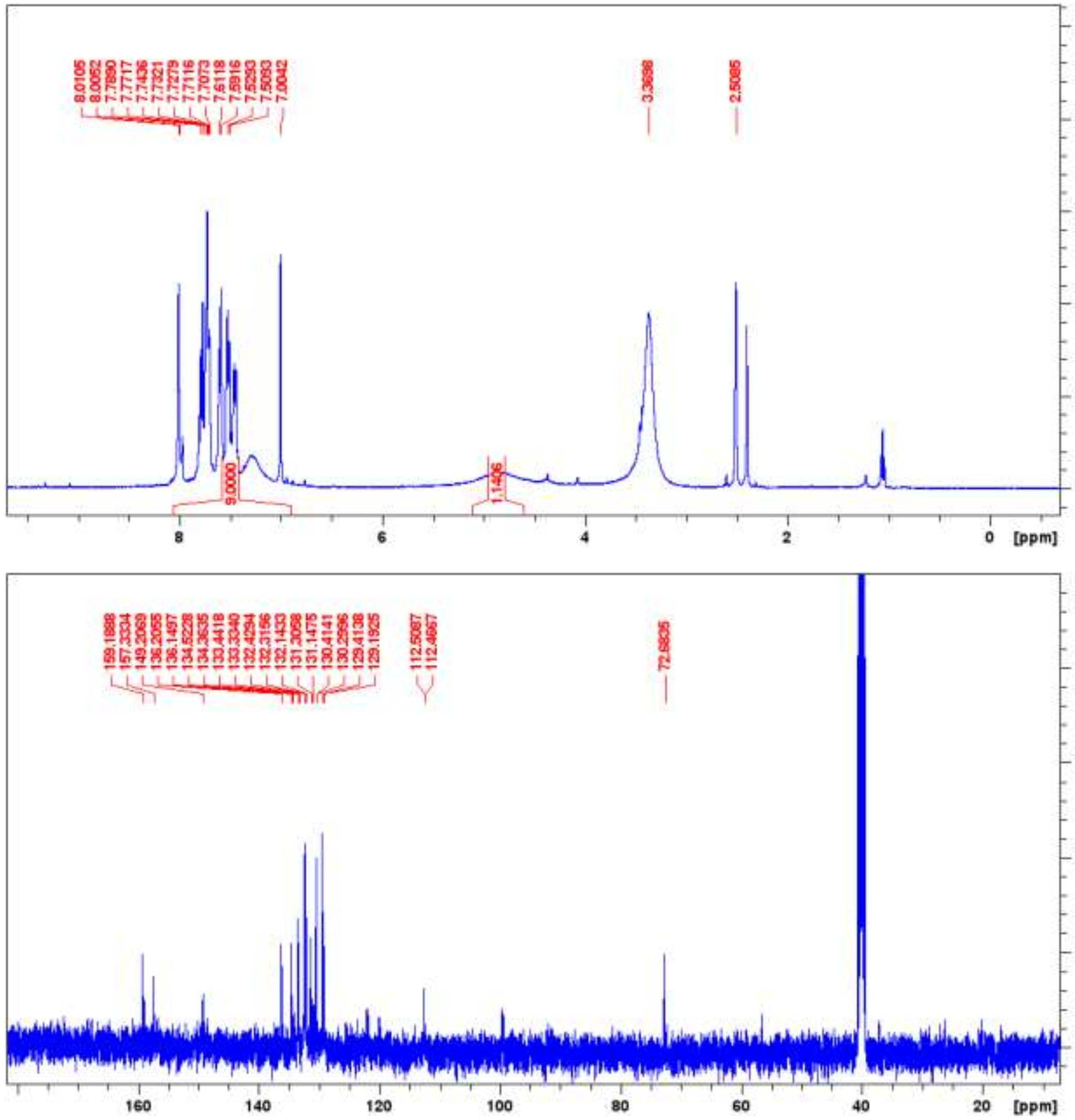

Figure S22. ^{1}H- and ^{13}C NMR spectra of **15**

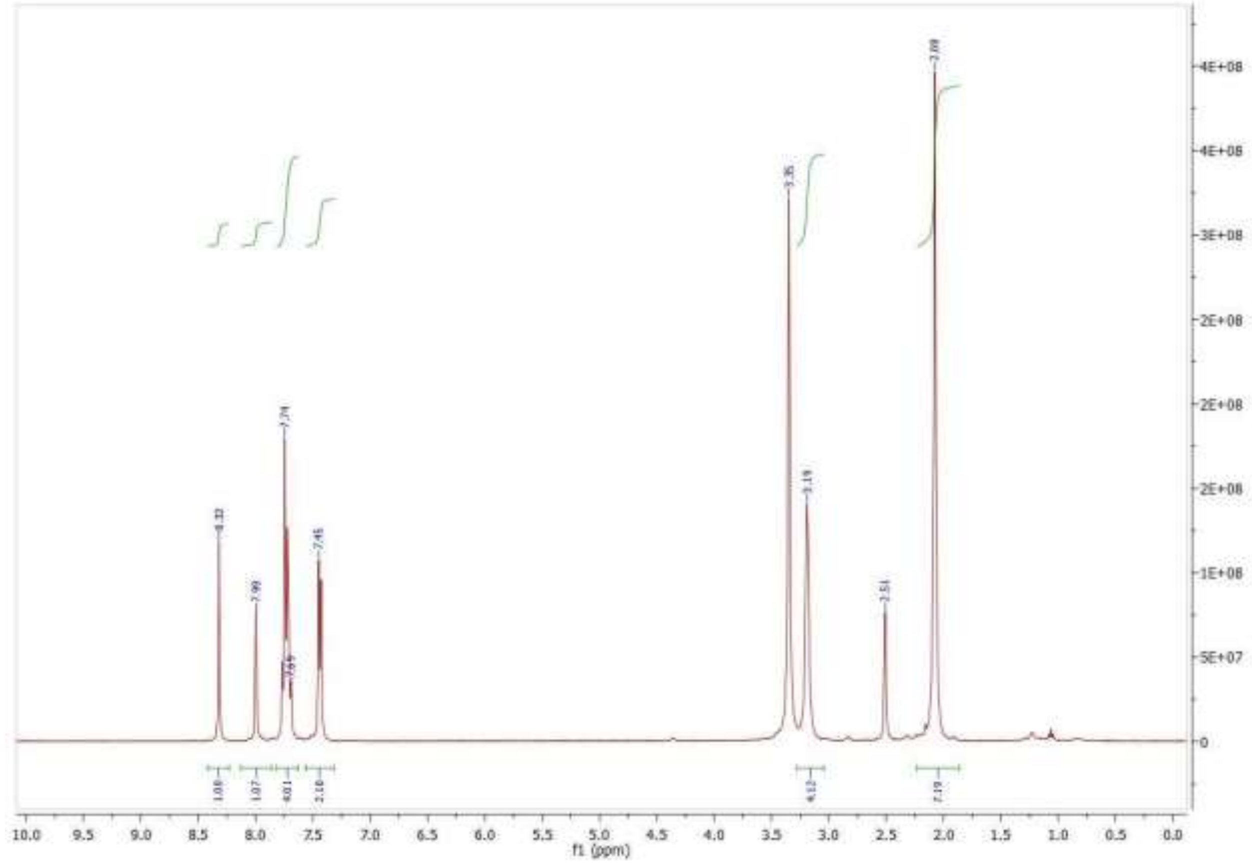
8.32
7.99
7.74
7.69
7.45
3.35
3.19
2.51
2.08
1.08
1.07
4.01
2.10
4.12
7.19
f1 (ppm)

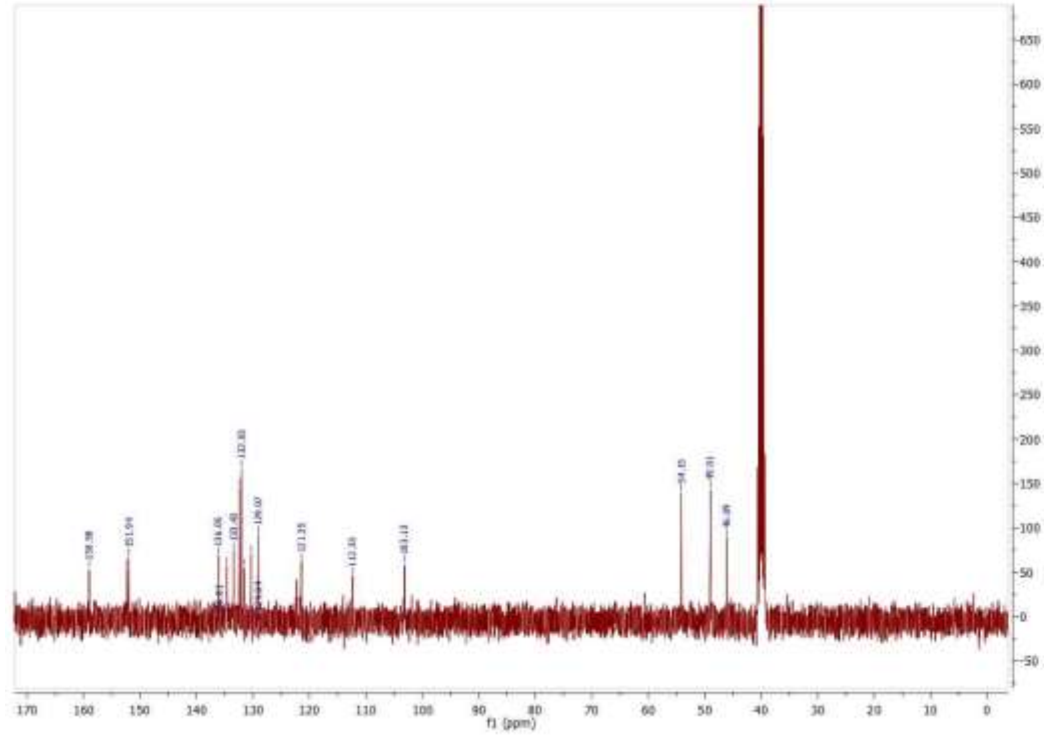
158.58
151.94
134.06
132.40
132.65
129.02
121.35
112.30
103.13
54.15
49.91
46.49
f1 (ppm)

Figure S23. ^{1}H- and ^{13}C NMR spectra of **16a**.

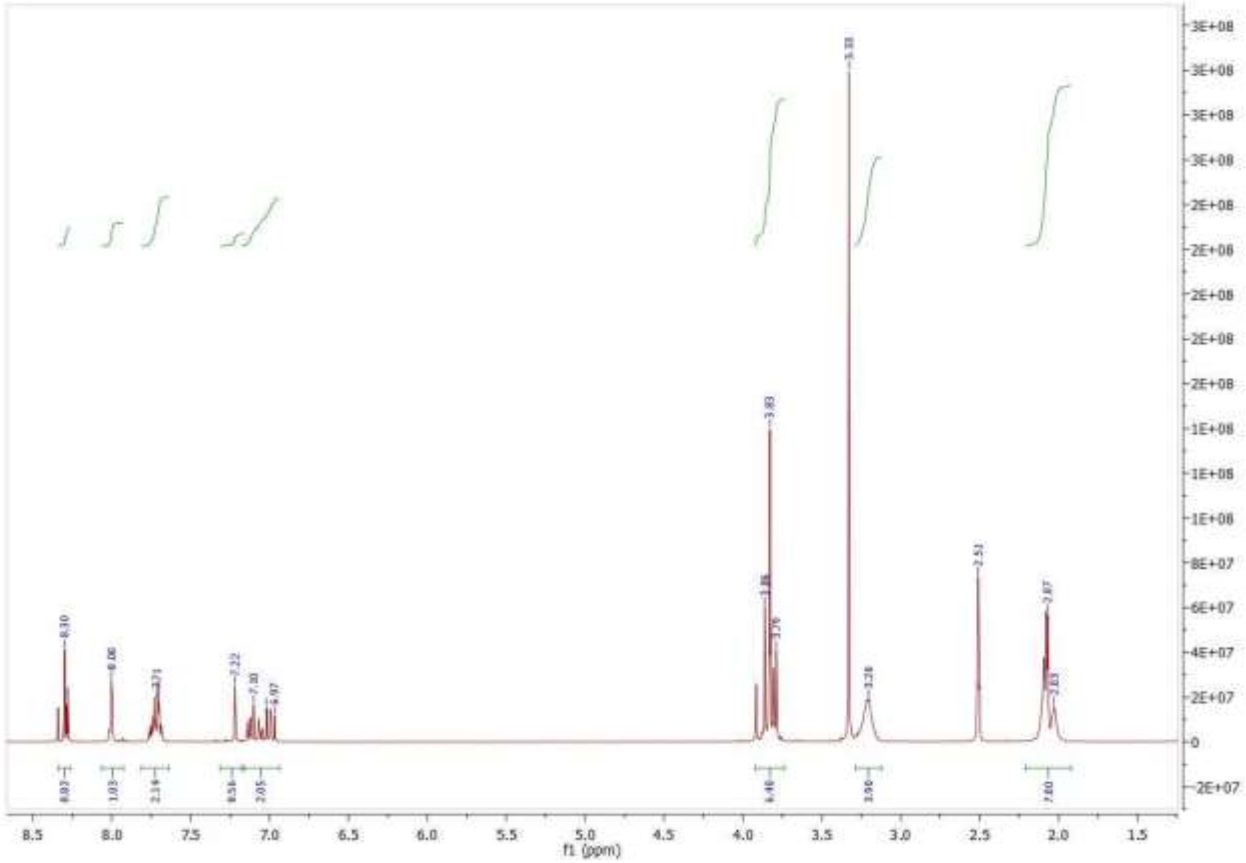

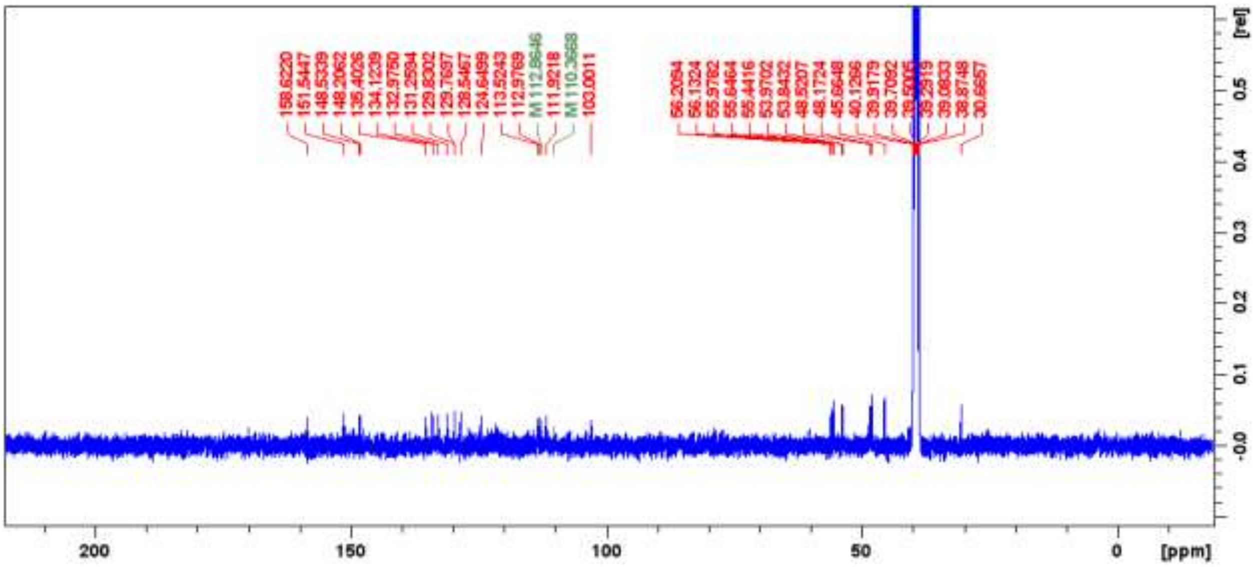

Figure S24. ^{1}H- and ^{13}C NMR spectra of **16b**.

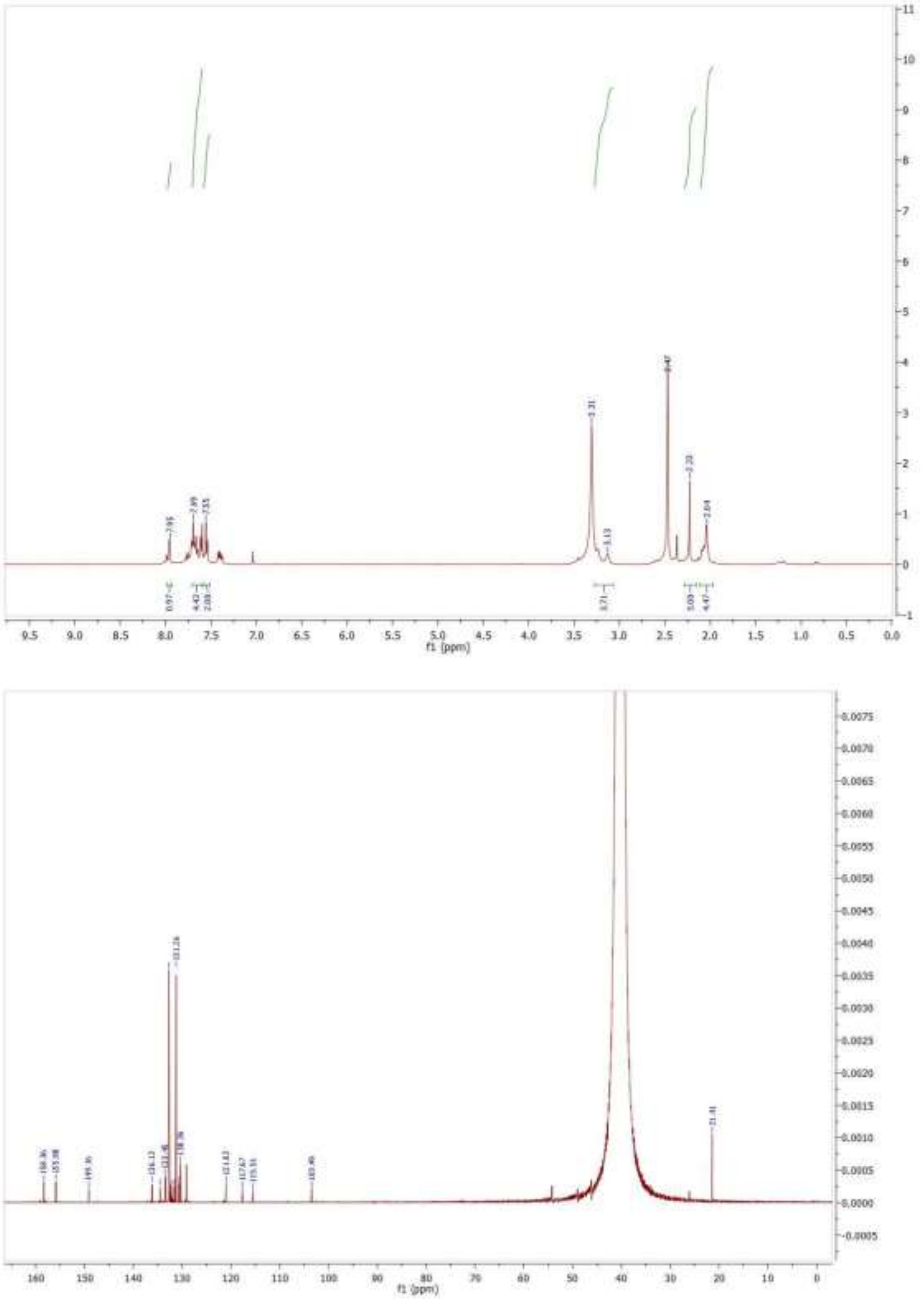

Figure S25. ^{1}H- and ^{13}C NMR spectra of **17**

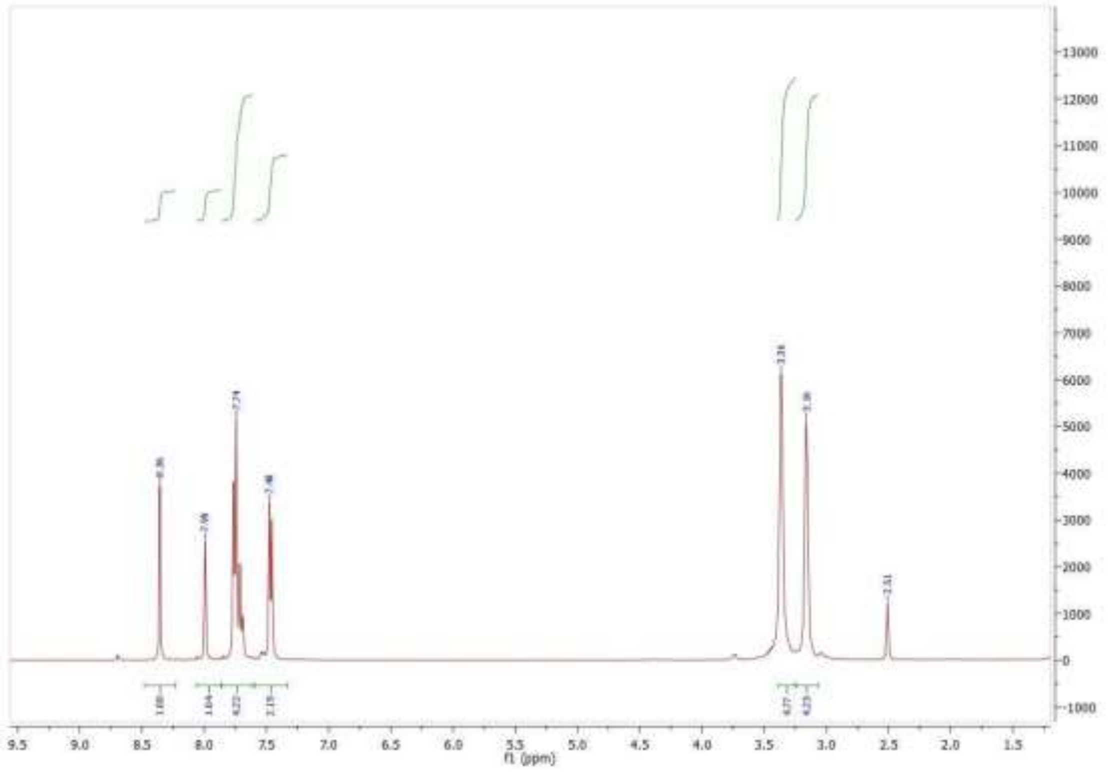

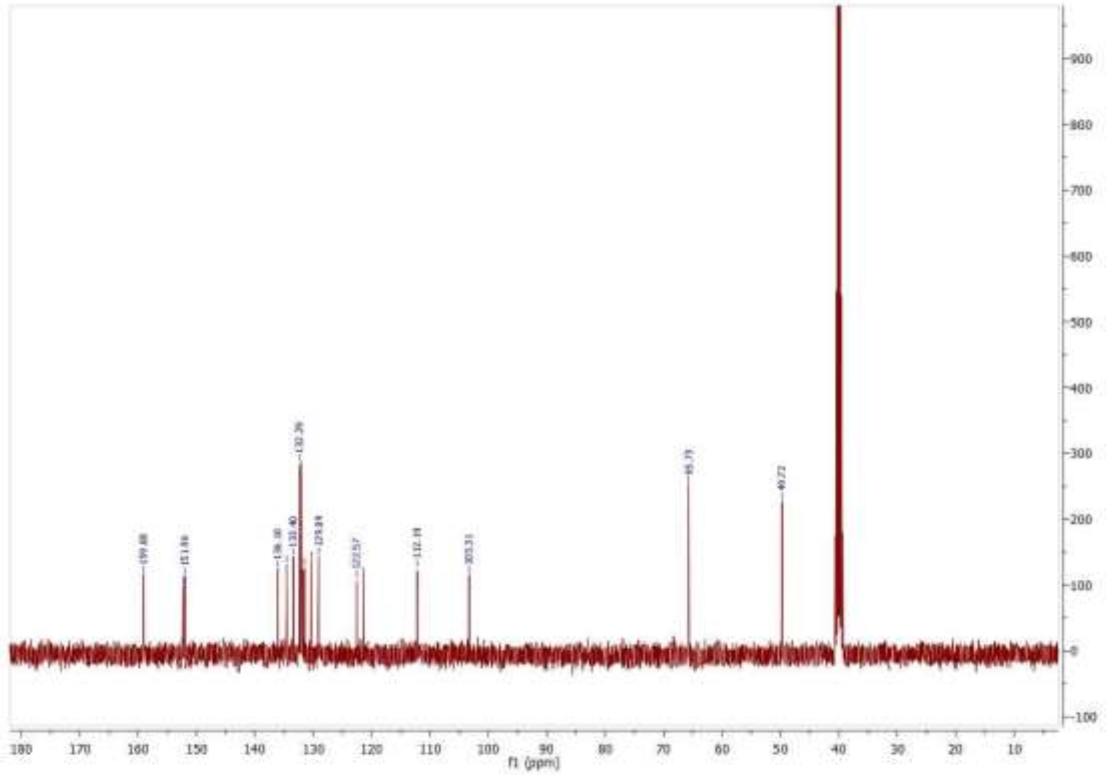

Figure S26. ^{1}H- and ^{13}C NMR spectra of **18a**.

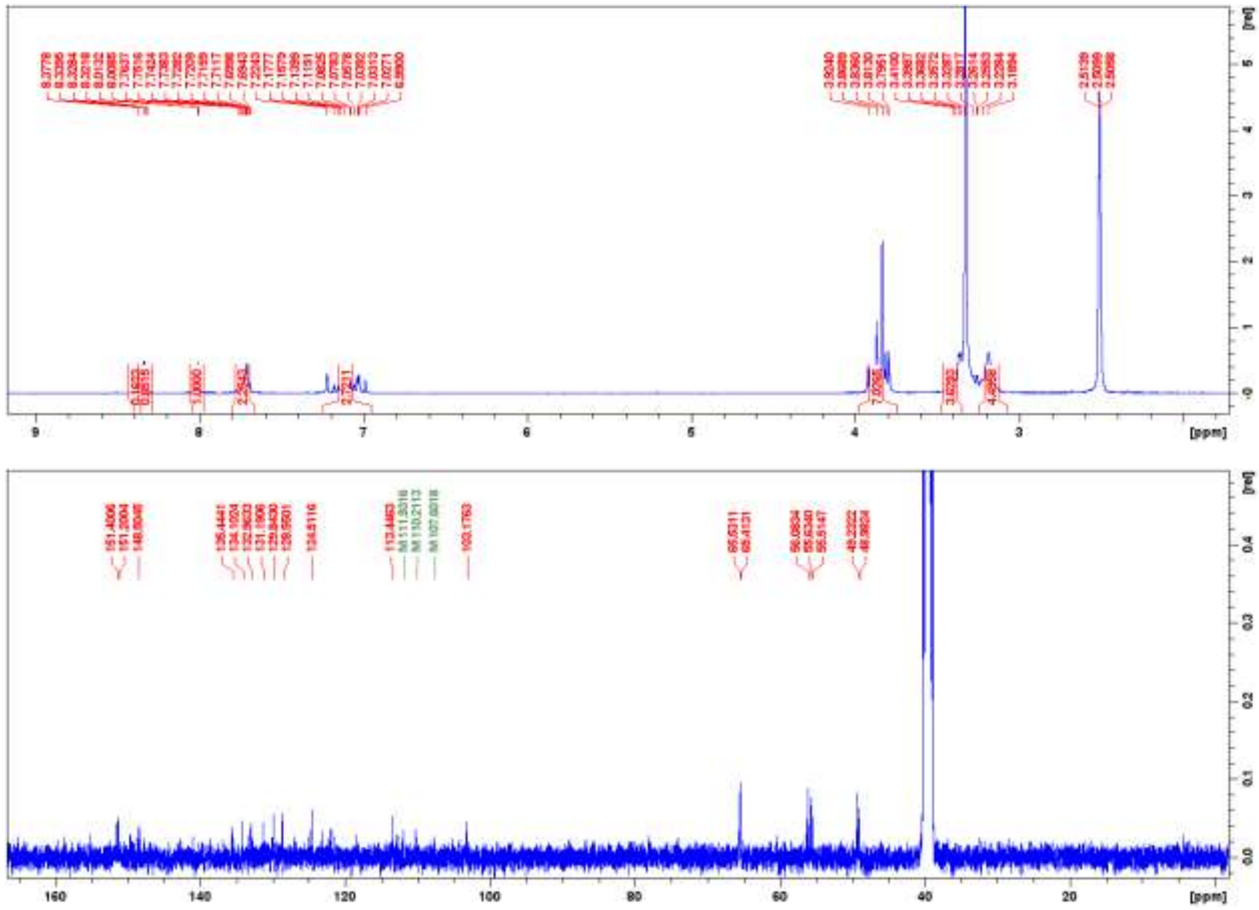

Figure S27. ^{1}H- and ^{13}C NMR spectra of **18b**.

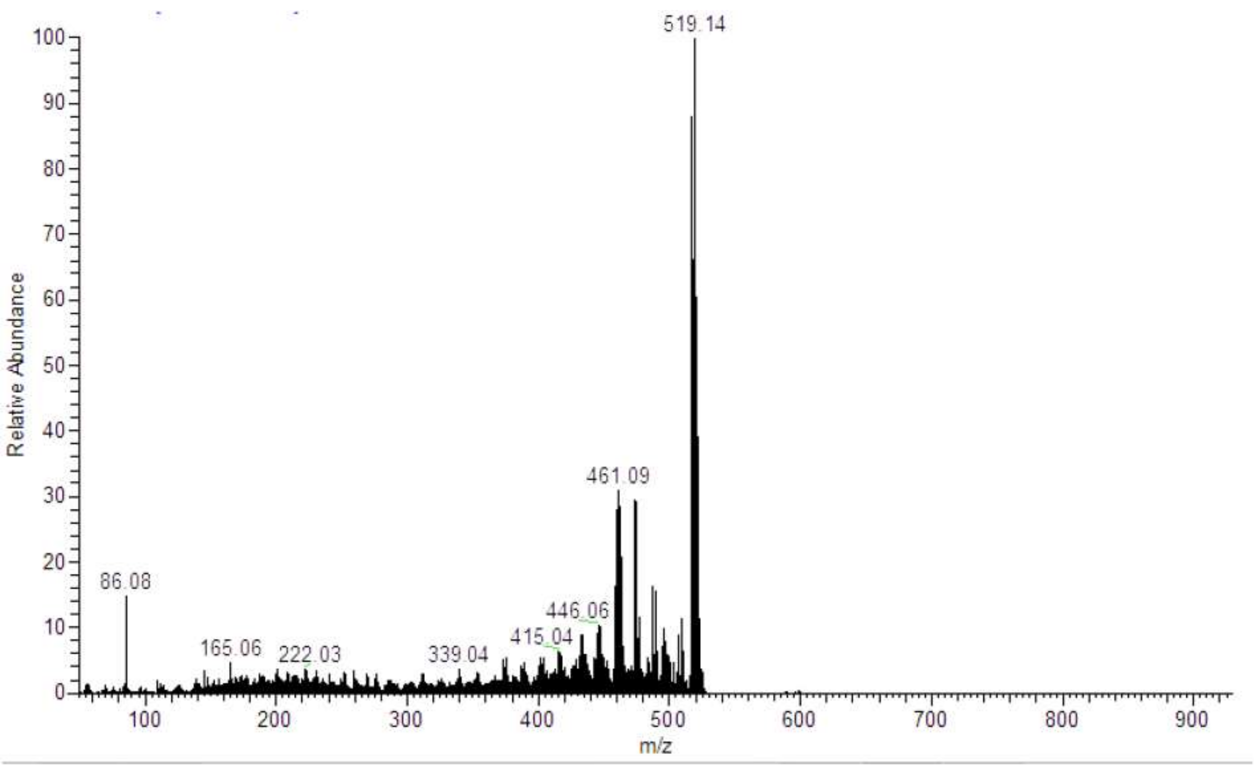

Mass spectrometry of **18b**.

Figure S28. ^{1}H- and ^{13}C NMR spectra of **19**.

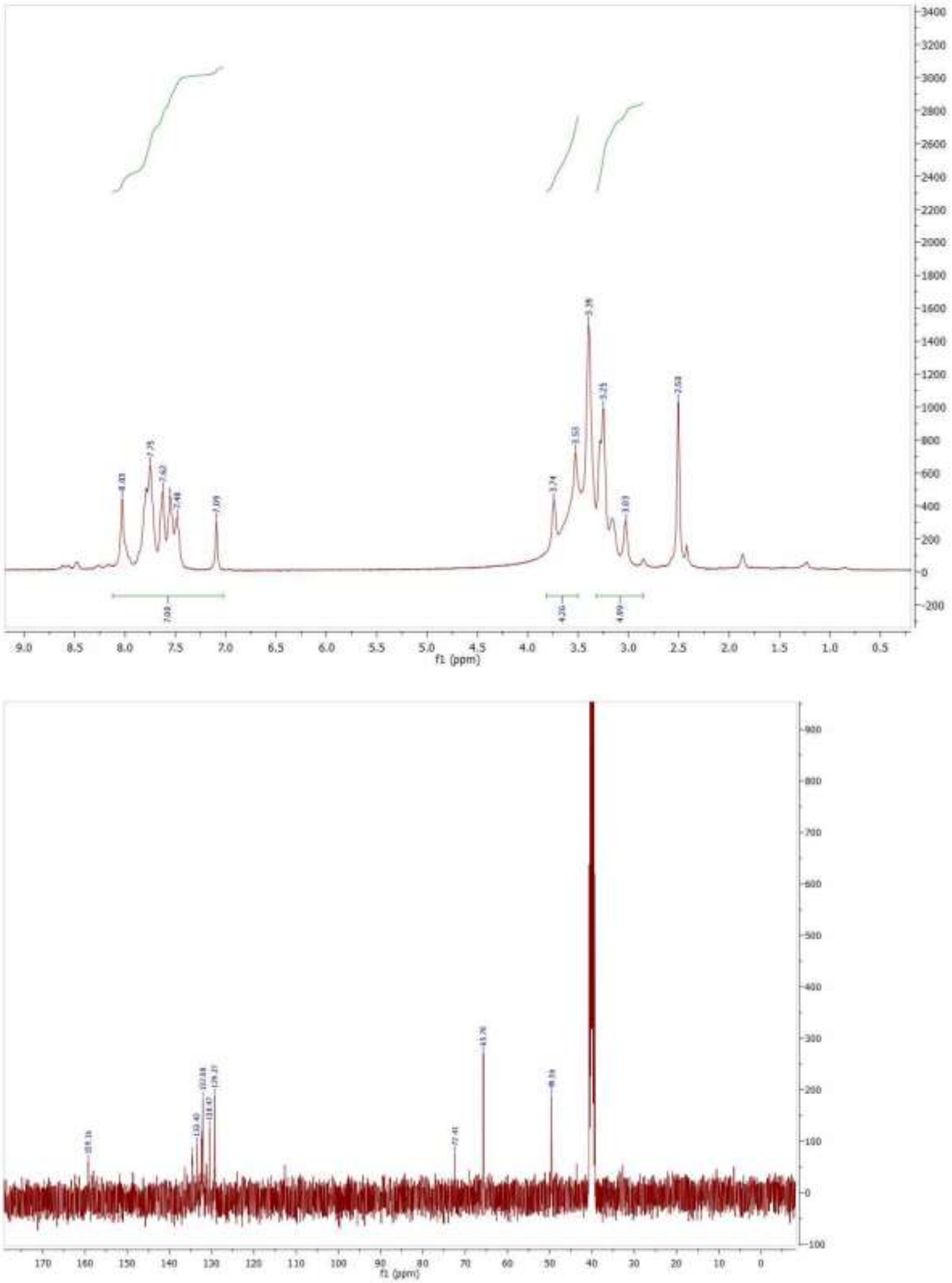

X-ray Structure Report

Datablock: solve4_sq

Bond precision:	C-C = 0.0098 A	Wavelength=1.54178
Cell:	a=17.7407(16) b=13.4659(11) c=17.8663(16)	
	alpha=90 beta=97.594(5) gamma=90	
Temperature:	296 K	
	Calculated	Reported
Volume	4230.7(6)	4230.7(6)
Space group	C 2/c	C 2/c
Hall group	-C 2yc	-C 2yc
Moiety formula	C20 H13 Cl4 N3 O2 [+ solvent]	?
Sum formula	C20 H13 Cl4 N3 O2 [+ solvent]	C20 H13 Cl4 N3 O2
Mr	469.13	469.13
Dx,g cm-3	1.473	1.473
Z	8	8
Mu (mm-1)	5.274	5.274
F000	1904.0	1904.0
F000'	1919.98	
h,k,lmax	21,16,21	21,16,21
Nref	3735	3627
Tmin,Tmax	0.491,0.729	0.850,0.940
Tmin'	0.206	

Correction method= # Reported T Limits: Tmin=0.850 Tmax=0.940 AbsCorr = MULTI-SCAN

Data completeness= 0.971 Theta(max)= 66.589

R(reflections)= 0.0959(2752) wR2(reflections)= 0.2776(3627)

S = 1.059 Npar= 262

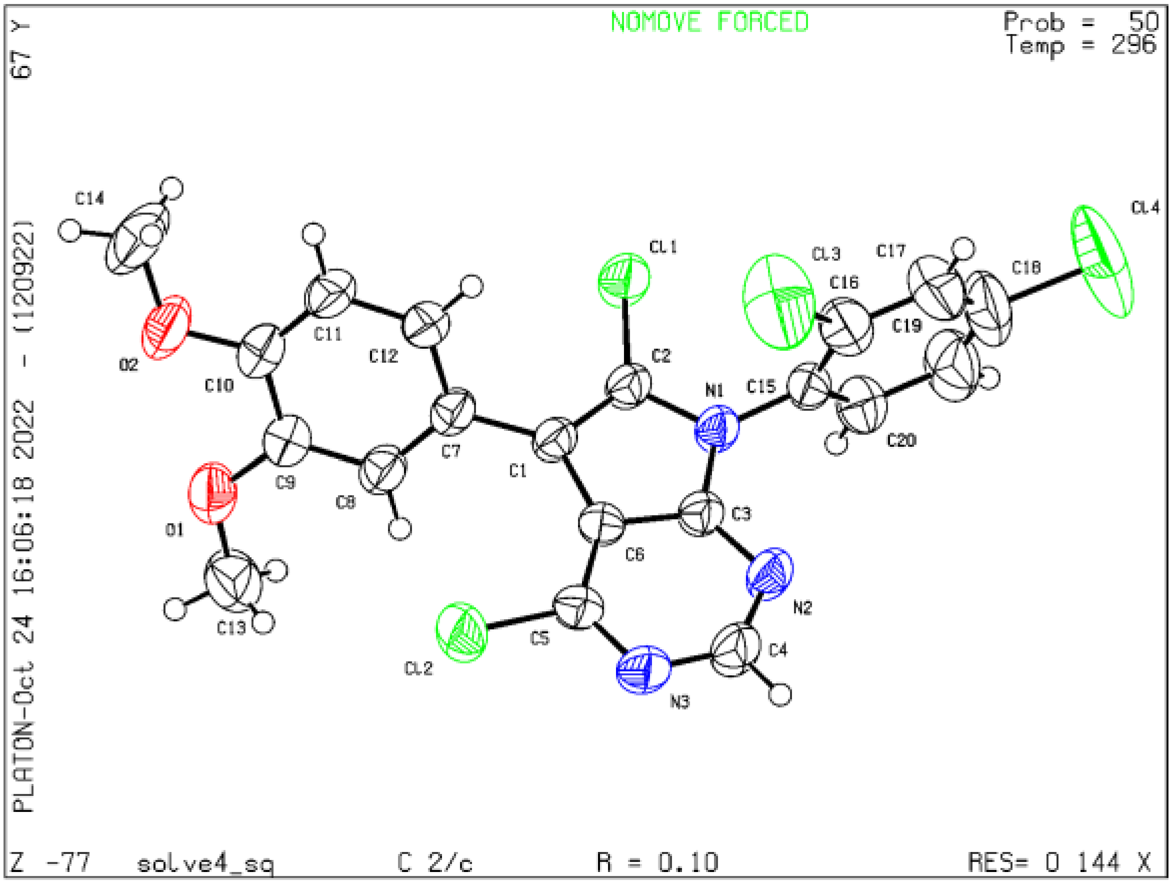

Figure S29. X-ray structure of 4b.

```
data_solve4_sq
_audit_creation_method            'SHELXL-2017/1'
_shelx_SHELXL_version_number      '2017/1'
_chemical_formula_sum
 'C20 H13 Cl4 N3 O2'
_chemical_formula_weight          469.13
loop_
 _atom_type_symbol
 _atom_type_description
 _atom_type_scat_dispersion_real
 _atom_type_scat_dispersion_imag
 _atom_type_scat_source
 'C'  'C'   0.0181   0.0091
 'International Tables Vol C Tables 4.2.6.8 and 6.1.1.4'
 'H'  'H'   0.0000   0.0000
 'International Tables Vol C Tables 4.2.6.8 and 6.1.1.4'
 'Cl'  'Cl'   0.3639   0.7018
 'International Tables Vol C Tables 4.2.6.8 and 6.1.1.4'
 'N'  'N'   0.0311   0.0180
 'International Tables Vol C Tables 4.2.6.8 and 6.1.1.4'
 'O'  'O'   0.0492   0.0322
```

```
 'International Tables Vol C Tables 4.2.6.8 and 6.1.1.4'

_space_group_crystal_system       monoclinic
_space_group_IT_number            15
_space_group_name_H-M_alt         'C 2/c'
_space_group_name_Hall            '-C 2yc'

_shelx_space_group_comment
;
The symmetry employed for this shelxl refinement is uniquely defined
by the following loop, which should always be used as a source of
symmetry information in preference to the above space-group names.
They are only intended as comments.
loop_
 _space_group_symop_operation_xyz
 'x, y, z'
 '-x, y, -z+1/2'
 'x+1/2, y+1/2, z'
 '-x+1/2, y+1/2, -z+1/2'
 '-x, -y, -z'
 'x, -y, z-1/2'
 '-x+1/2, -y+1/2, -z'
 'x+1/2, -y+1/2, z-1/2'
_cell_length_a                    17.7407(16)
_cell_length_b                    13.4659(11)
_cell_length_c                    17.8663(16)
_cell_angle_alpha                 90
_cell_angle_beta                  97.594(5)
_cell_angle_gamma                 90
_cell_volume                      4230.7(6)
_cell_formula_units_Z             8
_cell_measurement_temperature     296(2)
_cell_measurement_reflns_used      7819
_cell_measurement_theta_min        4.14
_cell_measurement_theta_max       66.20
_exptl_crystal_description       'Block'
_exptl_crystal_colour            'yellow'
_exptl_crystal_density_meas       ?
_exptl_crystal_density_method     ?
_exptl_crystal_density_diffrn     1.473
_exptl_crystal_F_000              1904
_exptl_transmission_factor_min    ?
_exptl_transmission_factor_max    ?
_exptl_crystal_size_max           0.290
_exptl_crystal_size_mid           0.120
_exptl_crystal_size_min           0.060
_exptl_absorpt_coefficient_mu     5.274
_shelx_estimated_absorpt_T_min    0.310
_shelx_estimated_absorpt_T_max    0.743
_exptl_absorpt_correction_type          Multi-Scan
_exptl_absorpt_correction_T_min         0.85
_exptl_absorpt_correction_T_max         0.94
```

```
_exptl_absorpt_process_details
SADABS-2016/2 - Bruker AXS area detector scaling and absorption
correction
_diffrn_ambient_temperature       296(2)
_diffrn_radiation_wavelength      1.54178
_diffrn_radiation_type            CuK\a
_diffrn_measurement_device_type  'Bruker APEX-II CCD'
_diffrn_measurement_method       '\f and \w scans'
_diffrn_detector_area_resol_mean        8.3333
_diffrn_reflns_number             26223
_diffrn_reflns_av_unetI/netI      0.0590
_diffrn_reflns_av_R_equivalents   0.0872
_diffrn_reflns_limit_h_min        -17
_diffrn_reflns_limit_h_max        21
_diffrn_reflns_limit_k_min        -16
_diffrn_reflns_limit_k_max        16
_diffrn_reflns_limit_l_min        -21
_diffrn_reflns_limit_l_max        20
_diffrn_reflns_theta_min          4.135
_diffrn_reflns_theta_max          66.589
_diffrn_reflns_theta_full         66.589
_diffrn_measured_fraction_theta_max   0.971
_diffrn_measured_fraction_theta_full  0.971
_diffrn_reflns_Laue_measured_fraction_max    0.971
_diffrn_reflns_Laue_measured_fraction_full   0.971
_diffrn_reflns_point_group_measured_fraction_max   0.971
_diffrn_reflns_point_group_measured_fraction_full  0.971
_reflns_number_total              3627
_reflns_number_gt                 2752
_reflns_threshold_expression      'I > 2\s(I)'
_reflns_Friedel_coverage          0.000
_reflns_Friedel_fraction_max      .
_reflns_Friedel_fraction_full     .

_reflns_special_details
 Reflections were merged by SHELXL according to the crystal
 class for the calculation of statistics and refinement.
 Structure factors included contributions from the .fab file.
 _reflns_Friedel_fraction is defined as the number of unique
 Friedel pairs measured divided by the number that would be
 possible theoretically, ignoring centric projections and
 systematic absences.

_computing_data_collection        ?
_computing_cell_refinement        ?
_computing_data_reduction         ?
_computing_structure_solution     ?
_computing_structure_refinement 'SHELXL-2017/1 (Sheldrick, 2017)'
_computing_molecular_graphics     ?
_computing_publication_material   ?
_refine_special_details          ?
_refine_ls_structure_factor_coef  Fsqd
```

```
_refine_ls_matrix_type            full
_refine_ls_weighting_scheme       calc
_refine_ls_weighting_details
'w=1/[\s^2^(Fo^2^)+(0.1212P)^2^+32.4683P] where P=(Fo^2^+2Fc^2^)/3'
_atom_sites_solution_primary      ?
_atom_sites_solution_secondary    ?
_atom_sites_solution_hydrogens    geom
_refine_ls_hydrogen_treatment     constr
_refine_ls_extinction_method      none
_refine_ls_extinction_coef        .
_refine_ls_number_reflns          3627
_refine_ls_number_parameters      262
_refine_ls_number_restraints      0
_refine_ls_R_factor_all           0.1165
_refine_ls_R_factor_gt            0.0959
_refine_ls_wR_factor_ref          0.2776
_refine_ls_wR_factor_gt           0.2604
_refine_ls_goodness_of_fit_ref    1.059
_refine_ls_restrained_S_all       1.059
_refine_ls_shift/su_max           0.000
_refine_ls_shift/su_mean          0.000

loop_
 _atom_site_label
 _atom_site_type_symbol
 _atom_site_fract_x
 _atom_site_fract_y
 _atom_site_fract_z
 _atom_site_U_iso_or_equiv
 _atom_site_adp_type
 _atom_site_occupancy
 _atom_site_site_symmetry_order
 _atom_site_calc_flag
 _atom_site_refinement_flags_posn
 _atom_site_refinement_flags_adp
 _atom_site_refinement_flags_occupancy
 _atom_site_disorder_assembly
 _atom_site_disorder_group
Cl1 Cl 0.60140(12) 0.59461(12) 0.50361(8) 0.0680(6) Uani 1 1 d . .
O1 O 0.5181(3) 0.1273(4) 0.4887(3) 0.0730(14) Uani 1 1 d . . . . .
N1 N 0.5968(3) 0.6110(3) 0.3551(2) 0.0470(11) Uani 1 1 d . . . . .
C1 C 0.6044(3) 0.4488(4) 0.3947(3) 0.0428(12) Uani 1 1 d . . . . .
Cl2 Cl 0.61139(14) 0.26273(12) 0.26465(10) 0.0780(6) Uani 1 1 d . .
O2 O 0.6263(3) 0.1280(4) 0.5993(3) 0.0792(15) Uani 1 1 d . . . . .
N2 N 0.5946(3) 0.5900(4) 0.2217(3) 0.0579(14) Uani 1 1 d . . . . .
C2 C 0.6018(3) 0.5462(4) 0.4159(3) 0.0467(13) Uani 1 1 d . . . . .
Cl3 Cl 0.75667(13) 0.6780(2) 0.3825(2) 0.1281(12) Uani 1 1 d . . . . .
N3 N 0.6002(3) 0.4210(4) 0.1813(3) 0.0600(14) Uani 1 1 d . . . . .
C3 C 0.5971(3) 0.5540(4) 0.2918(3) 0.0449(13) Uani 1 1 d . . . . .
Cl4 Cl 0.6411(3) 1.04467(18) 0.3555(3) 0.1801(19) Uani 1 1 d . . . . .
C4 C 0.5958(5) 0.5197(5) 0.1707(3) 0.0645(18) Uani 1 1 d . . . . .
H4 H 0.593293 0.541061 0.120972 0.077 Uiso 1 1 calc R U . . .
```

```
C5 C 0.6031(4) 0.3888(5) 0.2516(3) 0.0506(14) Uani 1 1 d . . . . .
C6 C 0.6005(3) 0.4532(4) 0.3130(3) 0.0432(12) Uani 1 1 d . . . . .
C7 C 0.6118(3) 0.3631(4) 0.4465(3) 0.0443(13) Uani 1 1 d . . . . .
C8 C 0.5591(3) 0.2861(5) 0.4402(3) 0.0500(14) Uani 1 1 d . . . . .
H8 H 0.518541 0.287374 0.401417 0.060 Uiso 1 1 calc R U . . .
C9 C 0.5661(4) 0.2072(5) 0.4910(3) 0.0526(14) Uani 1 1 d . . . . .
C10 C 0.6253(4) 0.2066(5) 0.5509(3) 0.0557(15) Uani 1 1 d . . . . .
C13 C 0.4548(5) 0.1254(7) 0.4336(5) 0.090(3) Uani 1 1 d . . . . .
H13A H 0.426446 0.065548 0.438564 0.135 Uiso 1 1 calc R U . . .
H13B H 0.423192 0.181871 0.439666 0.135 Uiso 1 1 calc R U . . .
H13C H 0.471133 0.127323 0.384456 0.135 Uiso 1 1 calc R U . . .
C12 C 0.6713(3) 0.3599(5) 0.5047(3) 0.0492(14) Uani 1 1 d . . . . .
H12 H 0.707717 0.409979 0.508885 0.059 Uiso 1 1 calc R U . . .
C11 C 0.6777(4) 0.2823(5) 0.5576(4) 0.0581(16) Uani 1 1 d . . . . .
H11 H 0.717349 0.282098 0.597214 0.070 Uiso 1 1 calc R U . . .
C14 C 0.6849(6) 0.1244(9) 0.6600(5) 0.116(4) Uani 1 1 d . . . . .
H14A H 0.679161 0.065997 0.689578 0.174 Uiso 1 1 calc R U . . .
H14B H 0.733072 0.122386 0.641180 0.174 Uiso 1 1 calc R U . . .
H14C H 0.682517 0.182326 0.690957 0.174 Uiso 1 1 calc R U . . .
C15 C 0.6068(4) 0.7163(4) 0.3577(3) 0.0481(14) Uani 1 1 d . . . . .
C16 C 0.6794(4) 0.7560(5) 0.3688(5) 0.0684(18) Uani 1 1 d . . . . .
C17 C 0.6892(6) 0.8581(7) 0.3683(5) 0.094(3) Uani 1 1 d . . . . .
H17 H 0.737700 0.885526 0.375125 0.113 Uiso 1 1 calc R U . . .
C18 C 0.6274(7) 0.9169(6) 0.3580(6) 0.092(3) Uani 1 1 d . . . . .
C19 C 0.5557(6) 0.8798(6) 0.3481(5) 0.087(3) Uani 1 1 d . . . . .
H19 H 0.514156 0.922589 0.341366 0.104 Uiso 1 1 calc R U . . .
C20 C 0.5445(4) 0.7784(5) 0.3482(4) 0.0648(18) Uani 1 1 d . . . . .
H20 H 0.495525 0.752236 0.341904 0.078 Uiso 1 1 calc R U . . .
loop_
 _atom_site_aniso_label
 _atom_site_aniso_U_11
 _atom_site_aniso_U_22
 _atom_site_aniso_U_33
 _atom_site_aniso_U_23
 _atom_site_aniso_U_13
 _atom_site_aniso_U_12
Cl1 0.1167(15) 0.0513(9) 0.0378(8) -0.0042(6) 0.0168(8) 0.0113(9)
O1 0.078(3) 0.059(3) 0.078(3) 0.020(2) -0.002(3) -0.013(2)
N1 0.069(3) 0.039(3) 0.034(2) 0.0004(19) 0.010(2) 0.006(2)
C1 0.051(3) 0.043(3) 0.034(3) 0.002(2) 0.005(2) 0.004(2)
Cl2 0.1340(18) 0.0426(9) 0.0589(10) -0.0080(7) 0.0185(10) 0.0065(9)
O2 0.084(3) 0.076(3) 0.072(3) 0.037(3) -0.011(3) -0.004(3)
N2 0.091(4) 0.048(3) 0.036(3) 0.005(2) 0.012(2) 0.008(3)
C2 0.062(4) 0.044(3) 0.034(3) 0.001(2) 0.007(2) 0.005(3)
Cl3 0.0677(13) 0.1047(19) 0.210(3) 0.027(2) 0.0121(16) 0.0101(13)
N3 0.086(4) 0.057(3) 0.037(3) -0.007(2) 0.009(2) 0.004(3)
C3 0.056(3) 0.042(3) 0.037(3) -0.001(2) 0.006(2) 0.004(3)
Cl4 0.249(5) 0.0417(13) 0.262(5) -0.0046(19) 0.084(4) -0.0229(19)
C4 0.104(5) 0.055(4) 0.037(3) 0.002(3) 0.017(3) 0.007(4)
C5 0.061(4) 0.048(3) 0.042(3) -0.007(3) 0.003(3) 0.000(3)
C6 0.054(3) 0.040(3) 0.036(3) -0.005(2) 0.007(2) 0.000(2)
C7 0.053(3) 0.043(3) 0.037(3) 0.003(2) 0.007(2) 0.011(3)
```

```
C8 0.055(3) 0.049(3) 0.044(3) 0.006(3) 0.002(3) 0.007(3)
C9 0.058(4) 0.049(3) 0.050(3) 0.006(3) 0.004(3) 0.001(3)
C10 0.061(4) 0.055(4) 0.050(3) 0.013(3) 0.005(3) 0.008(3)
C13 0.085(5) 0.068(5) 0.108(7) 0.011(5) -0.019(5) -0.018(4)
C12 0.055(3) 0.047(3) 0.044(3) 0.001(3) -0.001(3) 0.002(3)
C11 0.064(4) 0.058(4) 0.048(3) 0.007(3) -0.008(3) 0.009(3)
C14 0.119(8) 0.132(9) 0.086(6) 0.058(6) -0.027(6) -0.005(7)
C15 0.067(4) 0.039(3) 0.039(3) 0.001(2) 0.008(3) 0.001(3)
C16 0.072(4) 0.056(4) 0.076(5) -0.001(3) 0.006(4) -0.004(3)
C17 0.117(7) 0.066(5) 0.097(6) -0.002(5) 0.010(6) -0.031(5)
C18 0.134(8) 0.039(4) 0.107(7) 0.003(4) 0.034(6) 0.001(5)
C19 0.113(7) 0.050(4) 0.100(6) -0.001(4) 0.021(5) 0.022(5)
C20 0.078(5) 0.047(4) 0.069(4) 0.000(3) 0.006(4) 0.008(3)
_geom_special_details
;
 All esds (except the esd in the dihedral angle between two l.s.
planes)
 are estimated using the full covariance matrix.  The cell esds are
taken
 into account individually in the estimation of esds in distances,
angles
 and torsion angles; correlations between esds in cell parameters are
only
 used when they are defined by crystal symmetry.  An approximate
(isotropic)
 treatment of cell esds is used for estimating esds involving l.s.
planes.
loop_
 _geom_bond_atom_site_label_1
 _geom_bond_atom_site_label_2
 _geom_bond_distance
 _geom_bond_site_symmetry_2
 _geom_bond_publ_flag
Cl1 C2 1.698(5) . ?
O1 C9 1.369(8) . ?
O1 C13 1.394(9) . ?
N1 C3 1.367(7) . ?
N1 C2 1.387(7) . ?
N1 C15 1.429(7) . ?
C1 C2 1.366(8) . ?
C1 C6 1.454(7) . ?
C1 C7 1.475(8) . ?
Cl2 C5 1.717(6) . ?
O2 C10 1.365(8) . ?
O2 C14 1.400(9) . ?
N2 C4 1.315(8) . ?
N2 C3 1.339(7) . ?
Cl3 C16 1.719(8) . ?
N3 C5 1.324(8) . ?
N3 C4 1.343(9) . ?
C3 C6 1.409(8) . ?
Cl4 C18 1.739(8) . ?
```

```
C4 H4 0.9300 . ?
C5 C6 1.403(8) . ?
C7 C12 1.380(8) . ?
C7 C8 1.391(9) . ?
C8 C9 1.394(8) . ?
C8 H8 0.9300 . ?
C9 C10 1.395(9) . ?
C10 C11 1.374(9) . ?
C13 H13A 0.9600 . ?
C13 H13B 0.9600 . ?
C13 H13C 0.9600 . ?
C12 C11 1.404(8) . ?
C12 H12 0.9300 . ?
C11 H11 0.9300 . ?
C14 H14A 0.9600 . ?
C14 H14B 0.9600 . ?
C14 H14C 0.9600 . ?
C15 C20 1.379(9) . ?
C15 C16 1.384(9) . ?
C16 C17 1.385(11) . ?
C17 C18 1.345(13) . ?
C17 H17 0.9300 . ?
C18 C19 1.357(14) . ?
C19 C20 1.380(11) . ?
C19 H19 0.9300 . ?
C20 H20 0.9300 . ?
loop_
 _geom_angle_atom_site_label_1
 _geom_angle_atom_site_label_2
 _geom_angle_atom_site_label_3
 _geom_angle
 _geom_angle_site_symmetry_1
 _geom_angle_site_symmetry_3
 _geom_angle_publ_flag
C9 O1 C13 118.4(5) . . ?
C3 N1 C2 106.7(5) . . ?
C3 N1 C15 124.7(5) . . ?
C2 N1 C15 127.2(5) . . ?
C2 C1 C6 103.9(5) . . ?
C2 C1 C7 125.5(5) . . ?
C6 C1 C7 130.6(5) . . ?
C10 O2 C14 117.7(6) . . ?
C4 N2 C3 112.7(5) . . ?
C1 C2 N1 112.8(5) . . ?
C1 C2 Cl1 128.9(4) . . ?
N1 C2 Cl1 118.2(4) . . ?
C5 N3 C4 116.9(5) . . ?
N2 C3 N1 124.6(5) . . ?
N2 C3 C6 126.5(5) . . ?
N1 C3 C6 108.9(5) . . ?
N2 C4 N3 128.4(6) . . ?
N2 C4 H4 115.8 . . ?
```

N3 C4 H4 115.8 . . ?
N3 C5 C6 122.5(6) . . ?
N3 C5 Cl2 116.4(4) . . ?
C6 C5 Cl2 121.1(5) . . ?
C5 C6 C3 113.0(5) . . ?
C5 C6 C1 139.3(5) . . ?
C3 C6 C1 107.6(5) . . ?
C12 C7 C8 118.5(5) . . ?
C12 C7 C1 119.5(5) . . ?
C8 C7 C1 121.9(5) . . ?
C7 C8 C9 120.9(6) . . ?
C7 C8 H8 119.6 . . ?
C9 C8 H8 119.6 . . ?
O1 C9 C8 125.3(6) . . ?
O1 C9 C10 114.9(5) . . ?
C8 C9 C10 119.8(6) . . ?
O2 C10 C11 124.6(6) . . ?
O2 C10 C9 115.8(6) . . ?
C11 C10 C9 119.6(6) . . ?
O1 C13 H13A 109.5 . . ?
O1 C13 H13B 109.5 . . ?
H13A C13 H13B 109.5 . . ?
O1 C13 H13C 109.5 . . ?
H13A C13 H13C 109.5 . . ?
H13B C13 H13C 109.5 . . ?
C7 C12 C11 121.0(6) . . ?
C7 C12 H12 119.5 . . ?
C11 C12 H12 119.5 . . ?
C10 C11 C12 120.0(6) . . ?
C10 C11 H11 120.0 . . ?
C12 C11 H11 120.0 . . ?
O2 C14 H14A 109.5 . . ?
O2 C14 H14B 109.5 . . ?
H14A C14 H14B 109.5 . . ?
O2 C14 H14C 109.5 . . ?
H14A C14 H14C 109.5 . . ?
H14B C14 H14C 109.5 . . ?
C20 C15 C16 119.9(6) . . ?
C20 C15 N1 120.2(6) . . ?
C16 C15 N1 119.9(6) . . ?
C15 C16 C17 119.8(8) . . ?
C15 C16 Cl3 119.6(5) . . ?
C17 C16 Cl3 120.6(7) . . ?
C18 C17 C16 119.0(9) . . ?
C18 C17 H17 120.5 . . ?
C16 C17 H17 120.5 . . ?
C17 C18 C19 122.3(8) . . ?
C17 C18 Cl4 118.2(8) . . ?
C19 C18 Cl4 119.5(8) . . ?
C18 C19 C20 119.8(8) . . ?
C18 C19 H19 120.1 . . ?
C20 C19 H19 120.1 . . ?

```
C15 C20 C19 119.2(8) . . ?
C15 C20 H20 120.4 . . ?
C19 C20 H20 120.4 . . ?
_refine_diff_density_max    2.084
_refine_diff_density_min   -0.743
_refine_diff_density_rms    0.098
_shelx_res_file
TITL solve4_a.res in C2/c
    solve4_sq.res
    created by SHELXL-2017/1 at 12:19:03 on 15-Jun-2022
REM Old TITL solve4 in C2/c
REM SHELXT solution in C2/c
REM R1 0.285,  Rweak 0.049,  Alpha 0.041,  Orientation as input
REM Formula found by SHELXT:  C21 Cl3 N5 O
CELL  1.54178  17.7407  13.4659  17.8663   90.000   97.594   90.000
ZERR    8.000   0.0016   0.0011   0.0016    0.000    0.005    0.000
LATT  7
SYMM -X, Y, 1/2-Z
SFAC C H CL N O
UNIT 160 104 32 24 16
LIST 4 ! automatically inserted. Change 6 to 4 for CHECKCIF!!
TEMP 23.000
SIZE 0.06 0.12 0.29
L.S. 15
BOND $H
FMAP 2
ACTA
PLAN 20
ABIN
WGHT    0.121200   32.468304
FVAR       0.06082
CL1   3    0.601401    0.594609    0.503605    11.00000    0.11667
0.05132 =
         0.03782   -0.00423    0.01676    0.01126
O1    5    0.518143    0.127296    0.488733    11.00000    0.07820
0.05913 =
         0.07841    0.02047   -0.00222   -0.01349
N1    4    0.596788    0.610989    0.355081    11.00000    0.06908
0.03881 =
         0.03384    0.00040    0.00968    0.00637
C1    1    0.604406    0.448850    0.394726    11.00000    0.05101
0.04274 =
         0.03439    0.00187    0.00506    0.00427
CL2   3    0.611392    0.262726    0.264651    11.00000    0.13398
0.04264 =
         0.05890   -0.00800    0.01851    0.00654
O2    5    0.626322    0.128033    0.599323    11.00000    0.08436
0.07560 =
         0.07188    0.03689   -0.01142   -0.00366
N2    4    0.594582    0.590006    0.221665    11.00000    0.09080
0.04806 =
         0.03591    0.00468    0.01223    0.00756
```

```
C2    1    0.601820    0.546154    0.415895    11.00000    0.06248
0.04388 =
         0.03400    0.00054    0.00732    0.00548
CL3   3    0.756671    0.678035    0.382536    11.00000    0.06768
0.10469 =
         0.21013    0.02746    0.01206    0.01007
N3    4    0.600213    0.421037    0.181315    11.00000    0.08645
0.05653 =
         0.03703   -0.00688    0.00855    0.00440
C3    1    0.597066    0.554046    0.291806    11.00000    0.05554
0.04233 =
         0.03697   -0.00129    0.00633    0.00428
CL4   3    0.641108    1.044674    0.355484    11.00000    0.24943
0.04171 =
         0.26246   -0.00458    0.08357   -0.02289
C4    1    0.595806    0.519683    0.170736    11.00000    0.10414
0.05481 =
         0.03653    0.00170    0.01691    0.00685
AFIX  43
H4    2    0.593293    0.541061    0.120972    11.00000   -1.20000
AFIX   0
C5    1    0.603124    0.388752    0.251629    11.00000    0.06086
0.04787 =
         0.04198   -0.00651    0.00276    0.00015
C6    1    0.600474    0.453158    0.312967    11.00000    0.05393
0.03972 =
         0.03633   -0.00464    0.00735   -0.00009
C7    1    0.611796    0.363109    0.446546    11.00000    0.05304
0.04326 =
         0.03700    0.00304    0.00702    0.01053
C8    1    0.559071    0.286138    0.440161    11.00000    0.05508
0.04935 =
         0.04415    0.00613    0.00164    0.00682
AFIX  43
H8    2    0.518541    0.287374    0.401417    11.00000   -1.20000
AFIX   0
C9    1    0.566146    0.207159    0.491045    11.00000    0.05781
0.04869 =
         0.05031    0.00552    0.00368    0.00101
C10   1    0.625291    0.206588    0.550876    11.00000    0.06146
0.05495 =
         0.05004    0.01265    0.00466    0.00844
C13   1    0.454801    0.125359    0.433563    11.00000    0.08468
0.06793 =
         0.10849    0.01121   -0.01919   -0.01783
AFIX  33
H13A  2    0.426446    0.065548    0.438564    11.00000   -1.50000
H13B  2    0.423192    0.181871    0.439666    11.00000   -1.50000
H13C  2    0.471133    0.127323    0.384456    11.00000   -1.50000
AFIX   0
C12   1    0.671287    0.359919    0.504691    11.00000    0.05467
0.04685 =
```

```
          0.04424    0.00093   -0.00064    0.00232
AFIX  43
H12   2    0.707717    0.409979    0.508885    11.00000   -1.20000
AFIX   0
C11   1    0.677662    0.282281    0.557602    11.00000    0.06401
0.05838 =
          0.04790    0.00661   -0.00762    0.00900
AFIX  43
H11   2    0.717349    0.282098    0.597214    11.00000   -1.20000
AFIX   0
C14   1    0.684867    0.124400    0.660029    11.00000    0.11905
0.13246 =
          0.08620    0.05785   -0.02730   -0.00466
AFIX  33
H14A  2    0.679161    0.065997    0.689578    11.00000   -1.50000
H14B  2    0.733072    0.122386    0.641180    11.00000   -1.50000
H14C  2    0.682517    0.182326    0.690957    11.00000   -1.50000
AFIX   0
C15   1    0.606825    0.716285    0.357671    11.00000    0.06724
0.03884 =
          0.03860    0.00065    0.00802    0.00066
C16   1    0.679404    0.756036    0.368790    11.00000    0.07175
0.05615 =
          0.07637   -0.00072    0.00627   -0.00441
C17   1    0.689165    0.858103    0.368342    11.00000    0.11716
0.06624 =
          0.09721   -0.00220    0.00984   -0.03139
AFIX  43
H17   2    0.737700    0.885526    0.375125    11.00000   -1.20000
AFIX   0
C18   1    0.627420    0.916921    0.357956    11.00000    0.13400
0.03943 =
          0.10669    0.00255    0.03356    0.00126
C19   1    0.555655    0.879812    0.348122    11.00000    0.11298
0.05016 =
          0.09980   -0.00109    0.02071    0.02198
AFIX  43
H19   2    0.514156    0.922589    0.341366    11.00000   -1.20000
AFIX   0
C20   1    0.544477    0.778374    0.348182    11.00000    0.07796
0.04686 =
          0.06861   -0.00003    0.00638    0.00791
AFIX  43
H20   2    0.495525    0.752236    0.341904    11.00000   -1.20000
REM #####
AFIX   0
HKLF 4
REM  solve4_a.res in C2/c
REM R1 =  0.0959 for    2752 Fo > 4sig(Fo)  and  0.1165 for all    3627
data
REM    262 parameters refined using      0 restraints
END
```

```
WGHT      0.1201     32.6686
REM Highest difference peak  2.084,  deepest hole -0.743,  1-sigma
level  0.098
Q1    1   0.7354  0.2826  0.6439  11.00000  0.05    2.08
Q2    1   0.5931  1.0380  0.3210  11.00000  0.05    0.64
Q3    1   0.7542  0.4478  0.5092  11.00000  0.05    0.60
Q4    1   0.6497  1.0560  0.3728  11.00000  0.05    0.53
Q5    1   0.6702  0.0574  0.6073  11.00000  0.05    0.48
Q6    1   0.5491  0.5921  0.4678  11.00000  0.05    0.40
Q7    1   0.6840  1.0250  0.3167  11.00000  0.05    0.34
Q8    1   0.4735  0.7396  0.3097  11.00000  0.05    0.28
Q9    1   0.5537  0.2942  0.2761  11.00000  0.05    0.27
Q10   1   0.4838  0.2793  0.3851  11.00000  0.05    0.27
Q11   1   0.5559  0.2555  0.2136  11.00000  0.05    0.27
Q12   1   0.6212  0.7338  0.3278  11.00000  0.05    0.26
Q13   1   0.6624  0.2715  0.3063  11.00000  0.05    0.25
Q14   1   0.6742  0.5838  0.4932  11.00000  0.05    0.25
Q15   1   0.5186  0.2844  0.5212  11.00000  0.05    0.25
Q16   1   0.6056  0.7576  0.3118  11.00000  0.05    0.23
Q17   1   0.6656  0.6130  0.4836  11.00000  0.05    0.23
Q18   1   0.5048  0.2737  0.3413  11.00000  0.05    0.23
Q19   1   0.6777  0.0016  0.6373  11.00000  0.05    0.23
Q20   1   0.6801  0.2221  0.2442  11.00000  0.05    0.22
```
